# Mechanistic Insights Gained by High Spatial Resolution Reactivity Mapping of Homogeneous and Heterogeneous (Electro)Catalysts

**DOI:** 10.1021/acs.chemrev.2c00867

**Published:** 2023-04-10

**Authors:** Shahar Dery, Barak Friedman, Hadar Shema, Elad Gross

**Affiliations:** Institute of Chemistry and The Center for Nanoscience and Nanotechnology, The Hebrew University, Jerusalem 91904, Israel

## Abstract

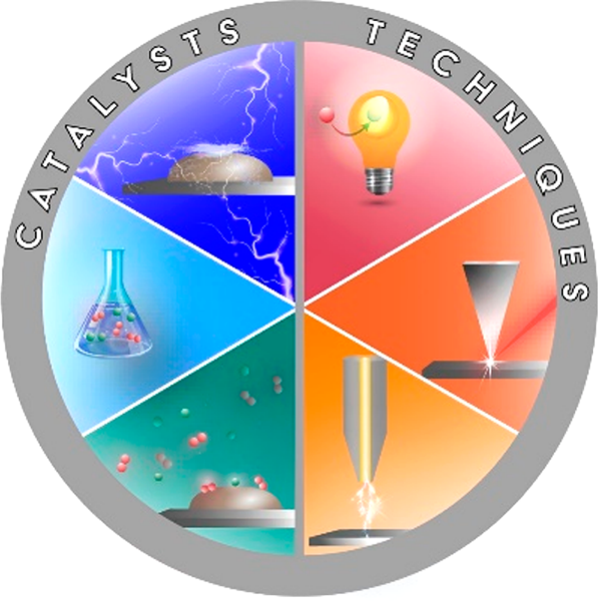

The recent development of high spatial resolution microscopy and spectroscopy tools enabled reactivity analysis of homogeneous and heterogeneous (electro)catalysts at previously unattainable resolution and sensitivity. These techniques revealed that catalytic entities are more heterogeneous than expected and local variations in reaction mechanism due to divergences in the nature of active sites, such as their atomic properties, distribution, and accessibility, occur both in homogeneous and heterogeneous (electro)catalysts. In this review, we highlight recent insights in catalysis research that were attained by conducting high spatial resolution studies. The discussed case studies range from reactivity detection of single particles or single molecular catalysts, inter- and intraparticle communication analysis, and probing the influence of catalysts distribution and accessibility on the resulting reactivity. It is demonstrated that multiparticle and multisite reactivity analyses provide unique knowledge about reaction mechanism that could not have been attained by conducting ensemble-based, averaging, spectroscopy measurements. It is highlighted that the integration of spectroscopy and microscopy measurements under realistic reaction conditions will be essential to bridge the gap between model-system studies and real-world high spatial resolution reactivity analysis.

## 1 Introduction

Heterogeneous (electro)catalysts may appear as a simple material, but a closer look will reveal large nanoscale variances in their structure, composition, and electronic properties.^1−3^ These variations have a dominant impact on the resulting catalytic activity,^4,5^ because different surface sites can modify the adsorption geometries of reactants and intermediates and alter the electron density distribution at a specific surface site, which will in turn modify the affinity toward chemisorption and bond dissociation.^6−9^ Identifying structure–reactivity correlations is therefore central for uncovering the molecular mechanism behind surface-induced catalytic reactions.

Over the past decades, extensive scientific research has focused on probing the ways by which different parameters such as structure, size, composition, and morphology influence the (electro)catalytic reactivity.^10−13^ These studies were mainly based on analysis of the averaged catalytic reactivity of the ensemble and provided fundamental breakthroughs in understanding the origins of surface reactivity.^1,3,14−16^ However, due to the natural inhomogeneity in surface properties and morphology, ensemble-based measurements cannot clearly pinpoint the impact of specific surface sites on the resulting reactivity.

Homogenous catalysts, which are constructed of metal-ion complexes or organic molecules, are expected to have smaller variance and inhomogeneity in their molecular structure and reactivity in comparison to that of heterogeneous catalysts. Recent examples have demonstrated that variations in the molecular structure, location, and accessibility of homogeneous catalysts do exist and impact their reactivity.^17−20^ Therefore, reactivity analysis at the single molecular-catalyst level is also essential for gaining insightful information about the reaction mechanism of homogeneous catalysts.

Various spectroscopic and microscopic tools were developed to reveal the nano-to-atomic structure of catalysts under reactive environments.^4,8,10,21^ Among the techniques that have been widely utilized are scanning transmission X-ray microscopy (STXM), which enables nanoscale X-ray probing with a spatial resolution of ∼10 nm at elevated temperature and pressure.^22^ In addition, X-ray diffraction (XRD) and X-ray nanotomography are high spatial resolution X-ray-based techniques that have been successfully employed to study the composition and oxidation states of catalysts at their reactive state.^9,21,23−27^ X-ray microscopy techniques have been utilized to provide 2D and 3D analysis of catalysts composition at nanometer scale under working conditions.^9,25^ Although these methods provide highly detailed structural information about the catalyst, they deliver limited chemical information about its localized reactivity. Environmental transmission electron microscopy (E-TEM) measurements led to unparalleled subnanometer resolution analysis that visualized surface reconstruction under reaction conditions.^28−33^ However, this method is also limited in its ability to provide chemical information about the localized reactivity pattern. A different approach for single particle reactivity analysis is based on indirect sensing of the influence of particles’ size, structure. and composition by monitoring the localized surface plasmon resonance of single particles.^34^ The combination of single-particle plasmonic nanospectroscopy and mass-spectrometry was utilized to correlate single-particle and ensemble-based reactivity.^35^ These measurements provided kinetic analysis of phase transition mechanism in single metallic NPs under working conditions.^36^

The main restriction of the methods presented thus far is their limited ability to acquire chemical information at the nanoscale that will uncover the nature and distribution of reactants, intermediates, and products.^37−39^ Nanoscale chemical information under working conditions is vital for determining the influence of different sites on the observed catalytic performances of heterogeneous and homogeneous (electro)catalysts.^37^ Probing and tracking reactive sites at nanometer resolution requires high-spatial resolution microscopy and spectroscopy techniques that can dismantle the pattern of the ensemble and provide invaluable information from the perspective of a single catalytic entity.^9,23^ An additional advantage in monitoring the localized catalytic reactivity is the ability to compare it with the averaged reactivity of the ensemble and by that offer vital insights into the reaction mechanism of different surface sites and the influence of the local environment on the resulting reactivity.^40,41^

**Scheme 1:**
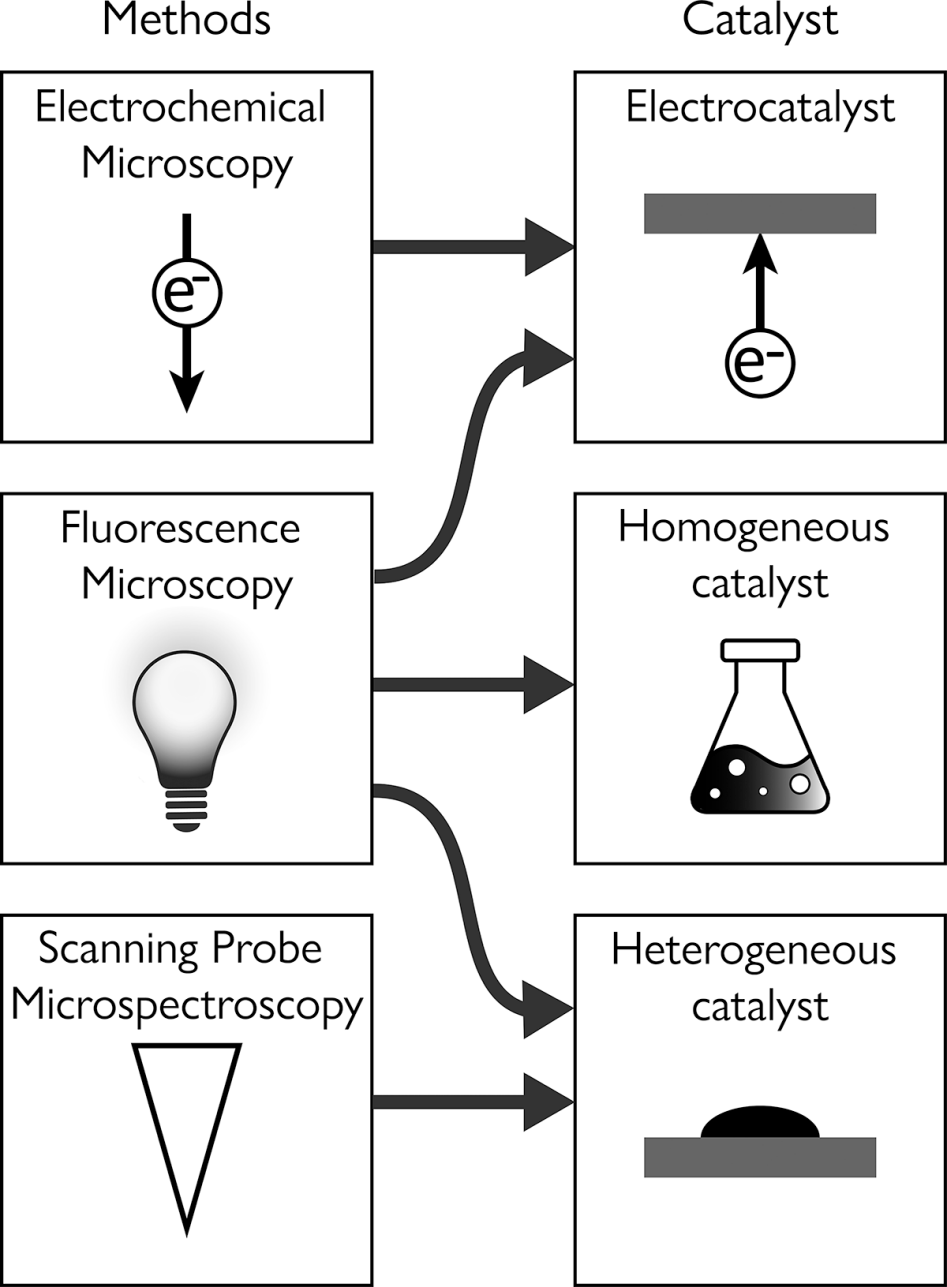
Schematic Description of the Main Analytical Tools and Their Utilization for High Spatial Resolution Reactivity Analysis of Catalytic Systems, Which Are Discussed in This Review Scanning electrochemical microscopy was employed for reactivity analysis of electrocatalysts. The wide versatility of super resolution fluorescence microscopy enabled its utilization for reactivity analysis of homogeneous-, heterogeneous-, and electrocatalysts. Probe-based microspectroscopy was mainly used for reactivity mapping of heterogeneous catalysts.

The aim of this review is to highlight the recent knowledge on the mechanism and reactivity pattern of homogeneous and heterogeneous(electro) catalysts that was gained by using high spatial resolution nanospectroscopy measurements (Scheme 1). This kind of knowledge about the localized mechanism-reactivity correlations could not have been attained by conducting ensemble-based spectroscopy measurements. The main methods that enabled to extract chemical information about nanoscale reactivity and will be the focal point of this study are super-resolution fluorescence microscopy (SRFM), tip-enhanced Raman spectroscopy (TERS), infrared nanospectroscopy, and scanning electrochemical cell microscopy (SECCM). In the following paragraphs, we will briefly describe the different methods and their principle of operation.

## 2 High Spatial Resolution Measurements Tools for Reactivity Analysis

**Scheme 2:**
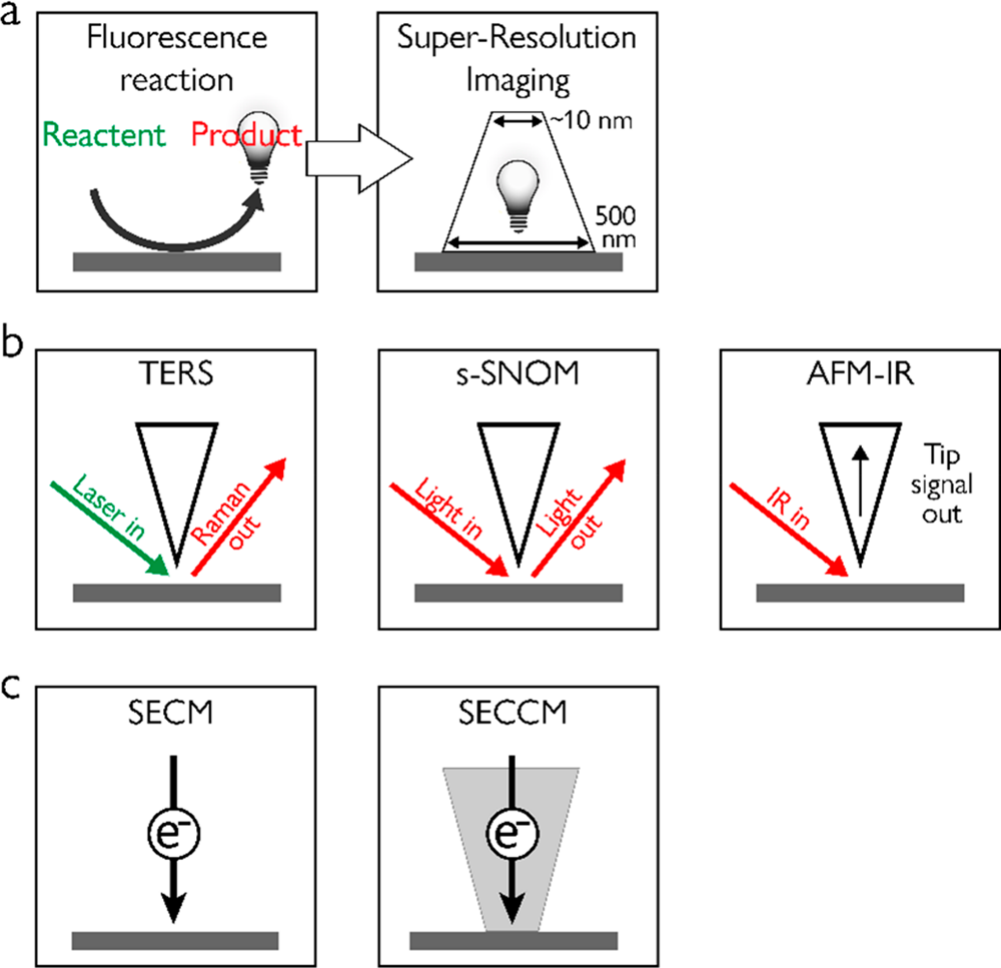
Schematic Description of the Various Analytical Tools for High Spatial Resolution Reactivity Analysis That Are Discussed in This Review^,^^,^ Super resolution fluorescence microscopy, in which catalytic sites are identified by probing fluorescent product molecules, and their exact location is monitored by super resolution imaging. Scanning probe microspectroscopy in which tip–light interactions are utilized to locally enhance the vibrational signals of molecules on surfaces, which are probed by detecting the scattered light or by monitoring changes in tip oscillations induced by photothermal effects. Scanning electrochemical microscopy for reactivity mapping of electrocatalysts either by using a nanoelectrode in scanning electrochemical microscopy (SECM) mode or by using a nanopipette in scanning electrochemical cell microscopy (SECCM) mode.

Super-resolution fluorescence microscopy (SRFM) has been widely utilized to probe the reactivity of catalysts at the nanoscale.^42,43^ This technique is mainly based on detecting the formation of fluorescent product molecules as probes of catalytic sites (Scheme 2a).^44^ In addition, fluorescence tags were attached to reactant molecules and utilized for monitoring polymerization reactions.^17^ The simplicity and high sensitivity of this experimental approach for single catalytic events led to its wide utilization for analysis of homogeneous and heterogeneous (electro)catalysts (Scheme 1).^45^ Although conventional fluorescence microscopy is limited to the resolution of few hundreds of nanometers due to the diffraction limit of light, super-resolution techniques allow images to be acquired with a higher resolution, overcoming the diffraction limit^46^ by isolating emitters and fitting the collected photons to a 2D Gaussian function (Scheme 2a).^47^ This can be practically performed by using low concentration of reactants. Under these conditions, single catalytic events will occur in a range of a few hundred nanometers within the temporal resolution limitations of the measurement and will provide the technical capabilities for reactivity mapping analysis with a spatial resolution of ∼10 nm.

Similarly, the diffraction limit of light restricts the spatial resolution of aperture-based Raman microscopes to a few hundreds of nanometers.^8^ Tip-enhanced Raman spectroscopy (TERS) was developed as a near-field apertureless technique that enables to overcome the diffraction limit of light by focusing the laser source on the apex of an AFM tip to generate a confined surface plasmon at the tip apex, which is positioned in close proximity to the probed surface (Scheme 2b).^48−53^ Thus, the confined plasmon at the tip apex enhances the Raman scattering signal from the surface. The local surface-induced Raman signal can be mapped with high spatial resolution, which is mainly dictated by the diameter of the tip apex. Coating the AFM tip with a plasmonic layer allows for the combination of surface-enhanced Raman scattering (SERS) with the high spatial resolution attained by the AFM tip.^54^ The spatial resolution of AFM is maintained throughout the process, inducing TERS signals with a spatial resolution of ∼20 nm. The ability to identify molecular vibrations at the nanoscale was harnessed for detection of chemical transformations on heterogeneous catalysts.^55−57^ Of note is the recent development of scanning tunneling microscopy (STM) TERS setups that were employed to probe and map the reactivity of catalytic surfaces and demonstrated improved spatial resolution than the one attained by TERS.^58−60^

Fourier transform infrared (FTIR) spectroscopy is a rapid and noninvasive technique used for extracting the chemical fingerprint of organic and inorganic materials. This is also the most dominant vibrational spectroscopy used in the chemical industry and academy alike.^61^ The wavelength of the mid-IR region dictates a diffraction limit for IR microscopes that is in the range of 10 μm.^62^ The microscale resolution enabled IR microscopy measurements to identify progression of catalytic reactions within a flow reactor and detection of chemical reactions within zeolite microcrystals.^63,64^ Nevertheless, the diffraction-limit of light has prevented IR probing in the nanoscale.

Two key IR-based scanning probe microscopy approaches, namely scattering-type scanning near-field optical microscopy (s-SNOM)^65−70^ and atomic force microscopy infrared (AFM-IR),^71−74^ have been developed to surpass the IR diffraction limit and enable the collection of vibrational information with nanoscale resolution (Scheme 2b).^38,39,75^ These two orthogonal approaches combine atomic force microscopy and infrared spectroscopy to provide fast and nondestructive detection of chemically relevant vibrational fingerprint with spatial resolution of ∼20 nm.^65,75^ In s-SNOM IR measurement an IR beam is focused onto the apex of a conductive AFM tip, which acts as a nanoantenna and locally enhances the IR light absorption in the area between the tip apex and the surface.^65−68,76−78^ A confined electric field, originated from tip–surface interactions, yields a localized IR absorption enhancement which is detected by demodulation of the scattered IR light.^67^ Various organic and inorganic materials have been successfully characterized by s-SNOM-IR measurements and the collected near-field signal was comparable with the far-field spectrum.^40,69,79−81^ The chemical contrast and spectroscopic identification of s-SNOM-IR measurements were further improved by the development of synchrotron radiation-based IR nanospectroscopy (SINS) measurements. In this method, a broad, bright and spatially coherent infrared synchrotron radiation was used as a source for IR scattering measurements.^65,66^ SINS measurements were utilized to address fundamental questions in catalysis research by mapping the reactivity pattern on single Au and Pt particles, while using specifically designed probe molecules.^40,41,82−84^

AFM-IR measurements are based on local detection of photothermal expansion by monitoring changes in tip oscillation (Scheme 2b).^71−73^ IR absorption, induced by tunable, pulsed IR laser source, leads to thermal expansion of the substrate, which in turn applies a localized force on the AFM tip that changes its oscillation amplitude. The photothermal effect is mostly influenced by the intrinsic thermal properties of the sample. AFM-IR technique has shown great utility for studying soft materials such as polymers, thin films, and composite-based layers.^38,75^ In a similar way to AFM-IR, photoinduced force microscopy (PiFM) is a highly sensitive and spatially resolved near-field IR technique that uses an AFM tip and IR light to extract surface IR vibrations.^85−87^ PiFM detects the motion of the AFM tip induced by the sample–tip interaction force. The expansion-modulated van der Waals force has been proposed as the dominant factor for vibrational detection in PiFM operation and is proportional to the thermal expansion of the sample under illumination.^88,89^ Thus, in both AFM-IR and PiFM techniques, the IR vibrations are monitored by changes in the tip oscillation pattern that are induced by photothermal expansion, which make these methods highly suitable for analysis of heterogeneous catalysts. It should be noted that due to the relatively long acquisition time required for probing the vibrational signal of molecules on surfaces, in most examples surface anchored probe molecules are used for reactivity detection and thus only a single turnover can be measured on reactive sites.

Scanning electrochemical microscopy (SECM) measurements employ a nanoscale electrode tip to provide quantitative evaluation of the connection between electrocatalytic activity and structure or composition variations (Scheme 2c).^90,91^ Scanning electrochemical cell microscopy (SECCM) replaced the probing electrode of SECM with an electrode-connected nanopipette that forms a local and portable electrochemical cell that functions as an electrochemical probe. SECCM has been demonstrated as a powerful technique for elucidating structure–activity correlations in a wide range of electrochemically active nanomaterials with submicrometer spatial resolution.^92^ Main advantages of SECCM over SECM are its fast electrochemical characterization owing to small capacitive current and its ability to prevent sample contamination during scanning.^93^ The SECCM probe is a micropipette or a nanopipette that is filled with an electrolyte solution and containing quasi-reference counter electrode. The pipet makes meniscus contact with the surface of interest, creating a confined electrochemical cell at the tip of the probe. Localized electrochemical measurements are conducted at a series of points across the surface to construct a spatially resolved map of electrochemical reactivity. Arrays of measurement points can be attained either by a constant distance or by a hopping scanning regime, in which the meniscus is completely detached from the surface after each measurement. Because the contact-area (droplet footprint) is well-defined, this approach provides a highly localized and quantitative electrochemical information.^94^

The aim of this review is to highlight recent knowledge about the reaction mechanism of homogeneous-, heterogeneous- and electrocatalysts that was gained by conducting high spatial resolution measurements while using the above-described microscopy and spectroscopy setups. In the first part of the review, we will discuss the influence of different surface sites on the reactivity (section 3.1) and product selectivity (section 3.2). This will be followed by analysis of the impact of different facets within a single nanocrystal (section 3.3) and composition variations (section 3.4) on the catalytic reactivity. The influence of diffusion of reactants and products to and from the active sites will be discussed (section 4). A central part of this review will focus on novel insights about reaction mechanism that were achieved by high spatial resolution reactivity measurements (section 5). In the last part of the review (section 6), we will conduct a comparative analysis of the different high-resolution tools for reactivity measurements and discuss open questions and opportunities in this field. Due to the limited length of this review, we have mostly discussed literature examples that were published in the last 5 years.

## 3 Site-Dependent Reactivity Analysis

### 3.1 Structure-Dependent Reactivity

This section includes mechanistic insights identified by probing reactivity heterogeneities of catalysts while using the high spatial resolution spectroscopic tools described above. Although the heterogeneity of active sites is a well-known phenomenon, it is difficult to pinpoint and clarify the contributions of different surface sites to the overall reactivity. As will be described below, high spatial resolution measurements provided the ability to directly differentiate and quantitatively correlate between the local catalytic reactivity and the nanometric physicochemical properties of heterogeneous catalysts.

#### 3.1.1 Thermal Catalysis

**Figure 1:**
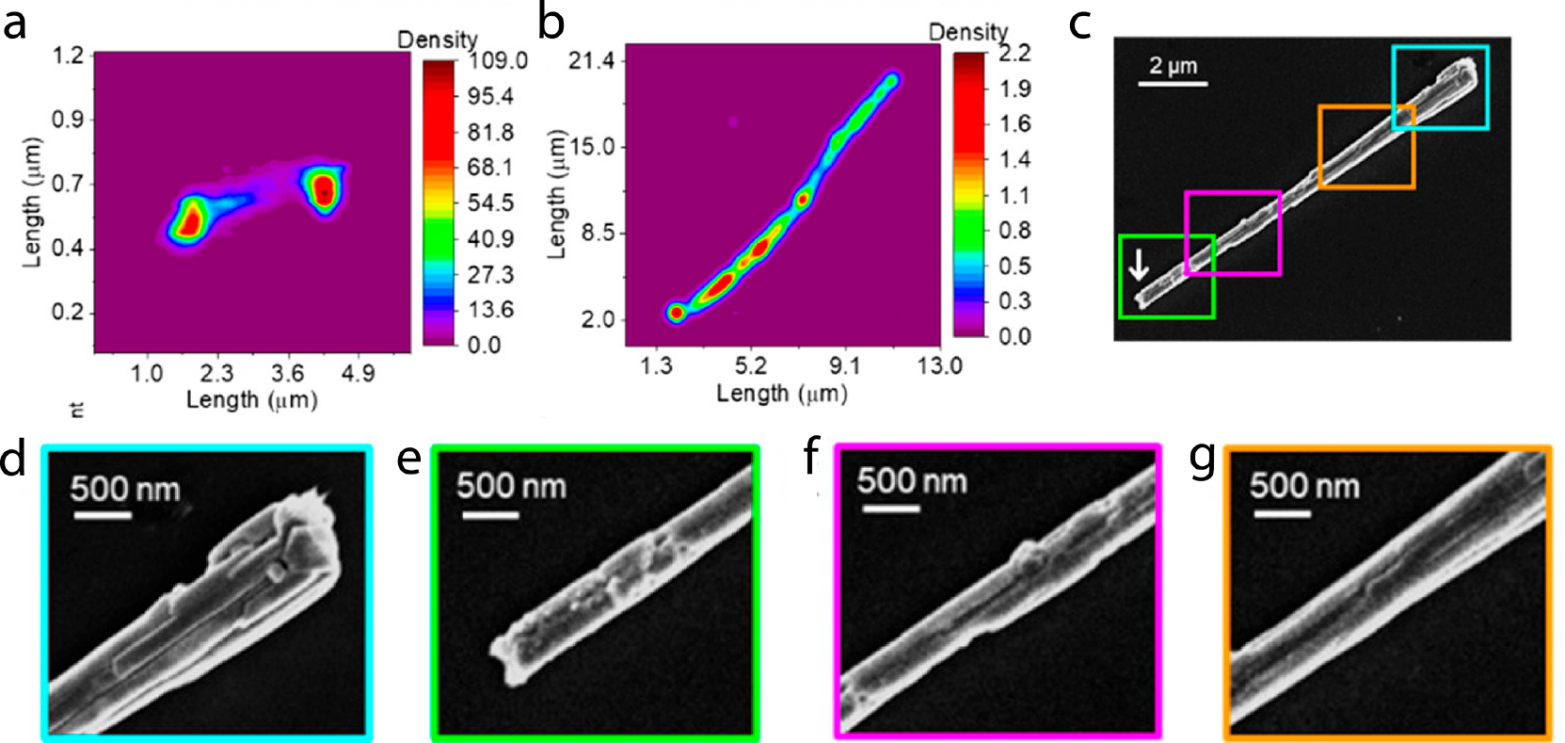
(a) 2D density fluorescence histogram image of short Cu nanowire and (b) long nanowire. (c) SEM images of the long nanowire and (d–g) higher magnitude SEM images of the segments marked by the colored rectangles in c. Adapted with permission from ref (95). Copyright 2020 American Chemical Society.

This subsection focuses on recently gained insights into thermal catalytic processes, which were achieved by employing high spatial resolution techniques. The first example discusses nanoscale catalytic heterogeneity within single copper nanowires that was identified by conducting super-resolution fluorescence microscopy measurements.^95^ The catalytic formation of CalFluor647-triazole from CalFluor647-azide served as the florescent probe reaction to monitor catalytic sites. 2D density plots of the fluorescence signal along a single nanowire (Figure 1a) revealed higher catalytic activity at the edges of the wire and lower activity at its center. Fluorescence signal that was measured for a longer nanowire (Figure 1b) showed a reactivity gradient along the wire. SEM images of the long nanowire and its segments (Figure 1c–g) correlated the locally enhanced reactivity with higher density of surface defects, such as steps, kinks, and corners, demonstrating the crucial impact of surface defects on reactivity. In addition, the high spatial resolution measurements provided an important observation on a region at the center of the rod, a so-called “seed”, from which the rod expands along the *Z*-direction. This region exhibits unexpectedly high reactivity, implying on a considerable number of surface-active sites, an observation that was not attained by previous ensemble-based measurements.

**Figure 2:**
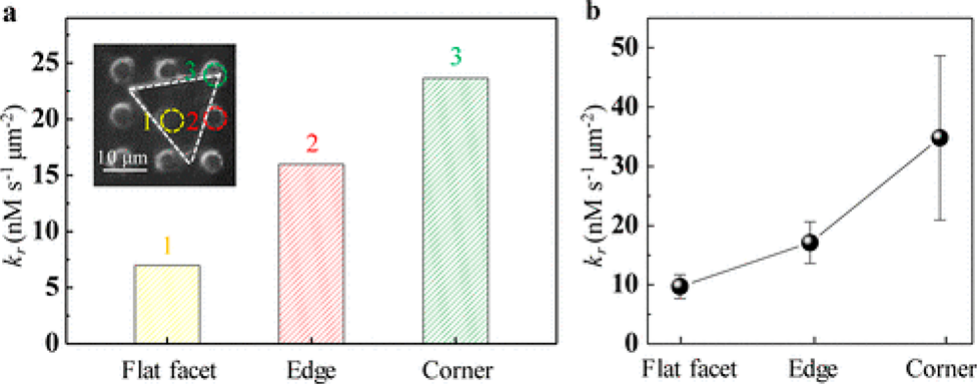
(a) Rate constant calculated for each specific microchamber, marked by a colored circle at the SEM image at the top corner. (b) Averaged rate constants of each region calculated by analysis of 72 Au nanoplates. Adapted with permission from ref (96). Copyright 2017 American Chemical Society.

Site-specific activity and kinetic analysis of Au nanoplates was demonstrated using fluorescence microscopy measurements.^96^ An array of polymethylsiloxane (PDMS) microchambers were imposed on Au nanoplates that served as microreactors for the catalytic reduction of resazurin to resorufin. The location of specific PDMS chambers on the nanoplates allowed fluorescence measurements of specific regions within a single nanoplate. Figure 2a shows the location of three microchambers on a single triangle nanoplate. One of the chambers is located on the flat facet at the center of the plate, the second is on the straight edge of the plates, and the third is on the corner in between two adjacent edges of the triangle. The rate constant, *k*, of each microreactor was calculated by Langmuir–Hinshelwood kinetics and was found to be highest at the corner and lowest at the flat facet. Averaging the rate constants of each region from 72 nanoplates indicated on the same general trend (Figure 2b). These results are consistent with previous reports,^97^ implying on the abundance of low coordinated sites at the corners and edges of the plates. High spatial resolution measurements therefore provided quantitative analysis of reaction rates at different surface sites such as the facet, edges, or corners. The microscale kinetic data offers a unique way to probe reaction kinetics and to understand the averaged kinetic trend that was measured for the ensemble. It should be noted that this method provides only microscale resolution information and therefore irrelevant for catalysts with nanometric dimensions at all directions.

**Figure 3:**
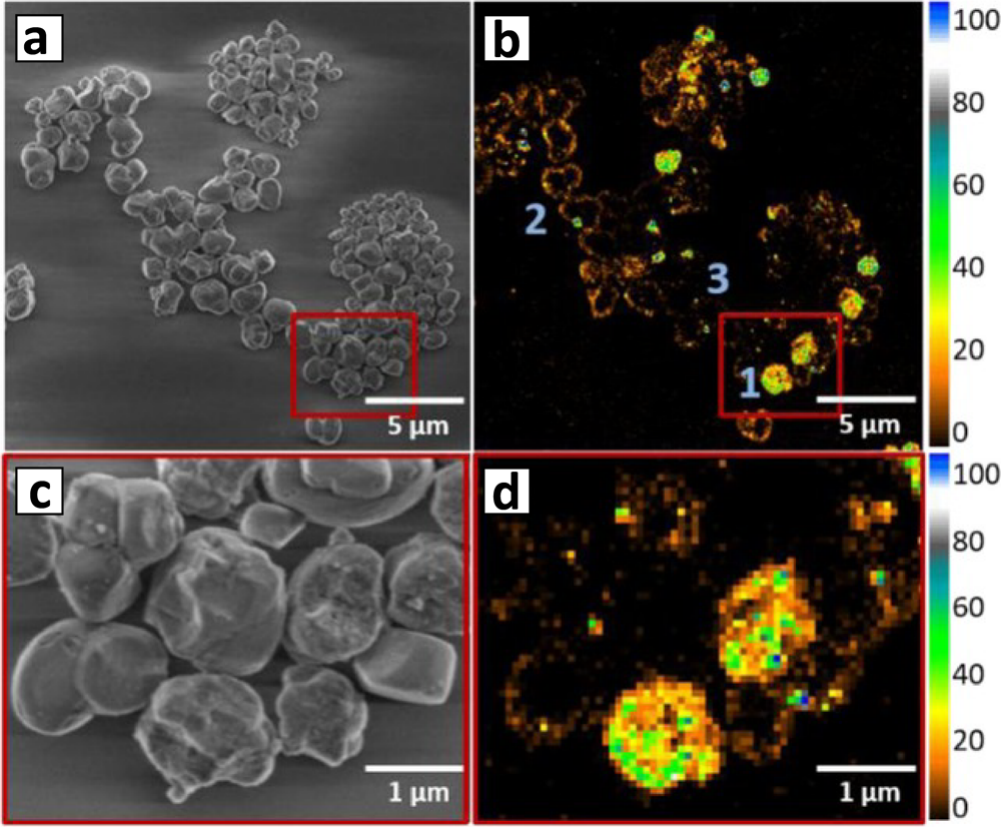
(a) SEM image of H-ZSM-5 particles. (b) 2D fluorescence microscopy mapping of the particles during oligomerization of FFA. The three types of particle marked by 1 (active particles), 2 (active only at the outer surface), and 3 (nonactive). (c,d) Zoomed-in SEM image and reactivity mapping of the areas marked by red rectangle in a and b. Adapted with permission from ref (98). Copyright 2017 Wiley-VCH.

Fluorescence microscopy measurements at higher spatial resolution were performed to probe the distribution of acidic sites within H-ZSM-5 zeolite at the subparticle level with a resolution of ∼20 nm.^98^ Oligomerization of furfuryl alcohol (FFA) served as the fluorescent probe reaction, because it is catalyzed by the Brønsted acid sites of the zeolite. Mapping the fluorescence signals that were obtained from single turnover events revealed three types of particles within the zeolite batch (Figure 3a,b). Some particles showed relatively homogeneous reactivity throughout the entire particle while in others the reactivity was detected along the outer surface of the particle, and nonactive or nearly nonactive particles were probed as well.

Closer look at the mapping results combined with SEM images revealed a clear correlation between surface roughness and catalytic activity. Particles with high degree of roughness showed enhanced reactivity across the particle, whereas particles with deteriorated reactivity were characterized with a smoother surface (Figure 3c,d). The link between structural properties of single zeolite particles and their catalytic activity in terms of quantity and accessibility of acidic sites emphasizes the importance of correlating the electron microscopy images with quantitative reactivity mapping analysis.

**Figure 4:**
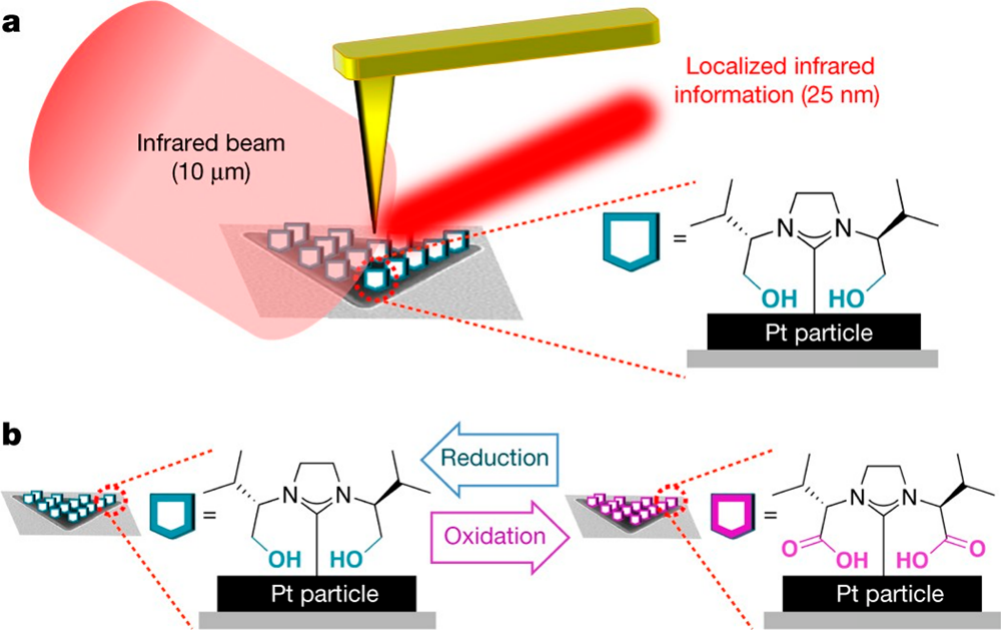
(a) Hydroxyl functionalized NHCs (OH-NHCs, turquoise pentagons) were anchored to the surface of Pt particles (black), which were deposited on a Si wafer (gray). An AFM tip (yellow) acts as an optical antenna by localizing the diffraction-limited synchrotron-sourced infrared beam, inducing an infrared scattering signal with a spatial resolution of ∼25 nm. (b) Under oxidizing conditions, the catalytic Pt particles oxidized the hydroxyl group into carboxylic acid (purple pentagons). This redox is reversible and under reducing conditions the carboxylic acid was reduced back into the hydroxyl group. Adapted with permission from ref (41). Copyright 2017 Nature Publishing Group.

IR nanospectroscopy measurements were conducted to map the surface reactivity pattern on single Pt and Au nanoparticles, while using functionalized N-heterocyclic carbene molecules (NHCs) as reactivity probes (Figure 4).^41^ Pt and Au nanoparticles were prepared on Si (110) wafer and coated with a monolayer of hydroxyl functionalized N-heterocyclic carbene molecules (OH-NHCs) as indicators for oxidation and hydrogenation reactions (Figure 4b).

**Figure 5:**
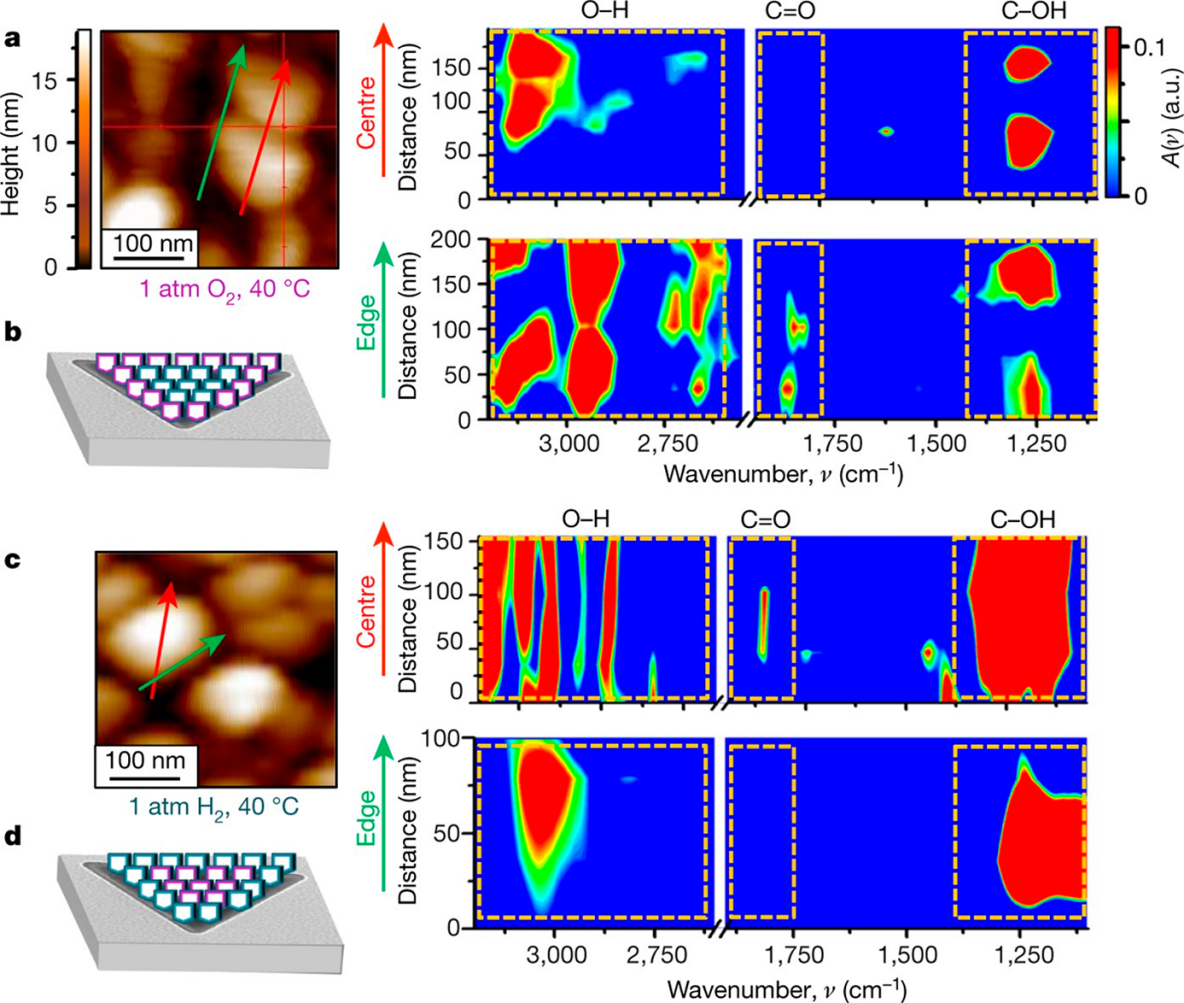
(a,c) IR nanospectroscopy line scans were taken across the center and perimeter (red and green arrows, respectively) of OH-NHC coated Pt particles following exposure of the sample to mild oxidizing (a) and reducing (c) conditions. The dashed highlighted areas in the spectra indicate the CO–H, C=O, and O–H absorption wavenumbers, demonstrating the variations in the IR spectra in these regions following exposure to different reaction conditions. (b,d) Schematics of the NHC-coated Pt particles following their exposure to oxidizing or reducing conditions, respectively. Adapted with permission from ref (41). Copyright 2017 Nature Publishing Group.

IR nanospectroscopy measurements were performed following exposure of Pt NPs coated with OH-NHCs to mild oxidizing conditions (Figure 5a,b) and revealed reactivity variations on different surface sites across the surface of a single Pt particle. The carbonyl (C=O) vibration signature was identified in the IR spectra that was acquired across the particles’ edge, nevertheless, this signal was not detected in IR spectra that was acquired along the particles’ center. These differences indicate that NHCs anchored on edge sites were more prone to initiate a catalytic oxidation reaction than NHCs that were anchored on the center of the particle. Reactivity variations were linked with differences in the coordination number of surface atoms, and enhanced catalytic performance was related to higher density of low-coordinated surface atoms at the particle’s perimeter.

The reversibility of the reaction was demonstrated by exposure of the sample to mild reducing conditions. Line scan mapping revealed the loss of the carbonyl vibration in the IR spectra collected from the perimeter of the particle, correlated to the enhanced reactivity of these surface sites (Figure 5c,d). Thus, the use of IR nanospectroscopy with functionalized NHCs as molecular probes enabled to probe catalytically relevant oxidation and hydrogenation reactions, while overcoming the requirement of fluorogenic products for high spatial resolution reactivity probing.

**Figure 6:**
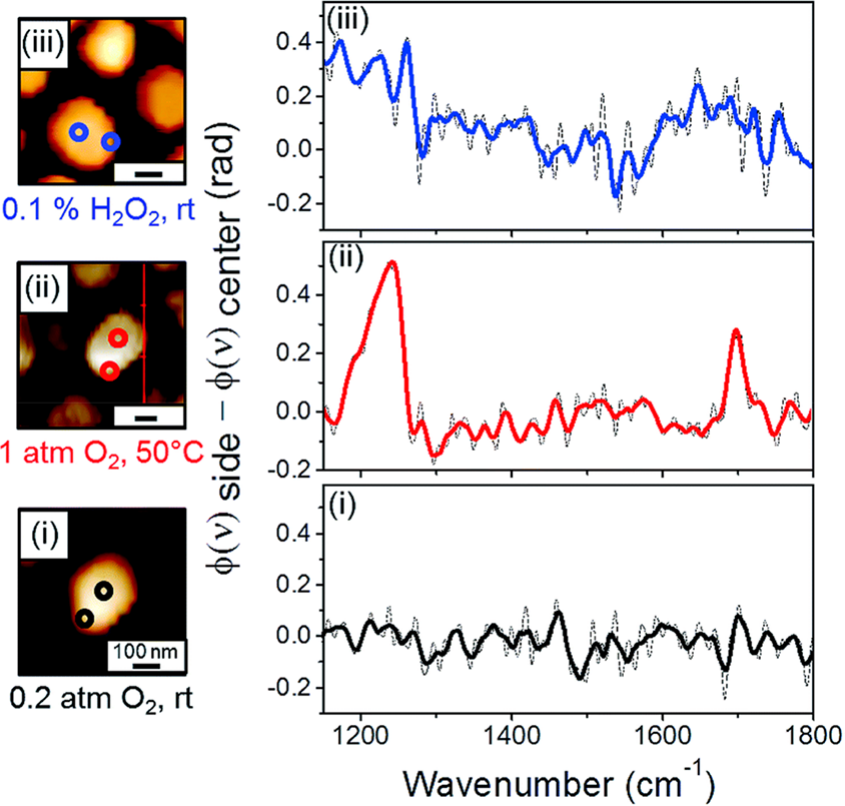
IR nanospectroscopy measurements were conducted at the perimeter and center of single Pt particles coated with OH-NHCs. Colored circles in the AFM topography image represent the location of the IR measurements (left panels). The differences in near-field spectra that were acquired at the perimeter (φ(ν) side) and center (φ(ν) center) of each particle are shown (right panels). IR nanospectroscopy measurements were conducted following NHCs’ surface-anchoring (i) and following exposure of the sample to gas phase (1 atm of O_2_, 50 °C, 10 h) (ii) and liquid phase (0.1% w/w H_2_O_2_, rt, 10 h) (iii) oxidizing conditions. The dash and color lines represent the as-received and smoothed spectra, respectively. Adapted with permission from ref (83). Copyright 2018 Royal Society of Chemistry.

IR nanospectroscopy was further utilized to identify the influence of oxidizing conditions on the reactivity pattern within a single catalytic particle.^83^ OH-NHCs were used as chemical probes for surface-induced oxidation reactions on Pt particles. IR measurements were conducted at different regions within single Pt particles following exposure to various oxidizing conditions. Subtraction of the IR spectra measured at the center of the particle from the spectra that was taken at the edge revealed enhanced reactivity at the perimeter of the particle (Figure 6).

The site-dependent reactivity patterns were highly influenced by reaction conditions, in which milder gas phase oxidation led to enhanced reactivity at the periphery that led to carboxylic acid formation (Figure 6, spectrum (ii)). Higher reactivity was observed following exposure to harsher oxidizing conditions; however, this was coupled with decomposition of the probe molecule (Figure 6, spectrum (iii)), implying that this condition may lead to selective poisoning of highly reactive edges sites.

**Figure 7:**
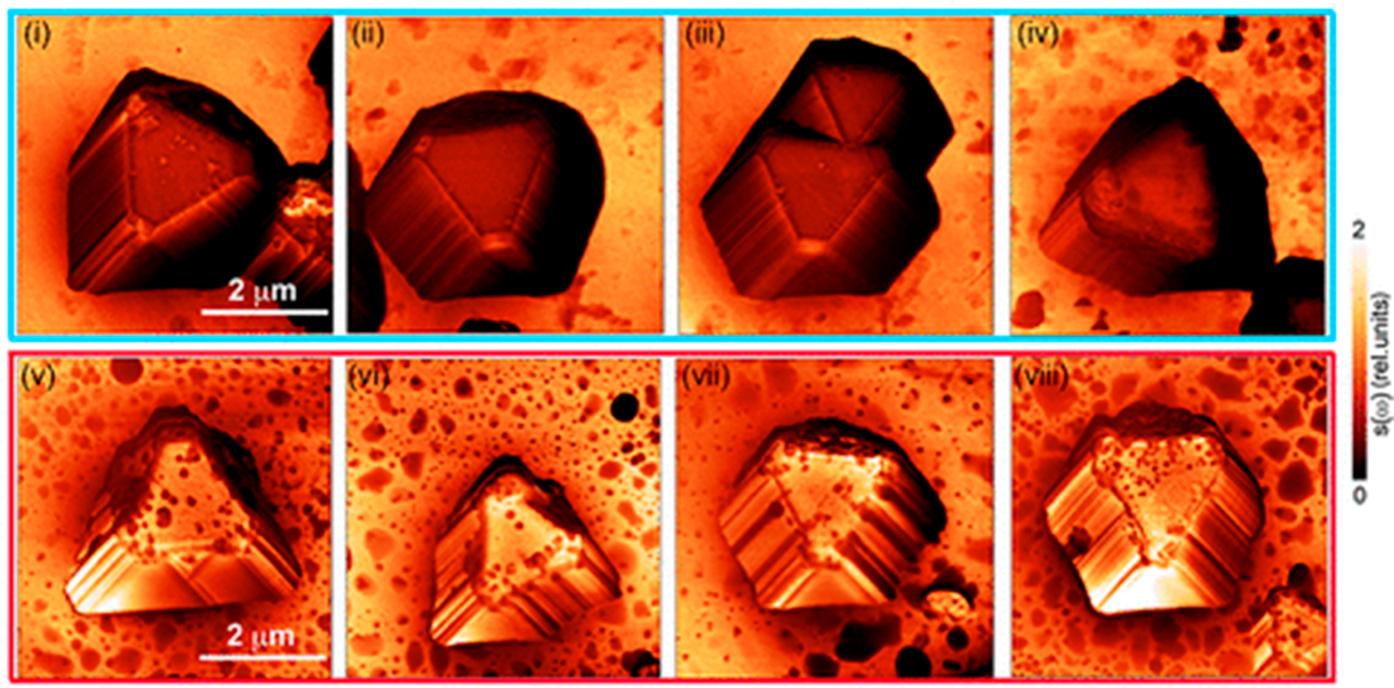
Nano-IR imaging of Cu_2_O microcrystals prior and following electrochemical reactions. Nano-IR amplitude images collected for Cu_2_O microcrystals at 905 cm^–1^. Panels (i–iv) show IR nanospectroscopy mapping of Cu_2_O prior the reaction, whereas panels (v) to (viii) show the mapping following electrochemical reaction in KHCO_3_ solution. Adapted with permission from ref (99). Copyright 2020 American Chemical Society.

Near-field IR mappings provide 2D information about the vibrational signature on particles' surface and were conducted to elucidate the electrocatalytic reactivity pattern of Cu_2_O microcrystals in electrochemical CO_2_ reduction.^99^ Once illuminated by IR signal at 905 cm^–1^ the Cu_2_O polyhedral microcrystals exhibited clear near-field contrast compared with the substrate, correlated to the presence of hydrocarbons. *Ex situ* IR nanospectroscopy mapping revealed that the spent catalyst shows a considerable increase in its near-field signal as compared with its pristine state, which is indicative of chemical residues randomly distributed on the sample surface (Figure 7). The enhanced near-field signal was mainly detected at the corners and edges of the Cu_2_O microcrystal, indicating on a higher reactivity of these surface sites toward electrochemical CO_2_ reduction as compared with its interior sites. This work showcased the ability of IR nanospectroscopy measurements to elucidate deactivation pathways of different nanometric surface sites due to poisoning, information that is typically lost in far-field IR spectroscopy measurements.

**Figure 8:**
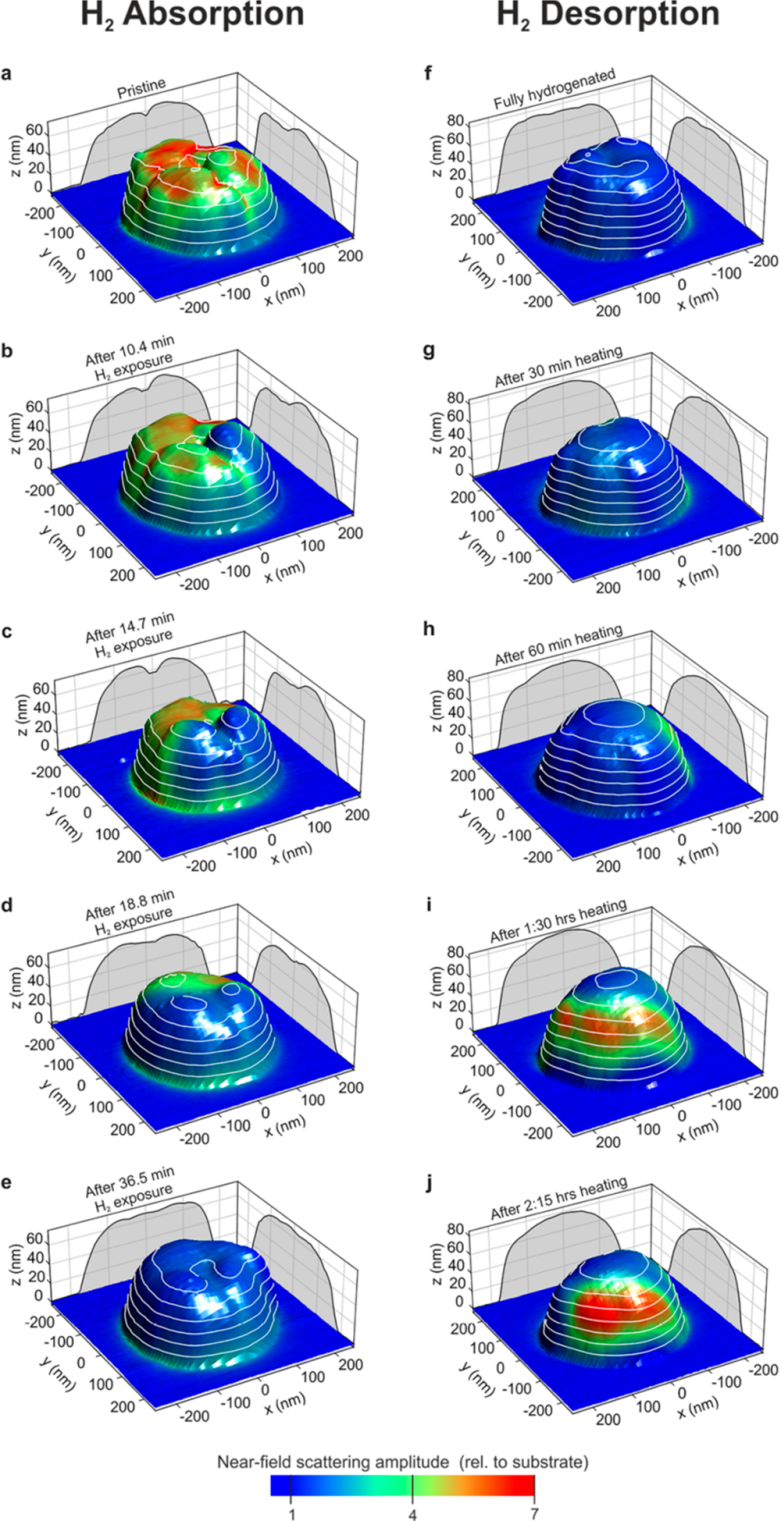
Overlay of the color-coded near-field scattering amplitude on the topography of Mg particle at different stages of hydrogen absorption (a–e) and desorption (f–j). The projected gray areas show the particle contour in the XZ and YZ plane. Adapted with permission from ref (100). Copyright 2018 American Chemical Society.

An elegant approach for *in situ* reactivity analysis was demonstrated by probing the hydrogen sorption in Mg nanoparticles. *In situ* near-field scattering microscopy measurements enabled to spatially and temporally resolve the phase transition of Mg to MgH_2_ by tracking the changes in the light scattering pattern (in the visible range) of single particles.^79,100^Figure 8a–e shows the *in situ* mapping of the near-field signal acquired from a single Mg nanoparticle during hydrogen sorption. The mapping vividly demonstrates the transition of Mg to MgH_2_ by monitoring the diminished near-field signal with time. Moreover, the phase transition occurred in a sequential manner in individual crystalline grains within the particle. A comparison between the fully hydrogenated particle (Figure 8f) to its pristine state (Figure 8a) indicated on a topography change to a smoother particle surface in conjunction with an increase in the particle’s thickness. Commensurately, hydrogen desorption was monitored *in situ* starting from a fully hydrogenated MgH_2_ nanoparticle (Figure 8f–j).

IR nanospectroscopy mapping revealed that hydrogen desorption induced a morphology change into a hemispherical particle. This shape suggested that a local minimum in surface energy was achieved. Interestingly, the dehydrogenated “end point” does not restore the initial near-field response, which implies that the conductivity and plasmonic nature of the particle have been hampered. These results indicated that the phase transition is not a simple continuous process but rather a step-like process that is instigated by reaction of individual grains within the particle. It was deduced that hydrogen diffusion is limited by grain boundaries of individual crystallites, yielding a lag in the hydrogenation process.

**Figure 9:**
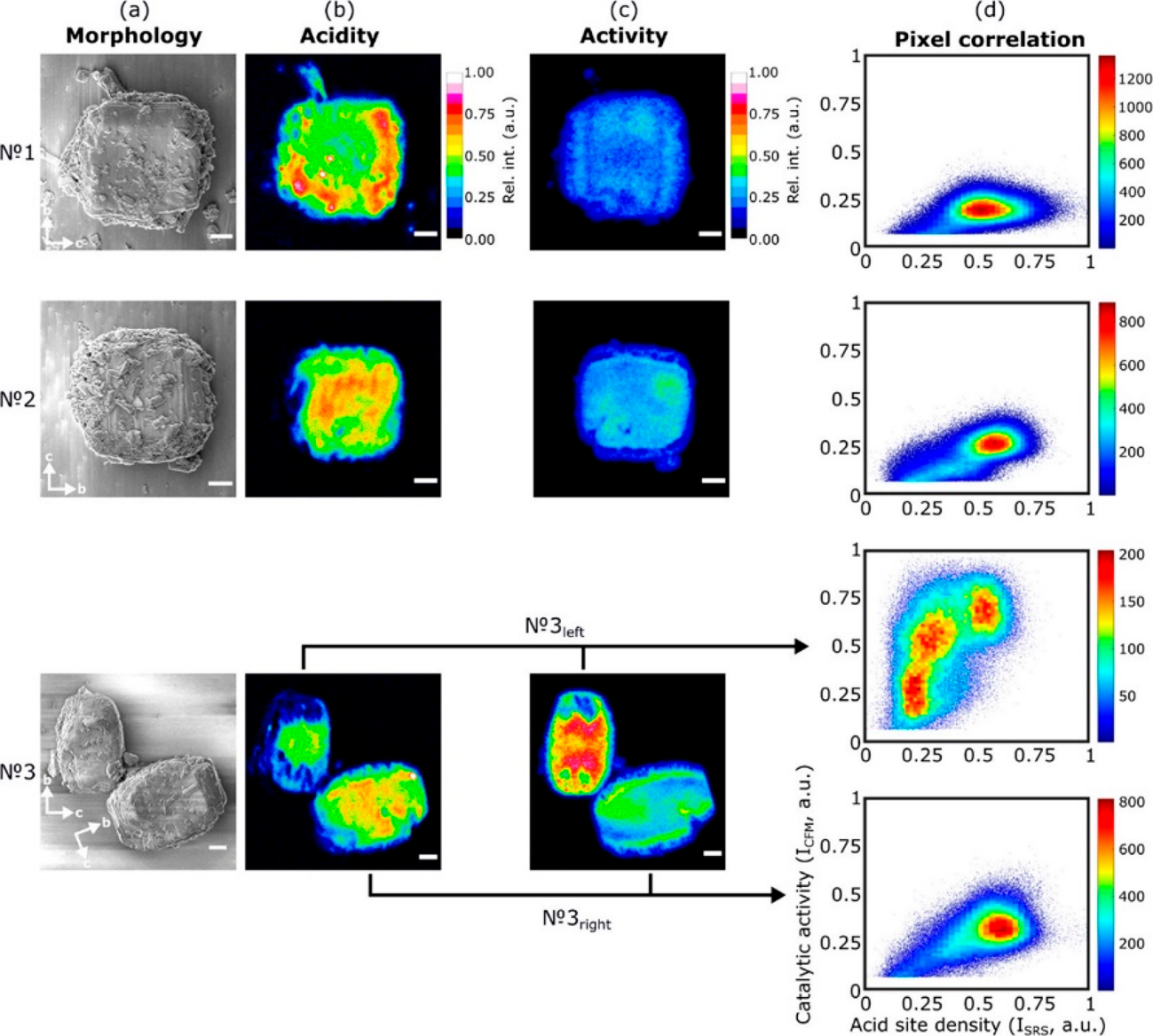
(a) SEM images of dealuminated mordenite crystals (scale bar = 2 μm). (b) Raman images (1006 cm^–1^) of the pyridinium ion in the crystals. (c) Fluorescence microscopy image of the FFA oligomers formed and accumulated within the dealuminated mordenite crystals after 20 min of reaction. (d) Correlation plot of fluorescence microscopy versus Raman scattering signal intensity. The heat map indicates the corresponding number of correlations per bin. Adapted with permission from ref (101). Copyright 2020 American Chemical Society.

Structure–activity correlations are typically used to evaluate the performance of catalysts. However, these correlations are commonly determined by bulk scale measurements, and therefore, a variety of local catalyst properties, such as site accessibility and activity are masked by the ensemble properties. In a recent report, the link between acid sites distribution and catalytic activity were investigated for dealuminated mordenite (ZM-510) acid catalyst at the subparticle scale by Raman microspectroscopy and fluorescence microscopy (Figure 9).^101^

Mordenite catalysts that were exposed to variable degrees of dealumination processes served as the model system. These processes influence both the accessibility and the concentration of acid sites because dealumination reduce the acid site concentration and commensurately raise their accessibility. The distribution of Brønsted acid sites which dominates the catalytic performance of ZM-510 catalyst was mapped by stimulated Raman scattering (SRS) microscopy while using pyridine as a basic probe molecule. In addition, mapping of the catalytic activity was facilitated by utilizing confocal fluorescence microscopy (CFM) to map fluorescent oligomers of furfuryl alcohol (FFA). The connection between acid site distribution and catalytic activity was enabled by pixel-to-pixel nanoscale correlation analysis of SRS and CFM images (Figure 9). As can be inferred, acid sites distribution and reactivity mapping show both intra- and interparticles heterogeneities. The heterogeneous behavior can be explained by local variability in accessibility and density of acid sites. Figure 9d demonstrates the correlation plot between SRS intensity and CFM imaging displaying a clear lack in connection between the acid sites and active sites distribution. The absence of a clear trend is explained by the dependency on molecular transport/diffusion as an essential part of the catalytic process.

The studies in this section demonstrate the crucial impact of high spatial resolution measurements for reactivity analysis on single nanoparticles that enabled to probe the influence of structural, composition, and accessibility variations on localized reactivity pattern. This analysis provided quantitative information about the correlation between local structural variations and their impact on reactivity. In addition, the influence of catalytic reactions on local structural properties uncovered reactivity deactivation mechanisms on single nanoparticles.

#### 3.1.2 Photoinduced Catalysis

**Figure 10:**
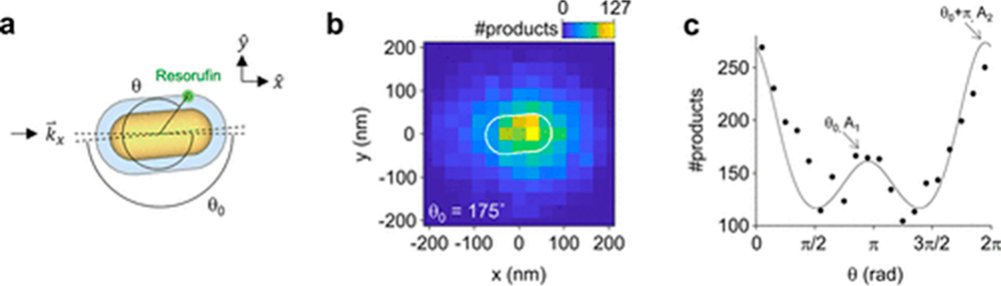
(a) Illustration of the studied system and the angles θ_0_ and θ. (b) 2D histogram of detected fluorescent product on a single nanorod. (c) Histogram of product formation and the angle θ correlation at a signal location. Adapted with permission from ref (102). Copyright 2021 American Chemical Society.

Super-resolution imaging of plasmon-driven chemical reactions on Au nanorods was achieved by *in situ* fluorescence microscopy.^102^ Au nanorods were coated with a shell of mesoporous silica and resazurin reduction to resorufin served as a probe reaction. Mapping of the nanorod electric field distribution was performed upon irradiation with a p-polarized light at 532 nm. The electric field distribution revealed that for p-orientation the field intensity was higher at the tip, located away from the incident beam source, while for the s-orientation, the field intensity was near equal on both tips. Reactivity analysis was conducted for nanorods oriented at different θ_0_ angles, and for the product location which corresponds to an angle θ (angles are defined in Figure 10a). The product mapping (Figure 10b) was acquired with ∼30 nm resolution and enabled assignment of a product to a specific location within the nanorod as demonstrated in a histogram of the product detection at different θ angles (Figure 10c). The data was fitted to two Gaussian peaks, A_1_, which corresponds to the tip away from the beam (in-plane angle of θ_0_), and A_2_, which corresponds to the other tip (in-plane angle of θ_0_ + π). In this manner, a correlation between catalytic activity and the local electric field intensity was probed within a single rod.

**Figure 11:**
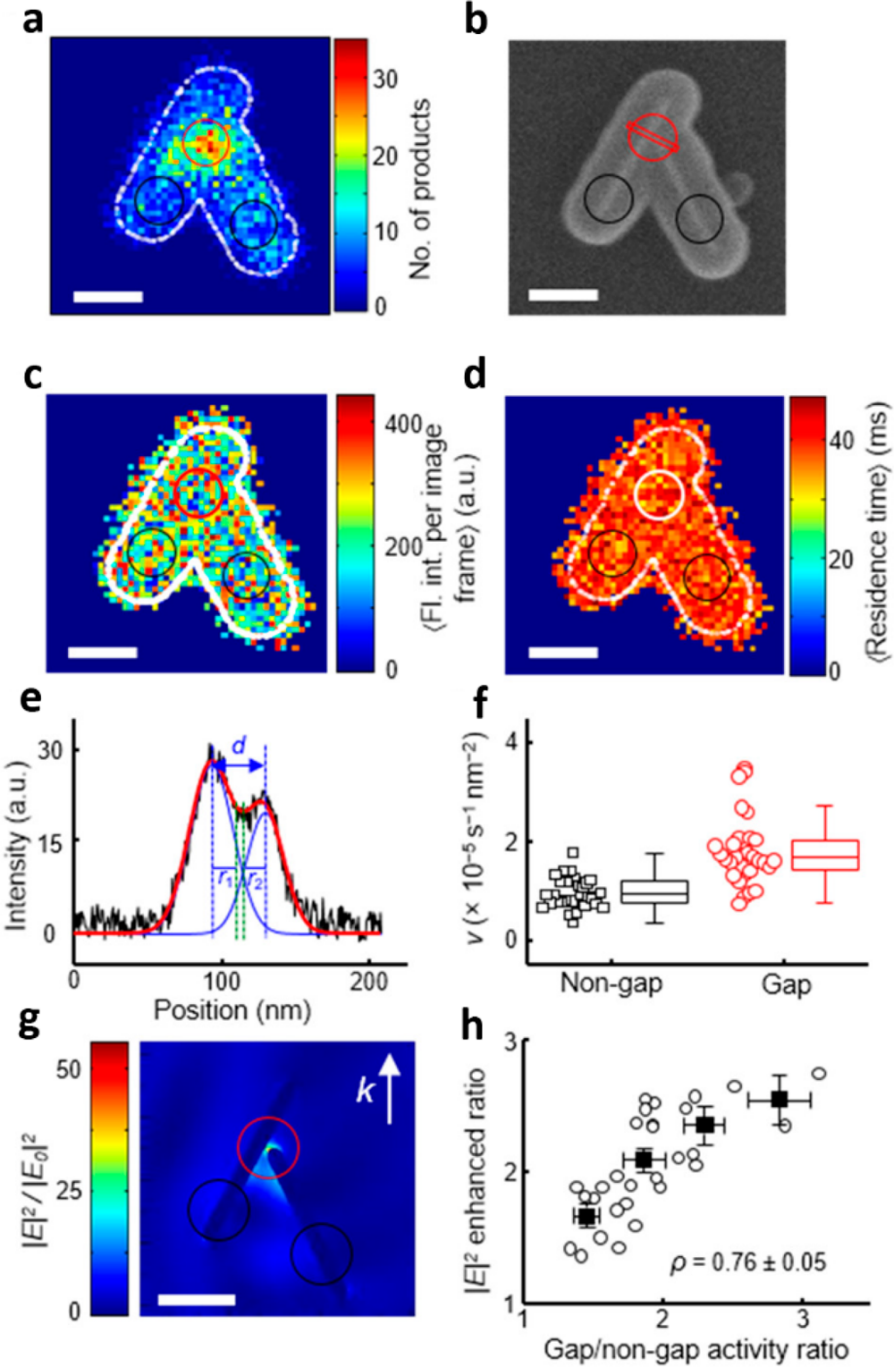
(a) Quantitative mapping of super-resolution fluorescence microscopy of Au nanorods dimer. (b) SEM image of the structure. (c) Spatial distribution of single catalytic product fluorescence intensity. (d) Spatial distribution of averaged residence time of products. (e) Topography profiling of the red line at B, where *d* is the distance between two nanorods. (f) Comparison of calculated turnover rate in gap sites and nongap sites. (g) Electric field enhancement derived from FDTD calculation. (h) Relation between electric field enhancement and reactivity difference of gap and nongap sites. Scale bar = 200 nm. Adapted with permission from ref (103). Copyright 2018 American Chemical Society.

Super-resolution fluorescence microscopy was also utilized to reveal the influence of plasmonic hotspots on photoinduced reaction yield.^103^ The distinctive fluorescent reaction of resazurin to resorufin was used for probing the surface-induced catalytic reactivity on two linked Au nanorods coated with mesoporous silica. Figure 11a,b shows the fluorescence reactivity mapping for a single nanostructure and its corresponding SEM image. The mapping clearly revealed enhanced catalytic activity at the gap between the two nanorods. Furthermore, no distinct difference in the fluorescence intensity from a single product formation over gap and nongap sites was observed nor different product residence time between these sites (Figure 11c,d). This result confirms that the increased signal at the gap in Figure 11a is correlated with an increased number of turnover events (Figure 11f). Finite difference time domain (FDTD) simulations were conducted and show strong correlation between the electric field enhancement and the observed catalytic activity (Figure 11g). Figure 11h demonstrates the plotting of the parameters, indicating that the enhanced catalytic activity is driven by surface plasmon. Thus, super-resolution fluorescence microscopy enabled to quantitatively analyze the local reactivity enhancement induced by hot spots.

The reactivity pattern of plasmon-driven Suzuki–Miyaura cross-coupling reaction was mapped by TERS on gold–palladium bimetallic nanoplates (Au@PdNPs).^104^ Au-coated AFM tip was functionalized with 4-mercaptophenylboronic acid, and the bimetallic surface was functionalized with a monolayer of halogenated derivatives of benzenethiol. The functionalized tip was brought near the bimetallic surface, and upon illumination, a plasmon-induced reaction was initiated at the nanometric region beneath the tip apex that led to the formation of biphenyl-4,4′-dithiol, the product of the studied Suzuki–Miyaura reaction. The edges and corners of the bimetallic nanoplates showed higher reactivity than the flat regions at the center of the nanoplates. Site-dependent reactivity pattern was observed for all halide derivatives that were measured, indicating that the enhanced reactivity of plasmon-induced C–C coupling reactions at defect sites is a general feature of bimetallic Au/Pd nanostructures.

High spatial resolution measurements of plasmon induced reactions on heterogeneous catalysts, which were discussed in this subsection, uncovered a nonhomogeneous reactivity pattern. Quantitative analysis of the impact of hot spots on nanoscale reactivity and the connection between the electric field strength and plasmon induced reactivity pattern were revealed. In addition, the connection between composition variations, which can facilitate the local reactivity, and plasmon induced reactivity were probed by conducting high spatial resolution measurements on multicomponent plasmonic particles. Thus, nanoscale reactivity analysis revealed that surface heterogeneities in plasmon-induced reactions are directly correlated to local structure and composition variations on single nanoparticles.

#### 3.1.3 Electrocatalysis

**Figure 12:**
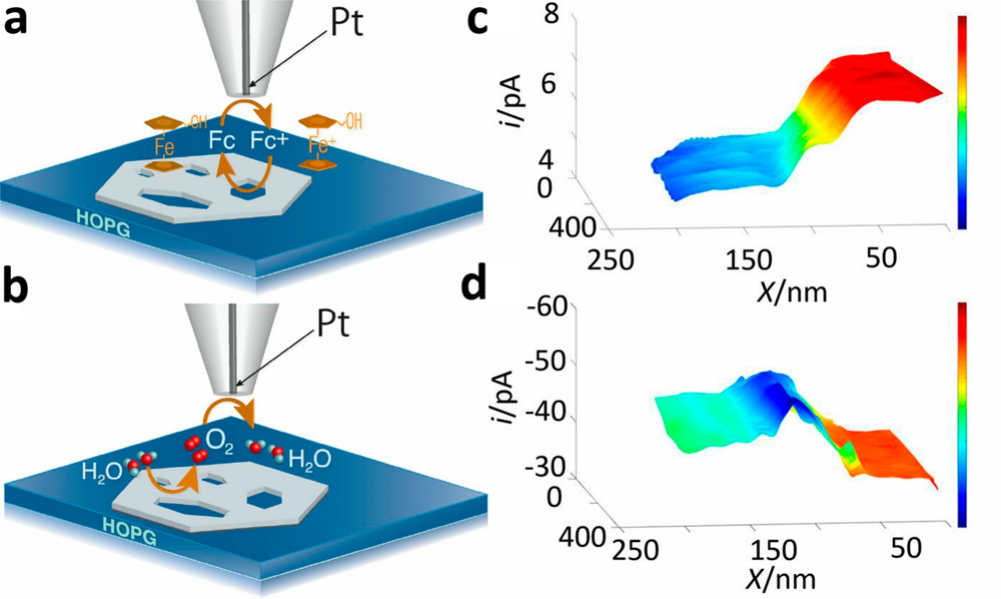
Schematic representation of (a) positive feedback produced by oxidation/reduction of ferrocenemethanol (Fc) and (b) substrate generation/tip collection of O_2_ at NiO nanosheet. SECM imaging of edge site: (c) feedback mode and (d) SECM images of the NiO edge. Pixel density: 330/μm (*x* axis) and 100/μm (*y* axis). Adapted with permission from ref (105). Copyright 2019 Proceedings of the National Academy of Sciences.

*Operando* mapping of the electrocatalytic reactivity was achieved by SECM measurements, which correlated the topography and oxygen evolution reaction (OER) yield of NiO catalyst (Figure 12a,b).^105^ The NiO nanosheet thickness was ∼10–20 nm in a mostly flat morphology bearing hexagonal defects and well-defined edges. This nanosheet was prepared on a highly oriented pyrolytic graphite (HOPG) substrate that is essentially inert to the reaction. Due to these differences in the reactivity pattern, the feedback mode of the SECM could easily differentiate between the topography and composition of the underlying surface. Furthermore, because oxygen is generated solely on the NiO surface, generation/collection mode complemented the feedback mode results, enabling to map the electrocatalytic OER activity. The reactivity on the basal (111) plane, as well as edge and corner sites were compared, and it was identified that the edge sites are considerably more active. To gain a better resolution, 20 nm sized Pt electrode was utilized for mapping the NiO/HOPG transition region (Figure 12c,d). SECM feedback mode mapping displayed a continuous like transition from the inactive HOPG surface to the active NiO as indicated by the lower current (Figure 12c). The complementary mapping conducted by substrate-generation/tip-collection mode (Figure 12d) showed that the transition occurred in the same region at a distance of ∼50–70 nm. Nevertheless, the transition did not appear continuous as in Figure 12c but rather displayed a distinct peak at *x* = 130 nm, which implied an increased electrocatalytic activity of the NiO edge.

**Figure 13:**
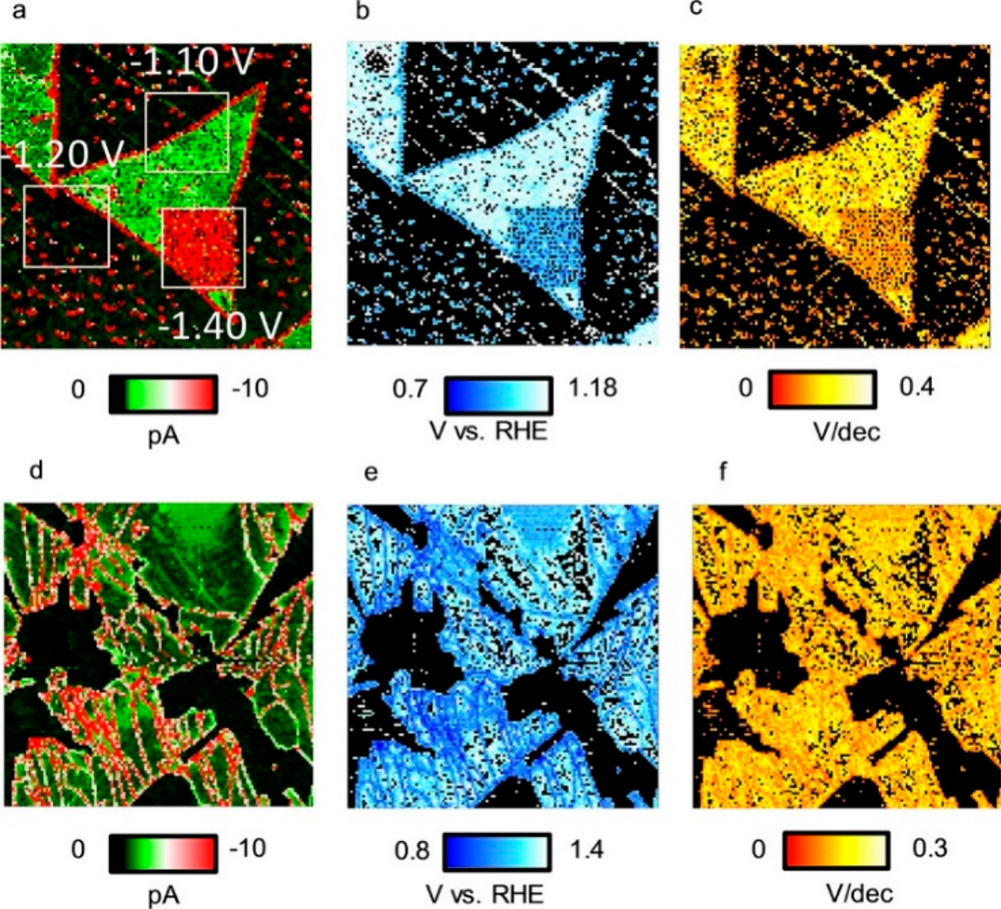
(a) Current response imaging of MoS_2_ nanosheet supported on HOPG. Squares marks regions where the scan reached the correspond voltage. (b) Overpotential imaging. (c) Tafel slope imaging. (d) Current response imaging of overannealed Mo_2_S nanosheet. (e) Overpotential imaging (f) Tafel slope imaging. Scan size was 10 × 10 μm^2^. Adapted with permission from ref (106). Copyright 2019 Wiley-VCH.

In a similar way, electrochemical mapping was utilized for mapping the electrocatalytic hydrogen evolution reaction (HER) on MoS_2_ nanosheets. Site-dependent HER yield on MoS_2_ nanosheets was probed by conducting high spatial resolution SECCM measurements.^106^ Cyclic voltammetry measurements were taken from different regions of the catalytic system to enable the imaging of MoS_2_ nanosheets on HOPG support, showing the current response (Figure 13a), the overpotential (Figure 13b), and the Tafel slope (Figure 13c). Enhanced HER activity, indicated by high current response and low overpotential and Tafel slope, was observed at the edges of the nanosheets, or when the scan reached −1.40 V vs reversible hydrogen electrode (RHE), the threshold voltage for enhanced HER activity. Additional information about site-dependent reactivity was obtained following annealing of the nanosheets, leading to high density of cracks within the sheets, as visualized by the SECCM imaging (Figure 13d–f). Crack regions showed similar behavior to that of the edges, thus further demonstrating the connection between defects and superior HER activity.

**Figure 14:**
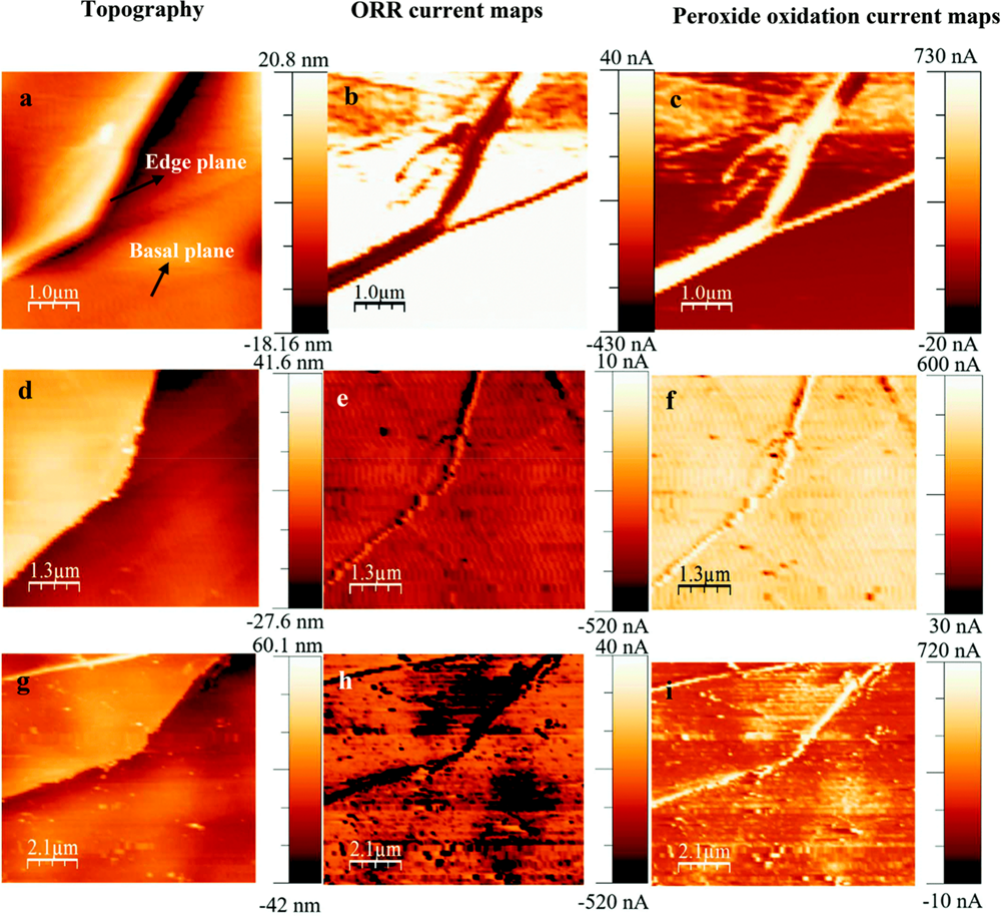
(a) Topography image of HOPG. (b) ORR current map of the same area. (c) Peroxide oxidation current map of the same area. (d–f) After ammonia plasma treatment. (g–i) After exposure to FeCl_3_. Adapted with permission from ref (108). Copyright 2021 MDPI.

Structural and compositional differences between sites can lead to site-dependent electrocatalytic reactivity, as recently demonstrated for oxygen reduction reaction (ORR) on Fe–N–HOPG catalyst.^107,108^ A unique setup combining atomic force microscopy and scanning electrochemical microscopy (AFM-SECM) was used to attain high spatial resolution reactivity and topography analysis. To study the compositional effect of the catalyst surface, the catalytic activity of Fe–N–HOPG was investigated at three different stages. First, the pure HOPG was scanned, showcasing the topography image of the basal and edge plane (Figure 14a). Current mapping of oxygen reduction and peroxide oxidation (Figure 14b,c, respectively) clearly suggests that the ORR activity is mainly located at the edge plane. The structure-dependent reactivity was explained by the formation of sp^3^ carbons at the catalyst edge plane. N-HOPG sites were prepared by treating the surface with ammonia plasma. The topography and current images revealed that the site-dependent reactivity has nearly vanished following this treatment, and in addition, the current values significantly deteriorated (Figure 14d–f). N-HOPG was then immersed in FeCl_3_ solution to functionalize the surface with Fe sites. Topography, ORR current, and peroxide oxidation current images shown in Figure 14g–i. These measurements revealed enhanced currents and superior activity at the edge planes, as well as some defective sites at the basal plane. The defect sites at the edge and basal planes were presumed to be hosts for Fe particles and to form sites of ferrous ions attached to pyridinic N atoms, giving rise to enhanced ORR activity. Overall, high spatial resolution AFM-SECM results show that composition variations are induced by local defect sites and modifies the electrocatalytic activity.

**Figure 15:**
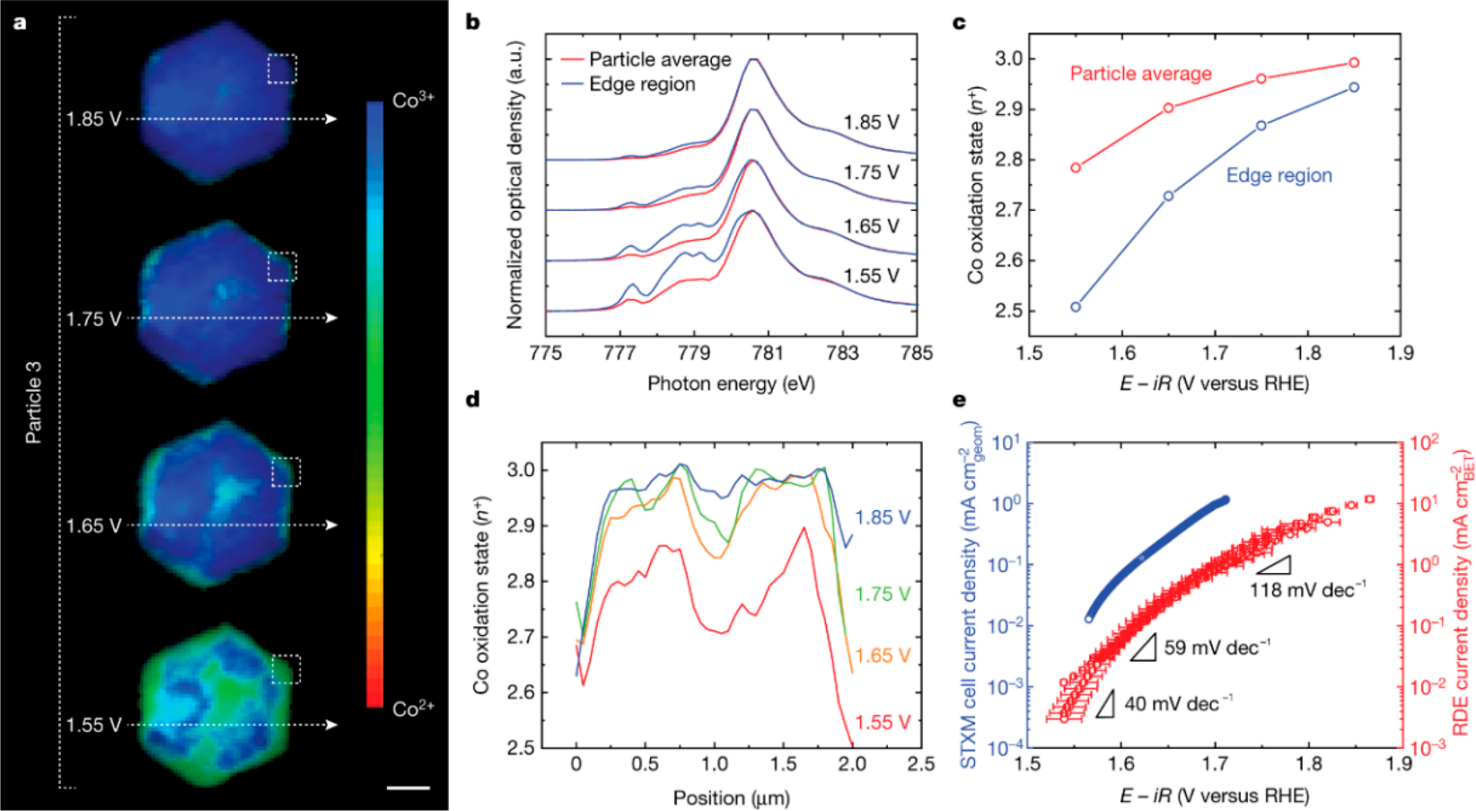
(a) Oxidation state mapping of a single β-Co(OH)_2_ particle at the applied voltages. Scale bar is 500 nm. (b) STXM-XAS spectra of the entire particle and edge region (marked by dashed square on a) at the applied voltages. (c) Plot of the corresponding average oxidation state at the edge and particle average as a function of voltage applied. (d) Oxidation state profiling along the horizontal axis marked by white arrow in a. (e) OER Tafel data obtained in STXM cell (blue) and RDE cell (red). Adapted with permission from ref (112). Copyright 2022 Nature Publishing Group.

In addition to structural variations, the electrocatalytic reactivity can be influenced by local differences in the oxidation state of the electrocatalysts. Thus, nanoscale analysis of the connection between oxidation state and electrocatalytic reactivity is of high importance because electrocatalytic properties are often linked to the oxidation state of the transition metal catalyst, as shown for metal(oxy)hydroxides in oxygen evolution reaction (OER).^109,110^ Subparticle scanning transmission X-ray microscopy (STXM) measurements demonstrated variations in the oxidation state of β-Co(OH)_2_ catalyst.^111,112^ Oxidation state mapping images of a single particle were obtained at different voltages (Figure 15a). Co^3+^ species, associated with β-CoOOH, were obtained at 1.55 V vs RHE, mainly on the basal plane. Interestingly, more regions exhibit Co^3+^ oxidation state as the potential increased and at 1.85 V the particle was fully oxidized. STXM-X-ray absorption spectroscopy (XAS) measurements from the edge sites provided a comparison between the oxidation state of the edge and the averaged signal across the particle. This analysis further confirmed that the particle and its edge are oxidized at 1.85 V (Figure 15b,c). In addition, high spatial resolution profiling of the oxidation state along the horizontal axis of the particle indicated a similar oxidation pattern (Figure 15d). The connection between oxidation state and oxygen evolution reaction yield was shown by comparing the Tafel data collected from the STXM cell and rotating disc electrode cell (blue and red curves in Figure 15e, respectively). The increase of the Tafel slope and the corresponding behavior of these cells implied that Co^3+^ is the active specie for OER. Although ensemble-based measurements displayed some Co^3+^ species at certain voltages below 1.85 V, STXM information made it possible to probe the exact location and nature of active sites.

Electrocatalytic reactivity mapping, conducted by SECM and SECCM measurements, demonstrated the impact of defect sites, such as cracks and interfaces. In addition, the integration of electrochemical mapping with topography or spectroscopy analysis uncovered the dynamic structural and electronic variations that are induced under electrocatalytic reaction conditions and their influence on the resulting reactivity. Such *operando* measurements provide unique understanding about the active sites for electrocatalysis, which cannot be gained by ensemble-based measurements.

### 3.2 Site-Dependent Selectivity

**Figure 16:**
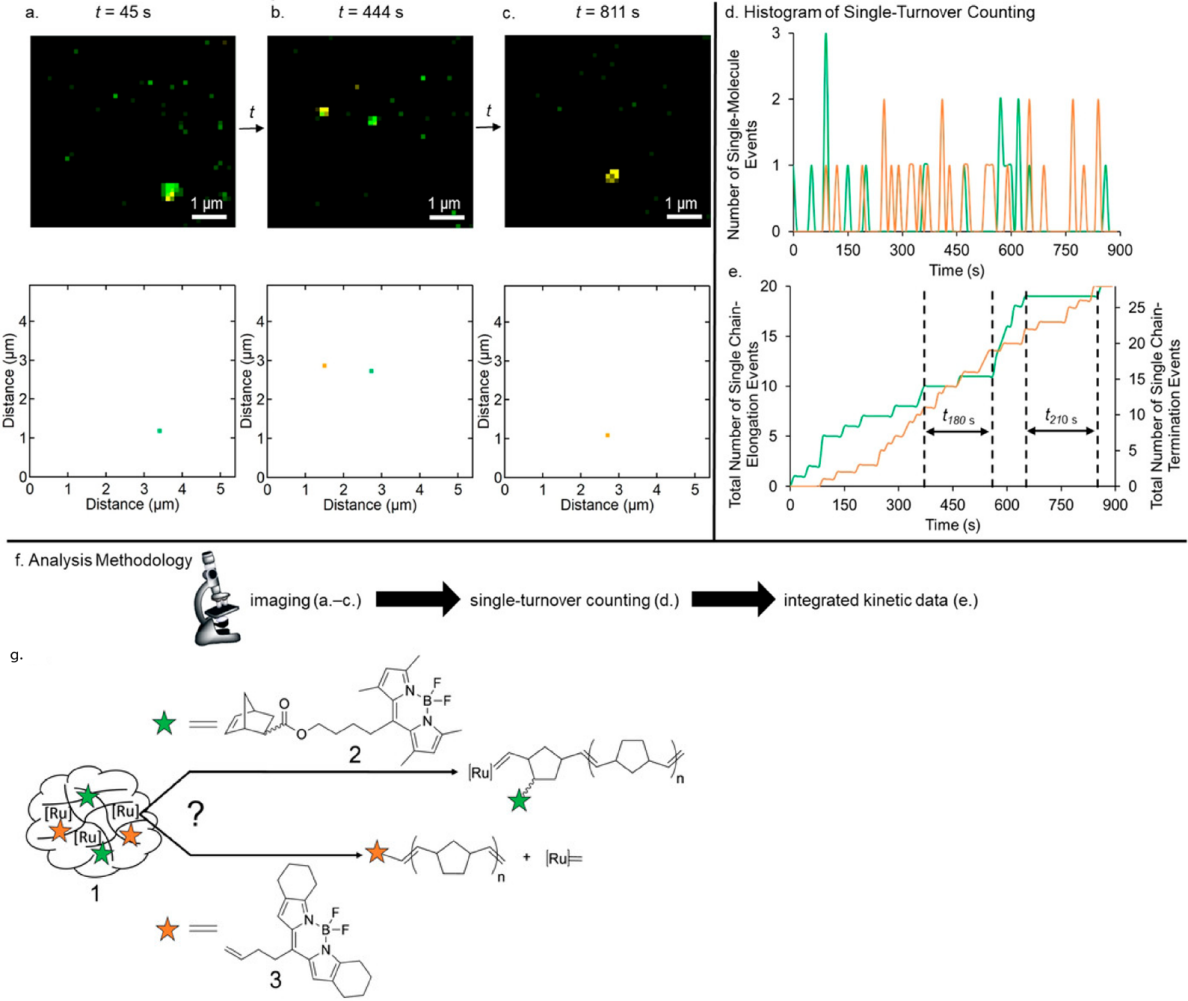
(a–c) Comparison of diffraction limited images (top) and super-resolved images (bottom) for single frames at *t* = 45, 444, 811 s, respectively. (d) Histogram data of single chain-elongation (green) and -termination (orange) events as of function of time. (e) Kinetic time traces comparing chain-elongation (green) and termination (orange). (f) Imaging and analysis methodology. (g) Schematic illustration of the two possible reaction pathways and their fluorescent marker. Adapted with permission from ref (17). Copyright 2019 Wiley-VCH.

High spatial resolution spectroscopy is not limited to the general analysis of surface reactivity, it could also be used to gain mechanistic understanding on reaction selectivity and thus provide crucial insights about the reaction mechanism on different surface sites. For instance, the fundamental question of how a single molecular catalyst selectivity is altered with time and space was recently addressed by probing the selectivity of molecular ruthenium catalyst’s toward different polymerization pathways.^17^ To detect selectivity based on a single turnover event, norbornene was tagged by two different fluorescent probes; one enables the detection of chain elongation event, and the second a chain termination event (Figure 16g). One second snapshots of catalyst selectivity toward either single-chain elongation or single-chain termination were acquired.

Parts a–c of Figure 16 depict one second super-resolution fluorescence microscopy imaging of a single aggregate. Notably, each of the reaction pathways was assigned to a specific color: green for chain elongation and orange for chain termination. The selectivity pattern was temporally resolved by counting the events corresponding to each reaction pathways within the one second time frame. Figure 16d shows the histogram of the elongation and termination events with respect to time. The summation of events over time for each of the reaction pathways, as shown in Figure 16e, provides informative kinetic behavior which reveals the variation in selectivity with time. This variation is attributed to the dynamic local environment of each molecular catalyst within a single aggregate, such as variation in solvation, segment movement, and chain growth that can impact the reactivity pattern.

**Figure 17:**
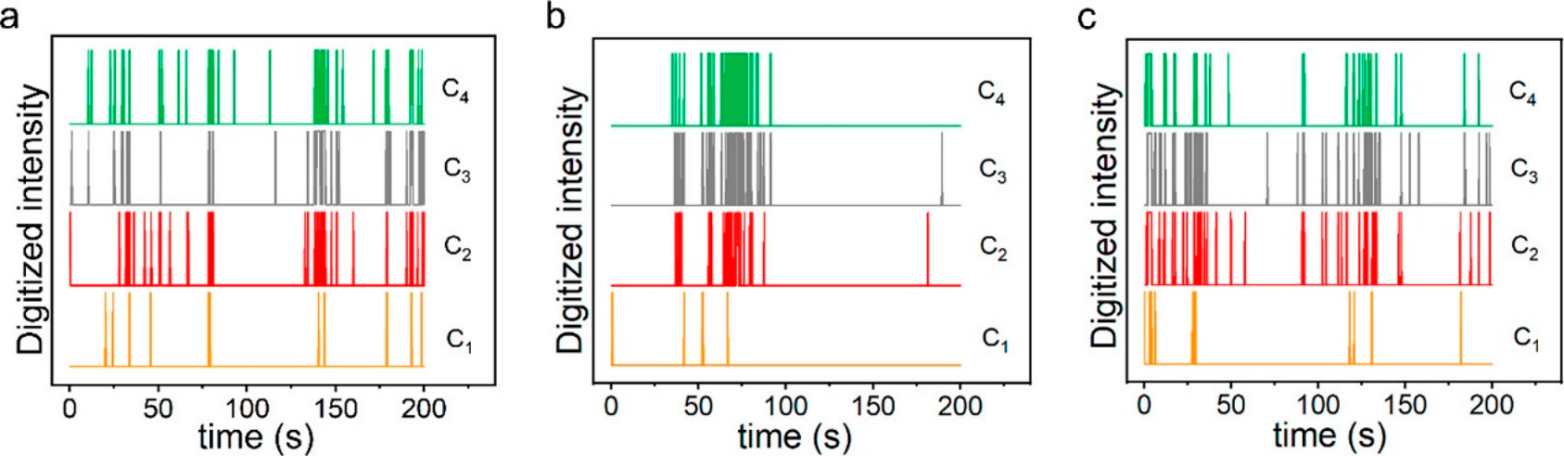
C_1_, C_2_, C_3_, and C_4_ species stochastic trajectories showing appearance of the various products on three different Ag NP (a–c) over a period of 200 s. Adapted with permission from ref (113). Copyright 2021 American Chemical Society.

Observed differences in reactivity and selectivity of catalytic systems were correlated to morphological, structural, and compositional heterogeneities. However, when these factors are ruled out, spatial and temporal heterogenic behavior of a catalytic system may still take place. Plasmon-excitation driven CO_2_ reduction reaction was explored on single Ag NPs using surface enhanced Raman scattering (SERS) spectroscopy.^113^ The products of the reaction were classified into four groups according to the number of carbon atoms in the product molecule (C*_n_*, when *n* = 1, 2, 3, 4). Figure 17 shows the product formation on three different particles over time. These trajectories enabled the examination of product selectivity for each NP scatterer at different time segments. Significant differences in selectivity were found between each NP. Furthermore, changes in selectivity were spotted overtime on each NP, excluding the possibility that the selectivity is determined solely by structure and morphology. Such spatial and temporal heterogeneity in selectivity might be explained by stochastic noise that arises from thermal fluctuations. The latter serves as an important insight for considering the randomness of events in addition to the static properties of catalysts.

**Figure 18:**
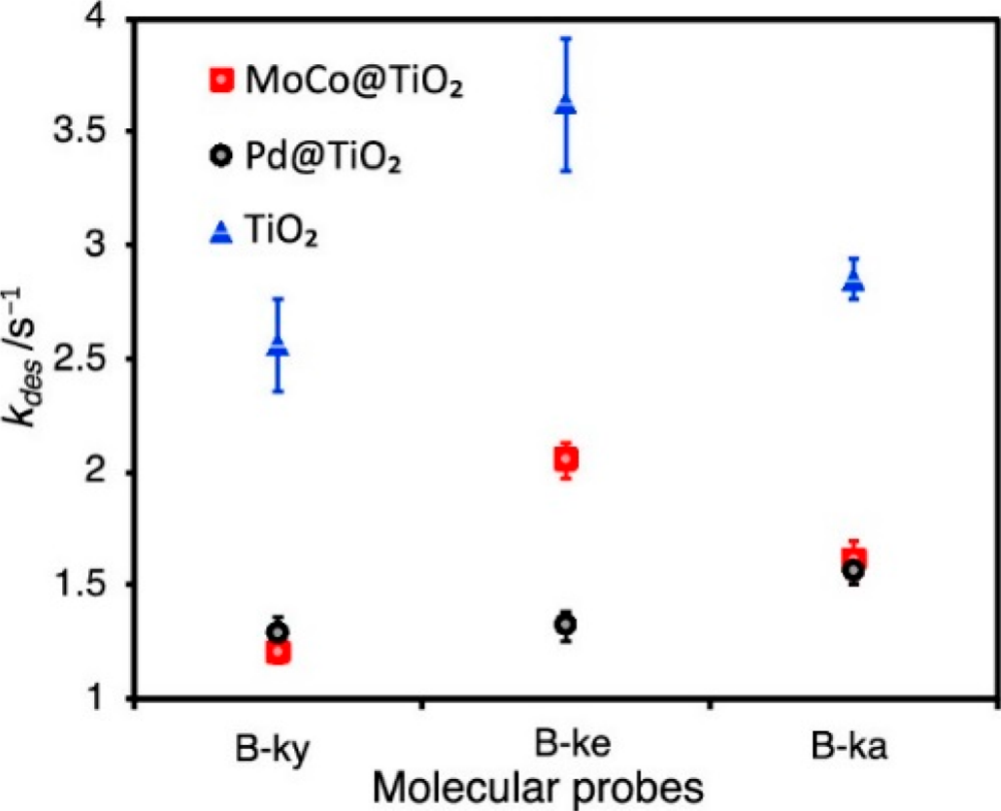
Desorption rates, *k*_des_, for various catalysts and molecules. B-ky is alkyne, B-ke is alkene, and B-ka is alkane. MoCo@TiO_2_, Pd@TiO_2_, and bare TiO_2_ are represented by red squares, black dots, and blue triangles, respectively. Adapted with permission from ref (116). Copyright 2021 American Chemical Society.

Mechanistic insights on the selectivity in photocatalytic alkynes semihydrogenation reaction^114^ and the influence of product desorption rate were analyzed by fluorescence microscopy. Interestingly, when using MeOH as the hydrogen source, MoCo@TiO_2_ demonstrated higher selectivity toward alkene formation than that of Pd@TiO_2,_^115^ and product desorption rate was identified as a possible factor influencing the resulting selectivity.^116^ To validate the impact of product desorption rate on selectivity, boron dipyrromethene (BODIPY) molecules containing alkyne, alkene, and alkane side groups were synthesized and served as probes for total internal reflection fluorescence (TIRF) microscopy. In this experimental setup, each adsorption of a BODIPY molecule to the surface yields a fluorescence signal. Because the measured burst time is equivalent to the desorption time, it enabled calculatation of the different desorption rates *k*_des_. Three catalysts were compared, namely Pd@TiO_2_, MoCo@TiO_2_, and bare TiO_2_, and each system was exposed to 1 nM solution of the different BODIPY molecules. The measured desorption rates of Pd@TiO_2_ were low and exhibited no significant difference between alkyne, alkene and alkane, indicating that the alkenes and alkynes remain adsorbed for relatively long period of time (Figure 18). Contrastingly, bare TiO_2_ and MoCo@TiO_2_ displayed higher *k*_des_ for the alkene BODIPY. The higher desorption rate of alkene may suggest that this is the main driving force for selective alkene formation.

**Scheme 3:**
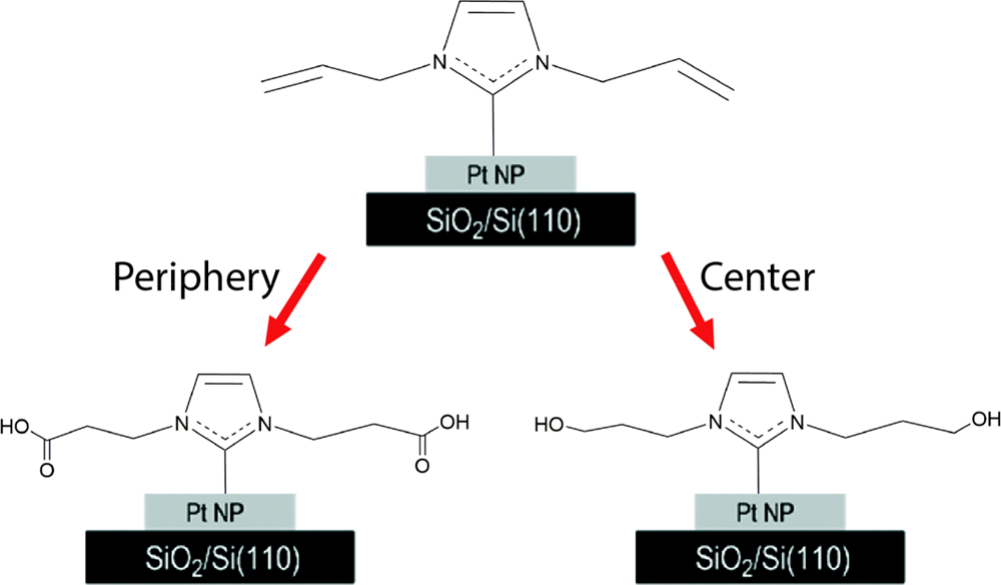
Illustration of the Two Oxidation Reaction Pathways Probed by IR Nanospectroscopy Measurements

Nanoscale selectivity analysis of catalytic reactions can lead to valuable mechanistic insights. However, the detection of intermediates is highly challenging due to their low surface concentration and potentially short lifetime. Allyl functionalized NHCs enabled to measure site-dependent selectivity reactions^82^ due to their potential to partially oxidize to a hydroxyl or fully oxidize to a carboxylic acid (Scheme 3). Exposure to mild oxidizing conditions yielded site-dependent selectivity pattern, in which variations in IR nanospectroscopy measurements implied that a partial oxidation occurred at the particle’s center and a full oxidation was accomplished at the edges of the particle. Exposure to harsher oxidizing conditions yielded additional vibrational peaks at both the center and edge of the particle, demonstrating the less selective and more destructive nature of harsher oxidizing conditions.

It should be noted that multiproduct detection requires the development of probe molecules that will enable identification of the formation of specific products. This was achieved by synthesis of two fluorescence reactants or by using surface anchored molecules with chemically active groups that yield thermally stable intermediates. By using these specifically synthesized probe molecules, high spatial resolution selectivity measurements were conducted and provided insights about selectivity mechanism and the ways by which local variations in the catalyst and its environment can impact the resulting products distribution.

### 3.3 Facet-Dependent Reactivity

Dissimilar crystallographic facets often induce altered catalytic reactivity which rise from the specific atomic order on the catalyst surface.^117,118^ Therefore, facet-controlled nanocrystals have been widely employed for analyzing the reactivity of different crystallographic facets. However, the knowledge gained by ensemble-based measurements is limited due to interparticle variations, which rise from synthetic limitations, and the natural inhomogeneity at the single nanocrystal level that includes facet interfaces and defects. High spatial resolution measurements can therefore improve our understanding of facet dependent reactivity by probing the reactivity of single nanocrystals to identify variations and correlations in the reactivity pattern and uncover the catalytic impact of neighboring facets, defects, and interfaces.

#### 3.3.1 Thermal Catalysis

**Figure 19:**
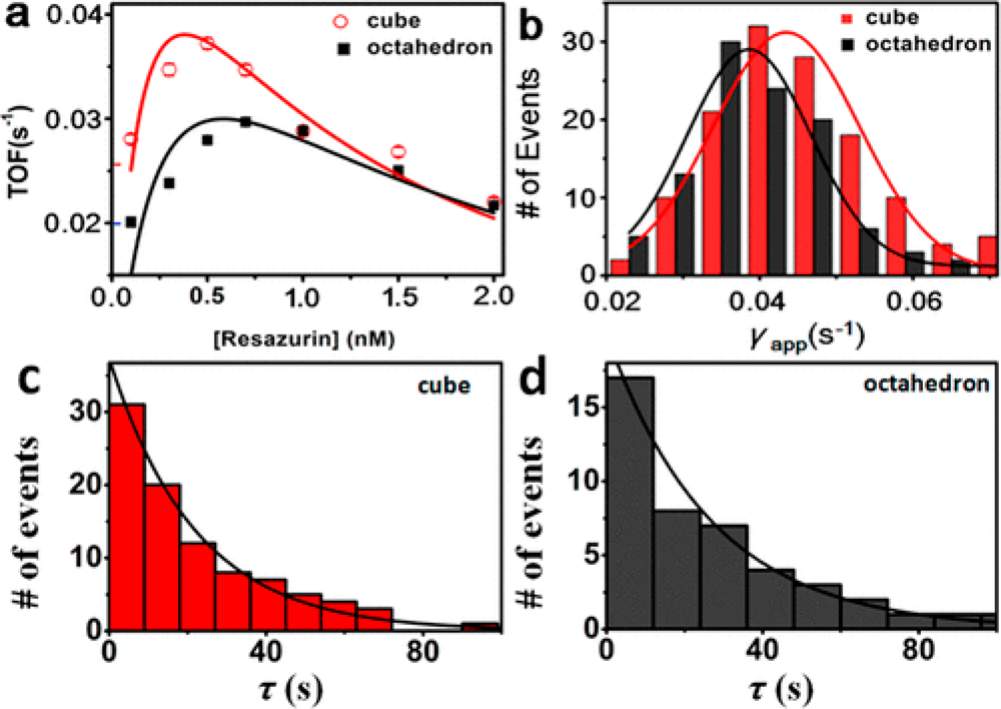
(a) Turnover frequencies at different reactant concentrations that were measured by integration of single particle reactivity analysis of single nanocubes (red) and single nano-octahedrons (black). (b) Distribution of the effective rate constant of single nanocubes (red) and nano-octahedrons (black). Distribution of turnover time for single nanocube and nano-octahedron are shown in c and d, respectively. Adapted with permission from ref (119). Copyright 2017 American Chemical Society.

The facet-dependent catalytic activity of Pd nanocubes and nano-octahedrons bearing (100) and (111) facets, respectively, were examined to determine the catalytic activity of each facet.^119^ Resazurin reduction to the fluorescence active resorufin served as a marker for catalytic turnover. By measuring the fluorescence intensity versus time, it was possible to observe fluorescence intensity with time intervals τ, representing the time for a single turnover, which enabled the calculation of turnover frequencies (TOF) of each particle. Comparing the TOFs versus resazurin concentration for the nanocubes and nano-octahedrons revealed that the TOF increase with concentration was faster for the nanocubes (Figure 19). The latter also reached a higher maximal TOF, indicating the enhanced reactivity of (100) facets. This increase was followed by a decay for both catalysts at high concentrations due to blocking of the active sites by resazurin. This study demonstrated, based on single particle measurements, the superior reactivity of (100) over (111) facets in Pd nanocrystals. However, it should be noted that the fluorescence signal was obtained from the nanoparticle as a whole, therefore could not be directly assigned to specific subparticle locations.

**Figure 20:**
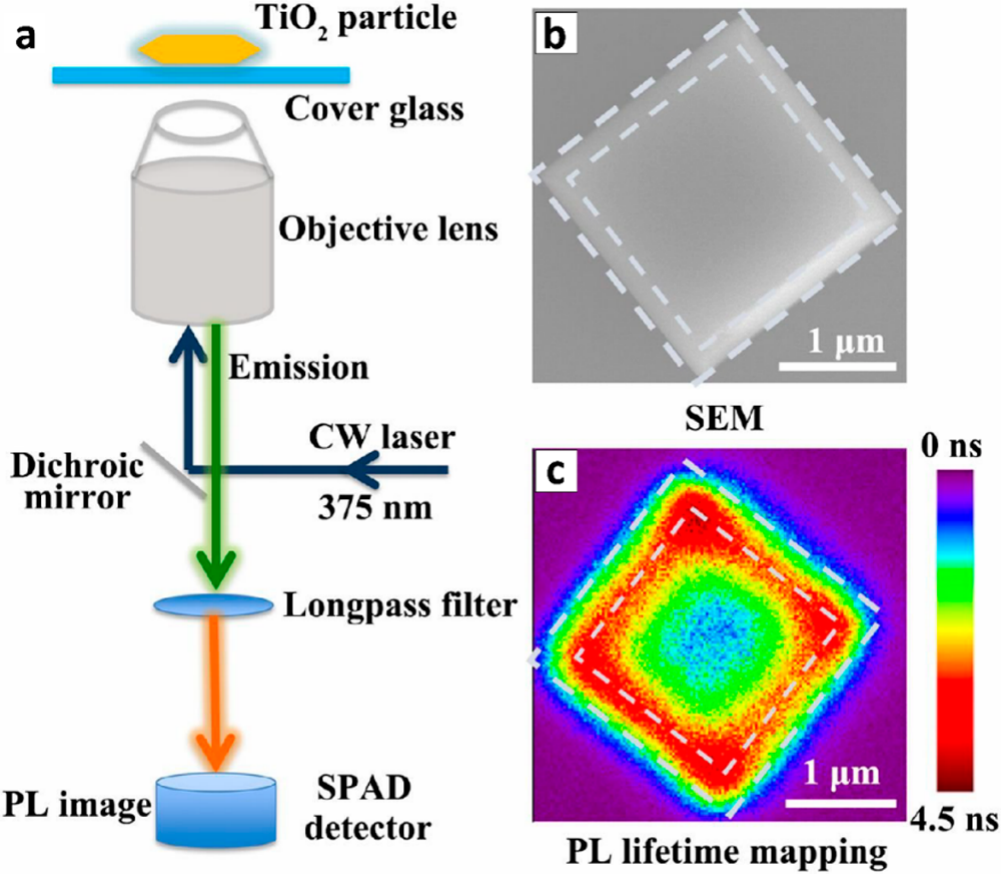
(a) Schematic illustration of the setup for single molecule PL measurements. (b) SEM image of single anatase TiO_2_. White dashed lines marks the (101) facet borders. (c) 2D mapping of PL lifetimes on the same TiO_2_ particle. Adapted with permission from ref (120). Copyright 2019 Proceedings of the National Academy of Sciences.

Dissimilar crystallographic facets also influence the electronic properties and thus can impact the photocatalytic reactivity distribution, as demonstrated in single particle photoluminescence (PL) measurements that uncovered facet-dependent reactivity in TiO_2_ photocatalyst.^120^ Photocatalytic activity is known to be highly dependent on charge carrier lifetimes, and longer PL lifetime is expected to enhance the catalytic activity at a specific surface site. Single particle photoluminescence (PL) measurements were performed on anatase TiO_2_ particle that is constructed of a (001) facet at its top and (101) facets at its side. Figure 20b marks the border of these facets by dashed lines. PL lifetime measurements revealed facet-dependent PL lifetimes (Figure 20c). Facets centers exhibited relatively short lifetimes, whereas slightly increased lifetimes were observed at the edges in between the (001) and (101) facets.

Notably, the longest lifetimes were observed at the corners located at the junction of (101) and (001) facets. Reduction and oxidation reactions were further investigated by measurements of fluorescent resorufin formation from amplex red and resazurin, in which amplex red and resazurin were used as probes for photogenerated holes and electrons, respectively. Both reduction and oxidation reactions exhibited enhanced catalytic activity at the edges and corners of the crystal, consistent with the PL lifetime mapping. Based on DFT calculations, it was suggested that when electron–hole pairs are photogenerated, electrons flow from (001) facet to (101) facet through the heterojunctions of the facets at the edges and corners, while the holes flow in the opposite direction. Electrons and holes are then trapped and create well separated electron and holes pairs, therefore achieving longer PL lifetime at these locations, which supply charge carriers for oxidation and reduction reactions.

**Figure 21:**
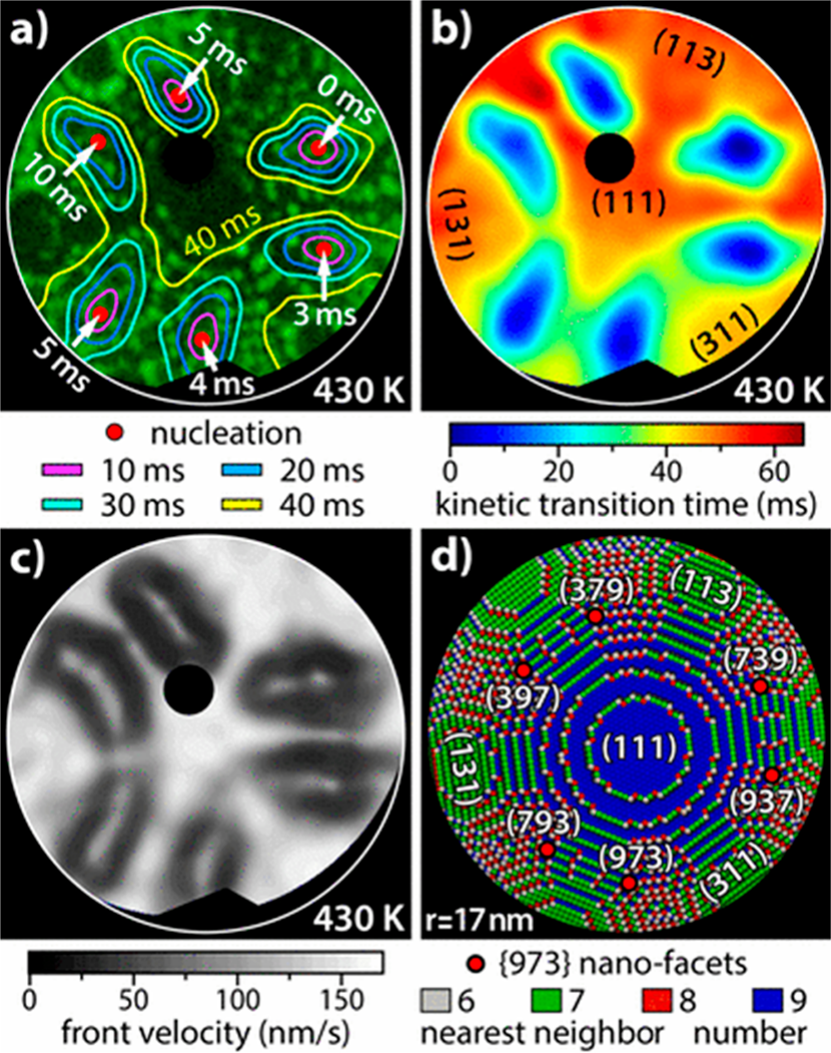
(a) Map of oscillating reaction fronts imposed on FIM image of the tip. Nucleation occurs in the position of red dots at the indicated time, and the lines show the front at times with 10 ms intervals. (b) Map of time-scan of fronts over the first 65 ms of the reaction. (c) Map of fronts velocities. (d) Model representing the corrugation by the coordination number at each position. Adapted with permission ref (121). Copyright 2021 American Chemical Society.

The influence of different crystallographic facets on catalytic reactivity is not necessarily limited to a specific facet and communication between neighboring facets can lead to increased catalytic activity as demonstrated for H_2_ oxidation on Rh nanoparticle.^121^ This reaction was modeled by a nanometric Rh tip mimicking a round nanoparticle. The tip was characterized at the atomic level by field ion microscopy (FIM), and in combination with field emission microscopy (FEM), the oscillating H_2_ oxidation reaction pattern was measured. The FIM-FEM setup allowed spatial and temporal observation of oscillations of H_2_ oxidation on the tip by applying automated procedure called transition point-tracking (TPT) in which the intensity of each pixel is calculated with high temporal resolution. The authors used this ability to reveal specific atoms with (973) atomic configurations that were located in between relatively flat facets and identified these sites as initiators for kinetic transitions (Figure 21).

The location of the most reactive sites between adjacent flat facets indicated that there is an interfacet effect, where the flat facet provides hydrogen supply to the (973) nanofacets, allowing the initiation of reaction fronts. Figure 21a shows the propagation of reaction front. The reaction is initiated at the (973) nanofacets, and then the fronts grow at a certain velocity (Figure 21b,c). A model of the tip surface, with coordination number and facet locations, is shown in Figure 21d, where the fronts with the highest velocities are those who correlates to low corrugation.

#### 3.3.2 Photoinduced Catalysis

**Figure 22:**
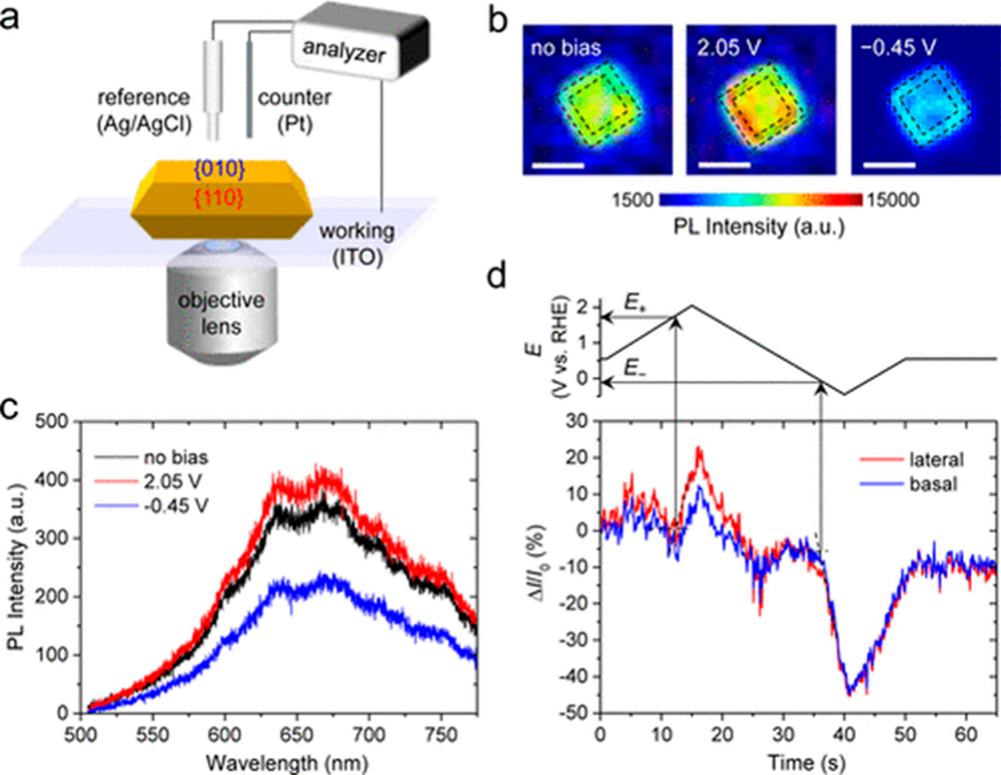
(a) Illustration of the spectroelectrochemical setup for single particle measurements. (b) PL images of a single BiVO_4_ particle without applied potential and while applying potentials of 2.05 and −0.45 V vs RHE. Black dashed lines mark the border between lateral and basal facet and the border of the particle edge. Scale bars are 1 μm. (c) PL spectrum under various potentials. (d) Plot of the relative change in PL intensity with the potential scan. Adapted with permission from ref (122). Copyright 2016 American Chemical Society.

Single particle photoluminescence (PL) microscopy was harnessed for mapping the charge carrier distribution in faceted BiVO_4_ photocatalyst and its connection with the basal (010) and lateral (110) facets.^122^ The localized variations in the PL signal were attributed to active sites with higher probability to trap electrically and photogenerated holes in trapped states above the valence band of the semiconductor. Spectroelectrochemistry measurements were conducted to explore the radiative recombination (Figure 22a).

PL images of single particles under different potentials of 2.05 and −0.45 V vs RHE and with no applied potential showed inhomogeneous PL intensities within the particle. The highest PL intensity was recorded for the positively applied potential and was significantly reduced when negative potential was applied (Figure 22b,c). Notably, plotting the PL intensity changes as a function of time for the lateral (110) and basal (010) facets show positive and negative peaks, when the potential gradually shifts to 2.05 V and −0.45 V vs RHE, respectively (Figure 22d). For a positive potential, the lateral (110) facets exhibit an intense peak, indicating that the oxidation is easily facilitated at this facet. Moreover, it implies that hole sites are mostly located at this facet, whereas electrons are spread over the entire particle. These results demonstrate the ability to gain insights critical for catalysis by conducting high spatial resolution PL measurements under reaction conditions without having to measure directly the reactivity.

**Figure 23:**
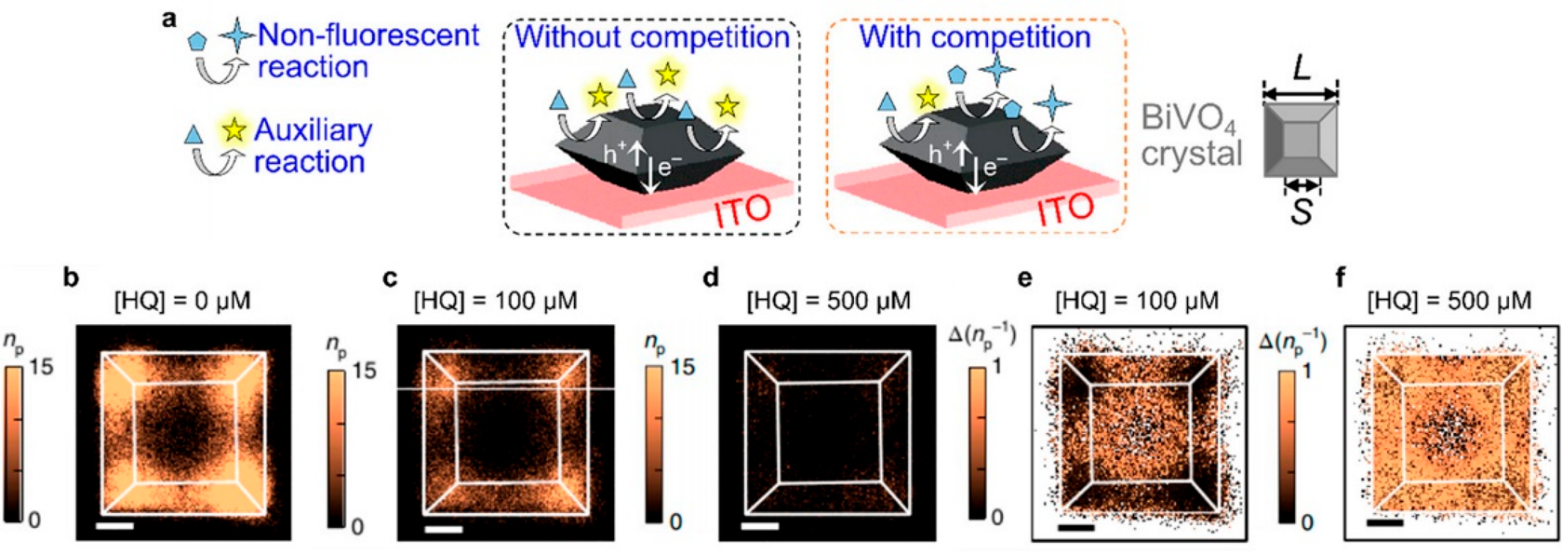
(a) Experimental setup for competitive imaging of nonfluorescent reaction. (b) 2D histogram image of number of resorufin product (*n*_p_) over a single BiVO_4_ particle after 22.5 min reaction and without presence of HQ. (c) 2D histograms of *n*_p_ with competition of 100 μM and (d) 500 μM HQ concentration. (e) 2D histograms of the difference of Δ*n*_p_^–1^ between (b) and (c,d). All scale bars are 500 nm. Adapted with permission from ref (124). Copyright 2020 American Chemical Society.

As demonstrated in the previous examples, studying catalytic reactivity by high spatial resolution fluorescence microscopy is inherently limited due to the requirement for highly fluorescent products. A possible route to overcome this limitation was demonstrated by using competitive reactions that enabled to probe the photoelectrocatalyzed reduction of hydroquinone (HQ) to benzoquinone on semiconducting BiVO_4_ catalyst containing basal (010) and lateral (110) facets.^123,124^ The experimental approach to visualize the nonfluorescent reaction was based on competition with fluorescent reaction, the reduction of amplex red (AR) into resorufin (Figure 23a). Monitoring of the nonfluorescent reaction was achieved by comparing the difference between the fluorescence image of AR reduction without competition (Figure 23b) with those obtained with competition (Figure 23c,d).

Additional comparisons demonstrated the difference in number of fluorescent product (Δ*n*_p_) with and without competition, indicating the facet-dependent reactivity. Moreover, the difference in the inverse number of resorufin products (Figure 23e,f) was proportional to the rate constant of HQ reduction (*K*_HQ_) for a given concentration of AR, demonstrating that HQ was adsorbed on specific surface sites within the particle. These results specify that there is a stronger adsorption of HQ on basal (010) than lateral (110) facets. Furthermore, within the lateral facets, adsorption was found to be stronger in the center of the facet compared to corners between the two lateral facets. The competitive method presented in this study unlocks the possibility to explore nonfluorescent reactions via fluorescence microscopy. However, it should be noted that this approach is currently limited to catalysts that can be sufficiently reactive to compete with a fluorescent reaction over all areas of interest within a catalytic particle and that this method cannot differentiate poisoning and reactivity of nonfluorescent reactions because both can locally block the fluorescent signal.

#### 3.3.3 Electrocatalysis

**Figure 24:**
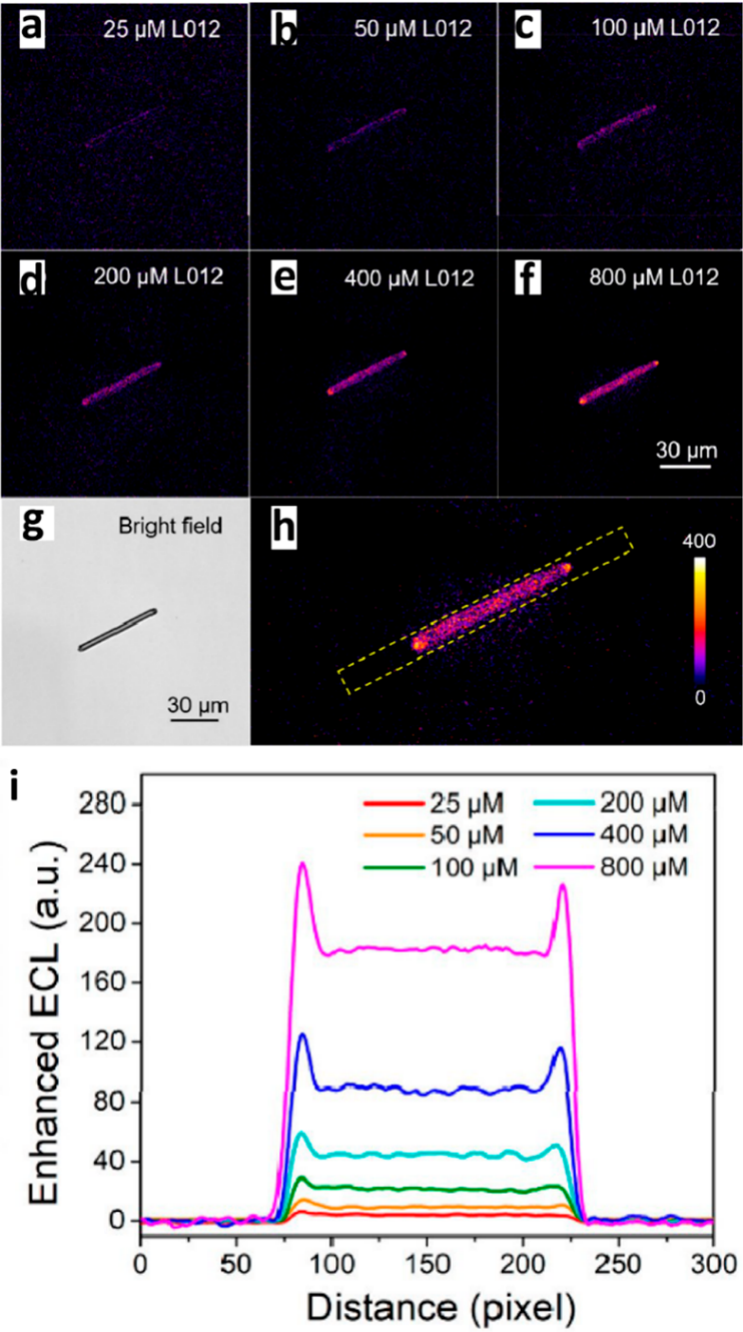
(a–f) Electrochemiluminescence signals obtained from a single ZnO nanorod at various L-012 concentration. (g) Bright field image of a single ZnO nanorod. (h) Higher magnitude of the image shown in F. (i) Electrochemiluminescence signals along the rod axis at various concentrations. Adapted with permission from ref (125). Copyright 2019 American Chemical Society.

Facet-dependent electrocatalytic reactivity of ZnO nanorod was mapped by probing the electro-oxidation of L-012 molecule (luminol analogue, 8-amino-5-chloro-7-phenylpyrido[3,4-*d*]pyridazine-1,4(2*H*,3*H*)dione) to endoperoxide, which forms a fluorescent active aminophthalic acid molecule.^125^ The reaction was catalyzed by ZnO nanorod with (100) facets along the rod and (002) facets at the tip. Electrochemiluminescence signal from a single nanorod was collected at different L-012 concentrations (Figure 24). 2D histogram of the electrochemiluminescence signals revealed differences in reactivity between the tips and sides of the rods. The high luminescence signal appeared to extend beyond the (002) facet, due to diffusion of L-012 radicals. Facet-dependent electrocatalytic reactivity was correlated with higher adsorption affinity of O_2_ on (002) over (100) facet. Because O_2_ reacts with L-012 radical to generate endoperoxide, the (002) facets are expected to show enhanced reactivity. The observed extension of reactivity beyond the (002) facet emphasized the importance of measuring different surface structures with multiple types of surface sites. Not only that the (002) facet was shown to be more active, but it also induced higher reactivity on a less reactive facet.

**Figure 25:**
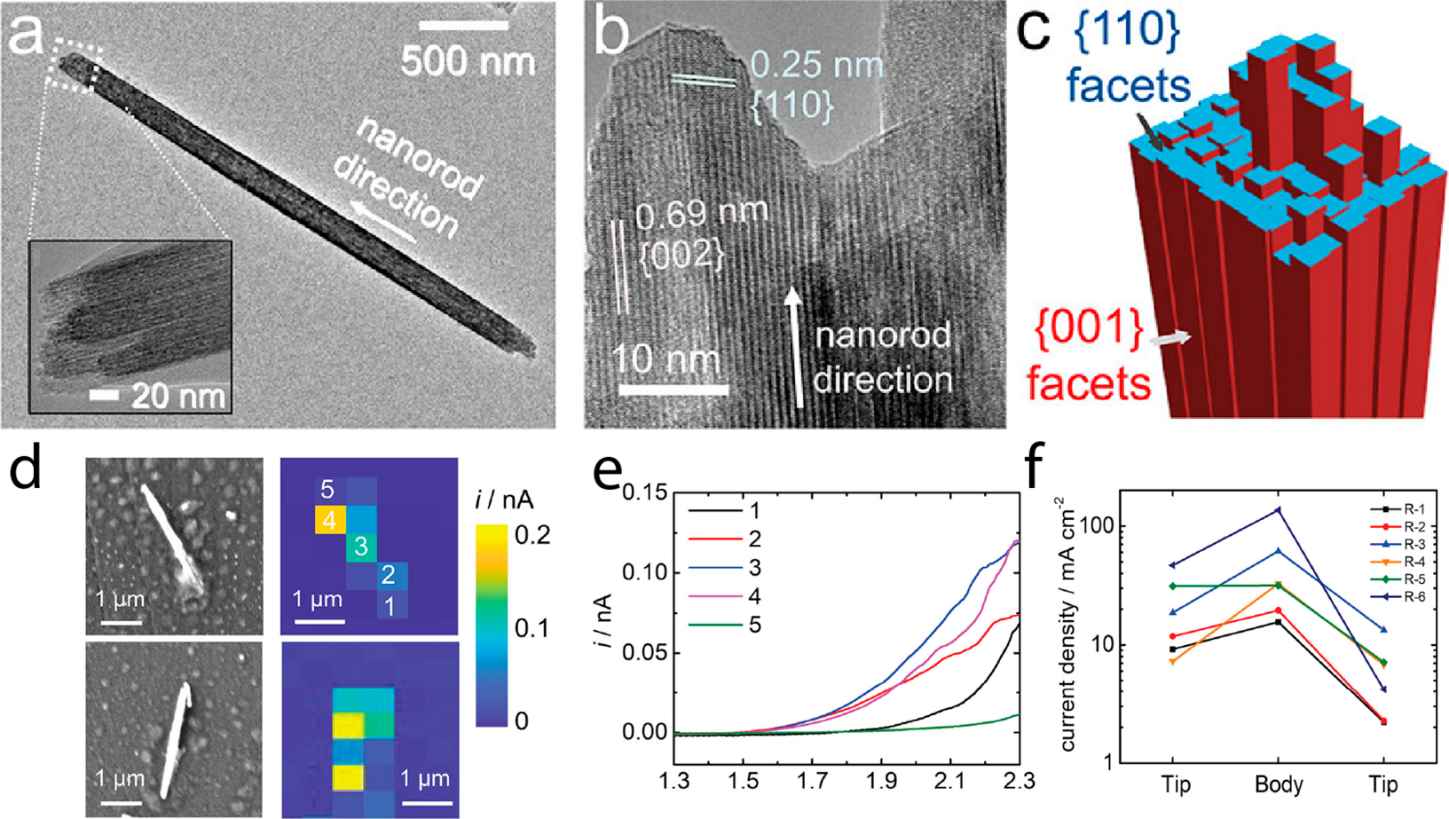
(a) TEM image of hematite nanorod. Inset: zoom-in of the tip section. (b) HRTEM image of the tip. (c) Schematic showing the facets exposed at the tip vs the body. (d) SEM images of hematite nanorods (left) and their colocalized high-resolution SECCM current map at 2.3 V (right). (e) Representative local voltammograms at different positions on the same hematite nanorods as indicated in (d). (f) Summary of OER current density at 2.3 V at the tip and the body of six different nanorods. Adapted with permission from ref (126). Copyright 2022 American Chemical Society.

Facet-dependent reactivity correlations between and within electrocatalysts were probed in hematite (α-Fe_2_O_3_) nanorods by SECCM measurements combined with colocalized structural characterization.^126^ SECCM measurements revealed an heterogeneous electrocatalytic OER yield at different regions within individual nanorods (Figure 25). Lower current and current density were probed around the nanorod’s tip. The heterogeneity was correlated to structural variations because the tip that is mainly composed of (110) facets while the nanorod’s body is constructed of (100) facets. Interestingly, this reactivity pattern indicates that in this system, the facets with lower surface atoms density are less reactive. These results imply the benefit of synthesizing longer hematite nanorods, and hematite nanostructures exposing more active facets for enhanced OER activity.

**Figure 26:**
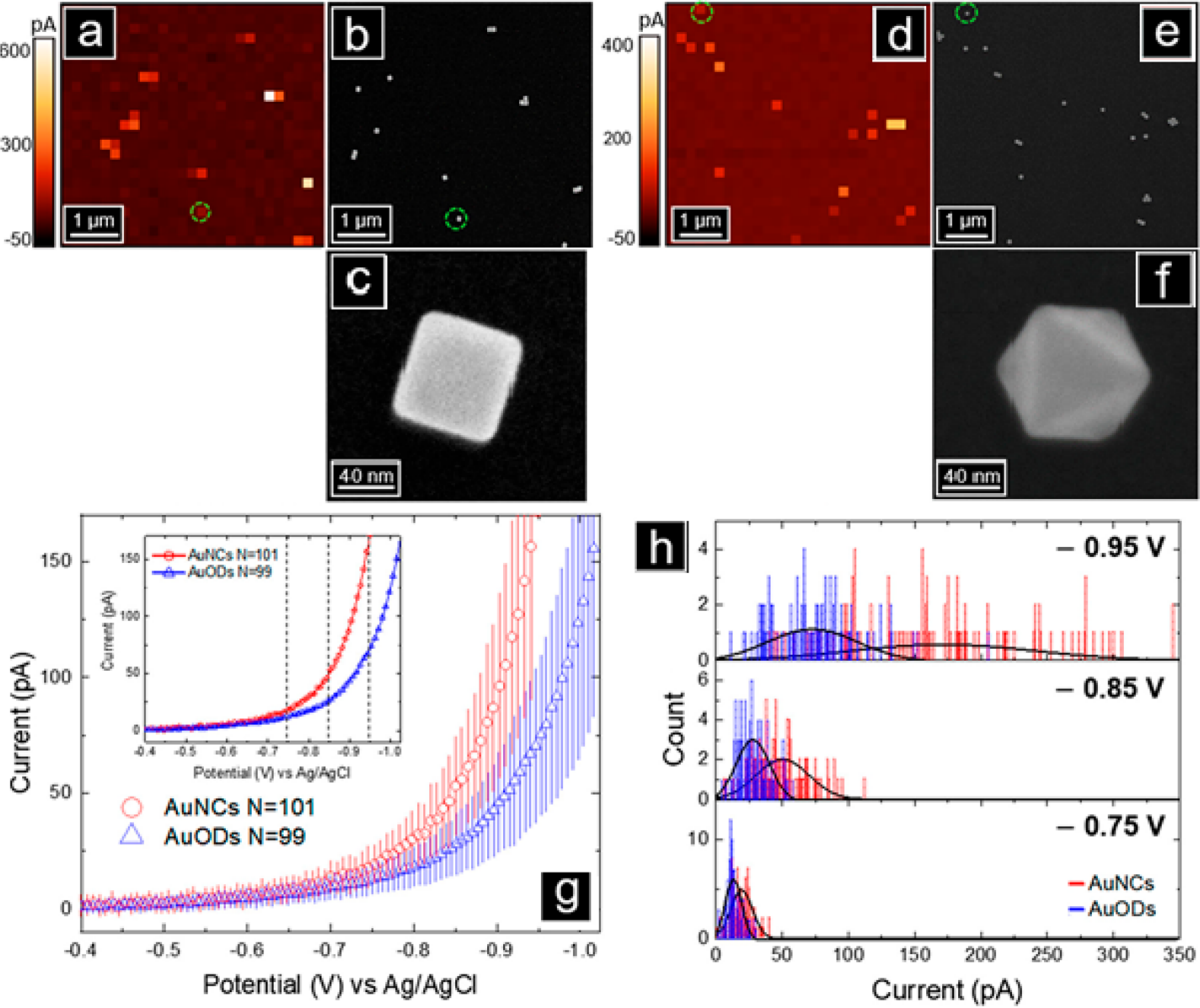
(a) SECCM CV map at −0.95 V vs Ag/AgCl, (b) correlative electron micrograph of SECCM area, and (c) high-resolution electron micrograph of Au NC. (d) SECCM CV map at −0.95 V vs Ag/AgCl, (e) correlative electron micrograph of SECCM area, and (f) high-resolution electron micrograph of Au OD. (g) Averaged CV response at individual Au NCs (*n* = 101) and Au ODs (*n* = 99). The pixel resolution of the SECCM CV map in (a,d) was 200 nm/pixel for both *X* and *Y* axes. Inset: Same averaged CVs without standard deviation errors bars. (h) Histogram of current magnitude at −0.80, −0.90, and −0.95 V vs Ag/AgCl. Adapted with permission from ref (127). Copyright 2020 American Chemical Society.

Facet-dependent electrocatalytic activity of Au nanocubes (NCs) and nano-octahedra (ODs) which are predominantly constructed of (100) and (111) crystal planes, respectively, were studied using high spatial resolution SECCM measurements (Figure 26).^127^ Single particle SECCM measurements revealed higher HER activity for Au NCs in comparison to Au ODs. DFT calculations suggested that Au (100) has a higher catalytic activity for HER as compared with the Au (110) due to lower hydrogen adsorption energy of the Au (100). The presented experimental approach in which SECCM was correlated with SEM, demonstrated the ability to measure electrocatalytic activity and visualize the particle at the nanoscale. In this manner, SECCM enables differentiation between not only single particle from a bundle but also from misshaped particles, which can be common in the synthesis of facet-controlled nanocrystals.

To conclude this subsection, facet-dependent reactivity analysis of single nanocrystals provided quantitative information about the reactivity of different facets and their interface. The facet-dependent (electro)catalytic reactivity explored on single nanocrystals demonstrated that: (1) Surface reactivity is not necessarily distributed evenly within a specific facet. (2) High spatial resolution measurements probed facet-dependent reactivity in realistic catalytic particles with complex surface morphologies. (3) Additional phenomena such as communication between facets,^121,125^ intrafacet heterogeneity,^123^ superior activity at facets interface,^120,123^ and facet induced charge separation^122^ were detected by high spatial resolution spectroscopies. (4) High spatial resolution analysis identified new nanoscale facets and quantified their influence on the reactivity pattern.

### 3.4 Composition-Dependent Reactivity

**Figure 27:**
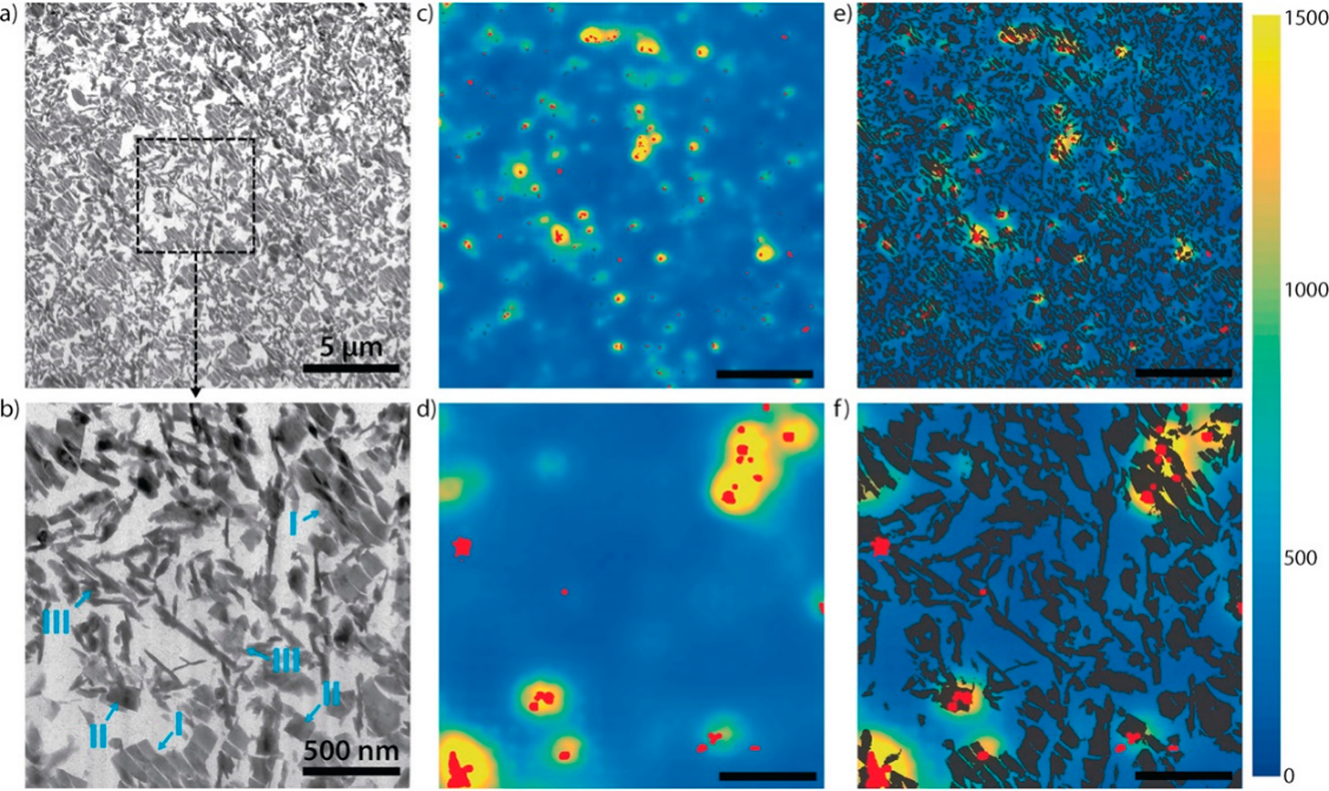
(a) TEM image of FCC catalyst and (b) higher magnification of the area marked by the square in a. Different components marked by (i) zeolite fragments, (ii) intact zeolites, and (iii) nonzeolite matrix. SOFI (according to the scale at the right side of the figure) and NASCA (red dots) mapping of fluorescence signal obtained from a and b are shown in (c) and (d), respectively. Combined fluorescence and TEM images are shown in (e) and (f). Adapted with permission from ref (130). Copyright 2019 Wiley-VCH.

The inhomogeneity in the properties of multicomponent catalytic particles calls for a method that will uncover structure and composition variations, along with reactivity analysis on a relatively large number of particles. This approach was utilized for studying fluid catalytic cracking (FCC) catalysts that are widely used in oil refining processes.^128^ The composition of the FCC catalyst includes zeolites, clay, alumina, and other materials.^129^ In light of their multicomponent nature, it is expected that the nanoscale catalytic reactivity will manifest inherent heterogeneity. Therefore, understanding which FCC surface sites contribute to higher catalytic activity is of great importance. A setup combining TEM and single molecule fluorescence microscopy was utilized to spatially resolve these sites.^130^ TEM image of single entity FCC catalysts (Figure 27a,b) enabled its classification into three components: (1) fragments of zeolite crystal created by sample preparation procedure, (2) intact zeolite crystals, and (3) nonzeolite material that served as a matrix.

Two super-resolution fluorescence microscopy techniques were implemented for mapping thiophene oligomerization as a probe reaction. Nanometer accuracy by stochastic chemical reactions (NASCA) provided localization of high intensity fluorescence events with high signal-to-noise ratio at 25 nm resolution. NASCA mapping of the fluorescent events on the FCC sample is shown by the red signal in Figure 27c,d. Super-resolution optical fluctuation imaging (SOFI) increases the spatial resolution by individually calculating the fluorescence fluctuation for each pixel and is shown by the blue to yellow scale in Figure 27c,d. SOFI and NASCA mapping were relatively correlated, yet some NASCA signals appeared without SOFI signal, attributed to nonfluctuating fluorescent events caused by contaminant species. In addition, some SOFI signals appeared with no correlating NASCA signals due to the higher sensitivity of SOFI. An overlay of the TEM image and the two fluorescence microscopy techniques is presented in Figures 27e,f. The overlay image revealed a correlation between the fluorescence signal and the zeolite crystals. However, considerable heterogeneity within and between zeolite crystals was clearly observed and might be related to different Al coordination within the crystals.^131^

Initiation of catalytic reactions depends on the efficient adsorption of reactants on active sites.^132^ The efficiency is not limited to the sheer number of molecules adsorbed per site but is also strongly related to the electronic state of the adsorbed molecules.^133^ Nanoscale TERS measurements elucidated the impact of surface composition on the adsorption affinity of reactants and its influence on chemical reactivity.^56^ TERS-STM measurements were employed to study the catalytic transformation of phenyl isocyanide (PIC) adsorbed on Au surface that is partially covered by Pd monolayer. TERS spectra were measured along the Pd–Au–Pd region and revealed site-dependent adsorption. On the Pd terrace, a dominant peak was detected at 1955 cm^–1^ and attributed to N≡C vibration, whereas on the Pd step edge, a second narrower and red-shifted peak appeared at 1933 cm^–1^. The additional peak indicates that there is a weaker N≡C bond in the PIC that is adsorbed on Pt step edge, making it prone to oxidation. Moreover, the N=C=O peak at 2245 cm^–1^ attributed to the oxidized PIC at ambient conditions is more visible at the step edge rather than the Pd or Au terrace. This is a unique example for the exploration of site-dependent reactivity by means of site-dependent adsorption of reactants and their identification via specific chemical bond shift.

**Figure 28:**
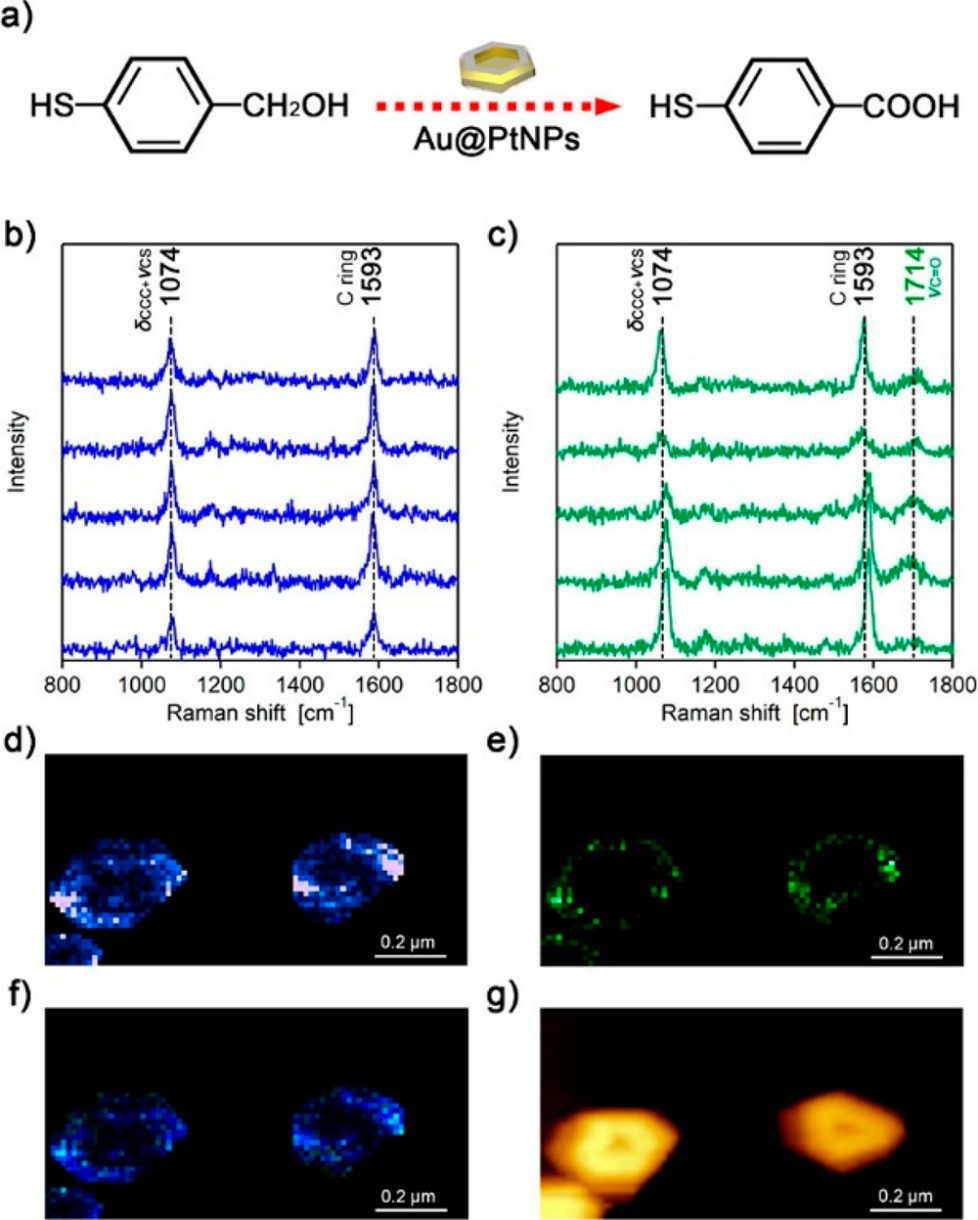
(a) Plasmon-induced oxidation of MPM to MBA on Au@PtNPs. (b,c) TERS spectra extracted from different locations of the Au@PtNPs. (d,e) Mapping of the typical vibrations for MPM (blue) and MBA (green) at 1593 and 1714 cm^–1^, respectively. (f) Overlapping TERS image of MPM and MBA. (g) AFM image of Au@PtNPs. Adapted with permission from ref (134). Copyright 2021 American Chemical Society.

Plasmon-driven oxidation and reduction reactions on bimetallic nanostructures were studied using TERS by probing the oxidation of 4-mercapto-phenyl-methanol (MPM) to 4-mercaptobenzoic acid (MBA) and the reversed reduction process on bimetallic Au@Pd and Au@Pt nanoplates (Au@PdNPs or Au@PtNPs).^134^ The carbonyl vibration at 1714 cm^–1^ has been witnessed only at the bimetallic edges, thus indicated the selective oxidation to a carboxylic acid at these sites (Figure 28). Kinetic analysis was performed by measuring the time-dependent changes in the TERS spectra. The analysis enabled extraction of the rate constants for the plasmon mediated oxidation and reduction reactions. These measurements implied that the plasmon induced reduction was faster than the oxidation.

**Figure 29:**
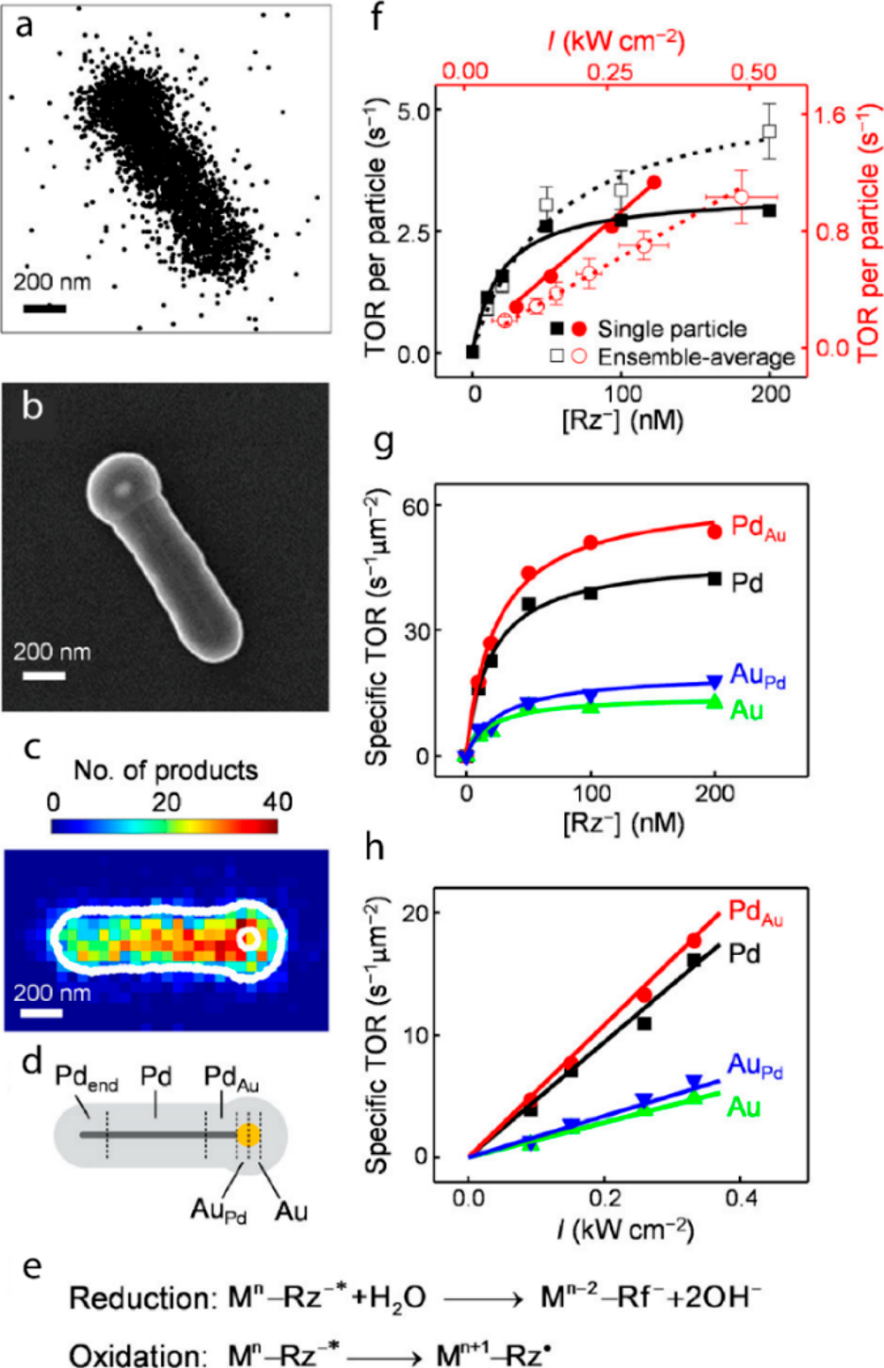
(a) Map of all fluorescence events location within a single bimetallic particle, where each event is represented by a black dot. (b) SEM image of single bimetallic particle. (c) 2D Histogram of the fluorescence events within a single bimetallic particle. White line marks the particles physical dimensions. Pixel size is according to location precision of ∼40 nm. (d) Schematic division of the catalyst into segments: Monometallic Pd at the tip far away from the Au NP (Pd_end_); monometallic Pd at the nanorod body (Pd); Au-doped Pd, close to Pd–Au interface (Pd_Au_); Pd-doped Au, close to Pd–Au interface (Au_Pd_); monometallic Au (Au). (e) Suggested kinetic mechanism. (f) Comparison of single-particle (filled circles and squared) and ensemble-averaged (hollow circles and squares) turnover rate (TOR) as a function of resazurin concentration (in black) and 532 nm exciting beam density *I* (in red). TOR of the segments defined in d as a function of resazurin concentration (g) and as a function of *I* (h). Adapted from ref (42). Copyright 2017 American Chemical Society.

Bimetallic catalysts are often known to yield a synergistic effect that leads to improved reactivity and selectivity as compared with their monometallic counterparts.^135,136^ Super-resolution fluorescence microscopy was employed to map the active sites of bimetallic Pd–Au nanostructures, providing insights to the catalytic activity of these structures compared to their monometallic components.^42^ The model system consisted of Au NP that was attached to one of the tips of a Pd nanorod and was covered by mesoporous silica shell to allow the removal of organic ligands while reactants accessibility to metallic sites remained unharmed due to the porosity of the silica. Resazurin conversion to resorufin served as the fluorescent probe reaction. Figure 29a shows the fluorescence signals collected from a single particle where each point is correlated to a single event.

The shape of the catalyst is clearly visible and matches the shape that was observed by electron microscopy (Figure 29b). The synergistic bimetallic effect was identified by a 2D histogram of the fluorescence signal and enhanced reactivity was revealed at the interface of the Pd nanorod and the Au nanoparticle (Figure 29c). Plotting the turnover rate as a function of reactant concentration or of an exciting beam density *I* revealed nonlinear and linear dependency, respectively (Figure 29e,f). These were plotted for the ensemble and at a single particle level and a classic Langmuir saturation kinetics was identified in both cases. Figure 29d shows a schematic division of the catalyst into four main segments: monometallic Pd, Au-doped Pd (Pd_Au_) near the interface, Pd-doped Au (Au_Pd_) near the interface, and monometallic Au. Notably, a plot of the specific turnover rate as a function of reactant concentration and *I* was conducted for each of these segments, revealing that Pd_Au_ sites were more reactive than monometallic Pd sites, and that Au_Pd_ sites were more reactive than monometallic Au sites (Figure 29g,h).

**Figure 30:**
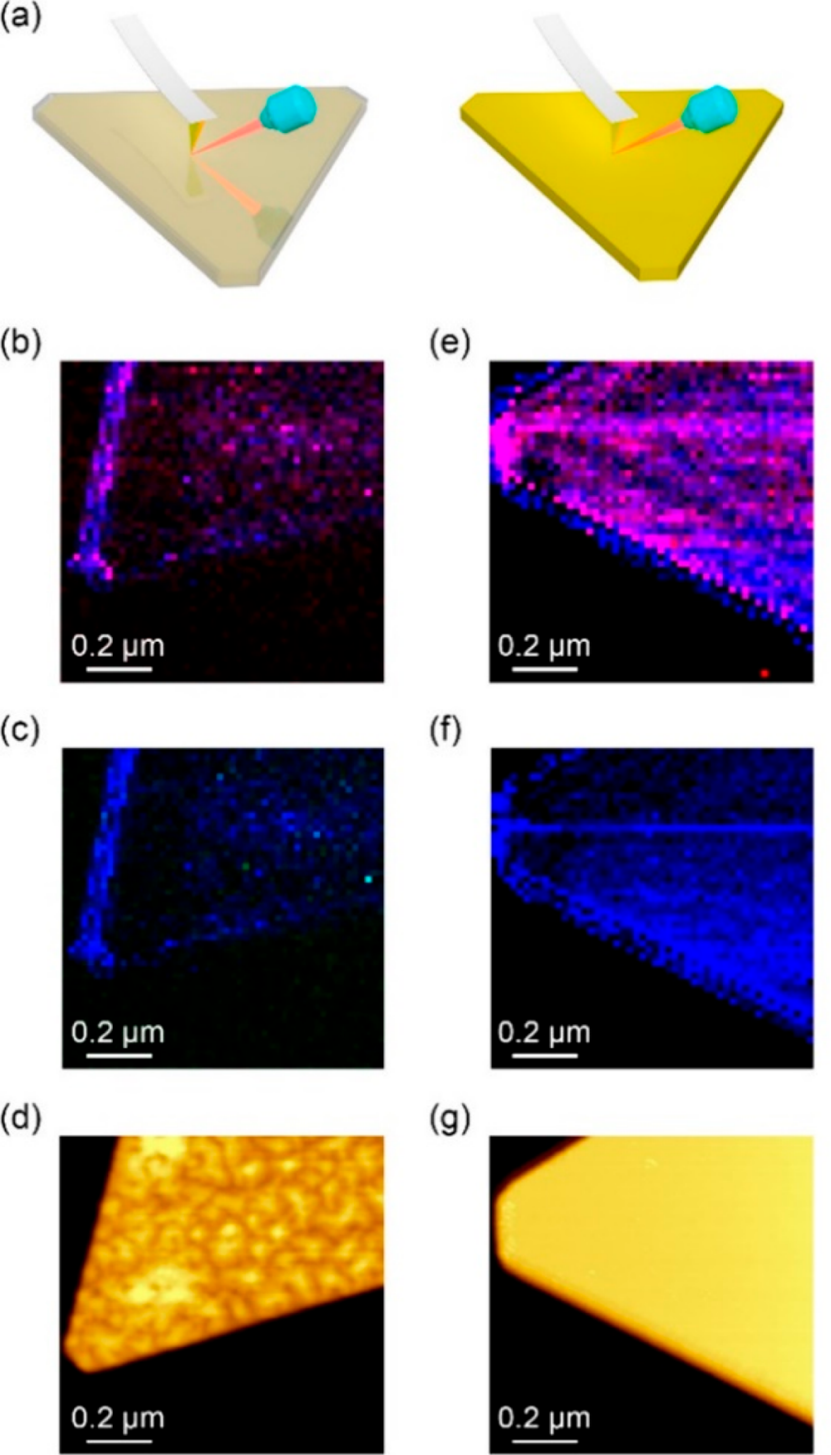
(a) Schematic illustration of Au@PdMPs and AuMPs substrates. (b,c) TERS mapping of Au@Pd MPs (20 nm per pixel). Nitro vibration of p-NTP at 1339 cm^–1^ is shown in blue, the N=N vibration at 1397 and 1441 cm^–1^ are shown in red, and 1485 cm^–1^ for the amine vibration is given in green. (d) AFM topography image of the corresponding Au@PdMPs. (e,f) TERS mapping of AuMPs (20 nm per pixel) with a similar color coding for the different vibrations. (g) AFM topography image of AuMPs. Adapted with permission from ref (137). Copyright 2020 American Chemical Society.

While the previous example showed the impact of composition on thermal reactivity by using fluorescence microscopy, the photocatalytic activity of Au microplates (AuMPs) and Au@Pd microplates (Au@PdMPs) was studied using *p*-nitrothiophenol (p-NTP) and *p*-bromobenzenethiol (BrBT) as markers for TERS detection.^137^ High spatial resolution TERS mapping revealed that despite the compositional differences both catalytic surfaces had considerable inactive domains (Figure 30). Contrastingly, the active photocatalytic nanostructures showed differences in reactivity due to the presence of Pd. For Au@PdMPs, high photoinduced reactivity toward p-NTP reduction was observed at the edges of Pd nanostructures, which confirmed that Pd is an inherent photocatalytically active metal. However, Au@PdMPs showed an overall lower reactivity in comparison to AuMPs due to enhanced damping of the localized surface plasmon resonance (LSPR) that is expected for bimetallic structures.

The effects of composition variations were widely investigated at the ensemble level, focusing on synergistic effects. However, high spatial resolution mapping of the reactivity enabled to directly identify correlations between reactivity and composition of surface sites, which is crucial due to local composition heterogeneities that can be also coupled with structural heterogeneities. Uncovering the reactive surface sites was found critical for elucidating mechanistic insights about the reactivity. For example, nanoscale analysis of the distance between the product formation site and the bimetallic site may imply whether synergistic effect includes diffusion of active species from bimetallic to monometallic sites.

## 4 Inter- and Intraparticle Diffusion

Many catalytic processes heavily rely on diffusion of active species to and from the catalytic surface. In this section, the importance of diffusion of reactants and intermediates will be demonstrated through high spatial resolution case studies that uncover the physicochemical parameters that control nanoscale diffusion and their impact on reaction mechanism.

**Figure 31:**
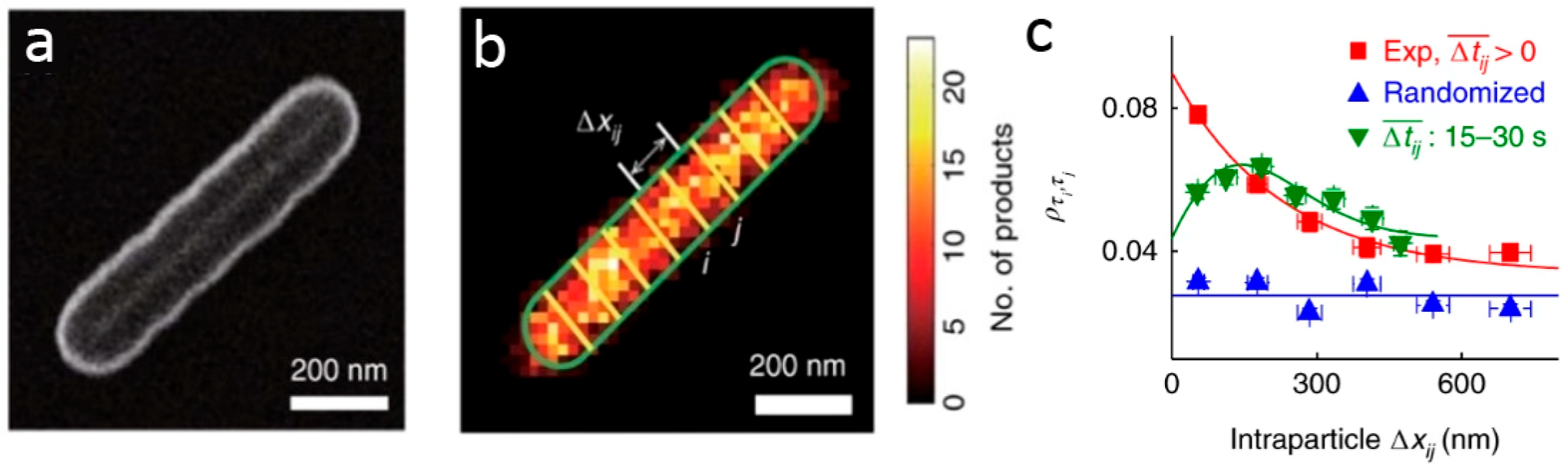
(a) SEM image of inspected mesoporous SiO_2_ coated Pd nanorod. (b) 2D Histogram of resorufin formation on single Pd nanorod. Green line denotes the structural contour (according to SEM image), yellow lines denote the dissection of the nanorod to 100 nm segments. Δ*x*_*ij*_ denotes the center to center distance between segments *i* and *jj*. (c) Plotting of Pearson cross-correlation coefficient (ρ_τ_*i*_,τ_*j*__) between reaction times (time between two fluorescent bursts at the same region) of the subsequent events of resorufin formation on Pd nanorods at region *i* and *j* (τ_*i*_,*τ*_*j*_) as a function of distance between region *i* and *j* (Δ*x*_*ij*_). In red is the data of subsequent events, in blue is randomized events, and in green when Δ*t*_*ij*_ constrained. Adapted with permission from ref (138). Copyright 2022 American Chemical Society.

Intra- and interparticle communication between different catalytic sites was probed by statistical analysis of single molecule fluorescence microscopy measurements.^45,138^ Pd nanorods, Au nanorods, and Au nanoplates were examined as nanocatalysts for various probe reactions. Intraparticle sites communication was observed by pairing catalytic event in region *i* of the catalyst to subsequent event in region *j* (Figure 31b). Next, the Pearson’s cross-correlation coefficient (ρ_τ_i_,*τ*_*j*__) between reaction times (time between two fluorescent bursts at the same region) of the subsequent events at region *i* and *j* (*τ*_*i*_,*τ*_*j*_) was computed.

Plotting the computed ρ_τ_1_,τ_2__ of over 40 Pd nanorods as a function of Δ*x*_*ij*_ (Figure 31c) revealed an exponential decay of the Pearson’s coefficient (red data points). Moreover, constraining Δ*t*_*ij*_ led to increase of the coefficient prior to the decay (green data points), indicating a positively cooperative communication between sites. Random pairing of catalytic events (blue data points) did not reveal any correlation, thus confirming that the decay is the result of communication between two subsequent events. Furthermore, no correlation of the distance or time exponential decay constants (*x*_0_^intra^,*t*_0_^intra^) to the reactant concentration was observed, therefore the communication is assumed to derive from the catalyst itself. Similar behavior was observed for Au nanorods and Au nanoplates. In addition, when voltages were applied a connection between the intraparticle communication and electric field direction was apparent. This result indicated that the catalytic messengers are positively charged and can diffuse from site to site. The detection of nanoscale communication between catalytic sites demonstrates the unique insights that are gained by conducting high spatial resolution reactivity imaging.

**Figure 32:**
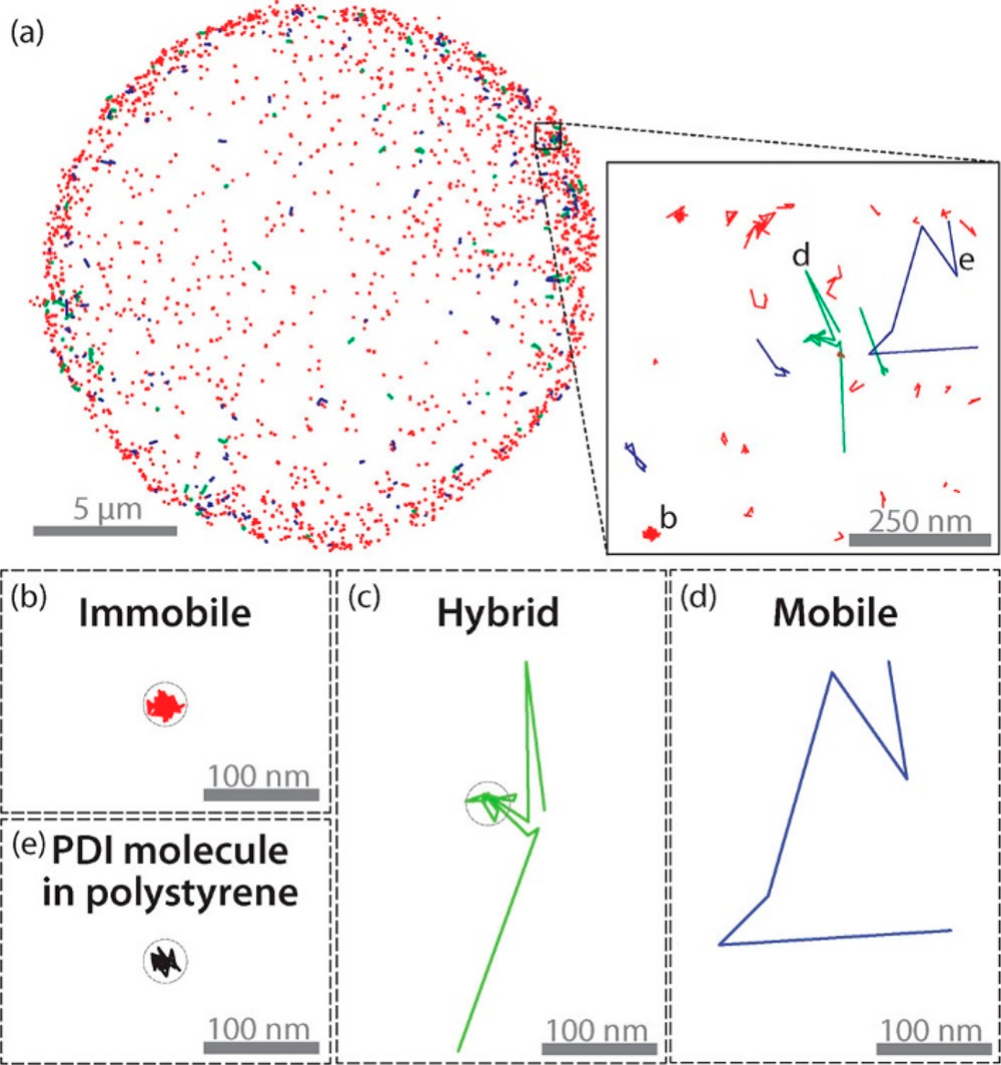
(a) Mapping of PDI track within FCC particle. Red is (b) immobile, green is (c) hybrid, and blue is (d) mobile. (e) Example of PDI molecule track in polystyrene thin film. Adapted from ref (139). Copyright 2017 American Chemical Society.

Fluid catalytic cracking (FCC) catalysts contain pores which facilitate reactant diffusion and activation. *N*,*N*′-bis(2,6-dimethylphenyl)-perylene-3,4,9,10-tetracarboxylic diimide (PDI) served as a fluorescent probe molecule to model the diffusion of oil gas to the zeolite’s pores.^139^ Fluorescence microscopy images were analyzed to determine single molecule tracks of PDI within a single FCC particle. The analysis revealed three distinct pathways for PDI. Most of the PDI molecules (88%) were immobile, i.e., presented movement within the uncertainty range or no movement at all (red in Figure 32a,b). 8% of the PDI molecules were found to be mobile (blue in Figure 32a,d), whereas the remaining 4% exhibited a hybrid behavior composed of mobile and immobile trajectories (green in Figure 32a,c). The immobility of PDI molecules was attributed to physisorption of PDI to the pore walls. It is assumed to arise from the polar groups of PDI, suggesting that a less polar molecule might exhibit improved mobility inside the pores. The diffusion coefficients of the mobile molecules were calculated, showcasing large variability that is consistent with the variability of pores size. In this regard, this work demonstrated the variation in molecular diffusion within a heterogeneous structure, a concept that induces a dominant impact on the reactivity of heterogeneous catalysis.

**Figure 33:**
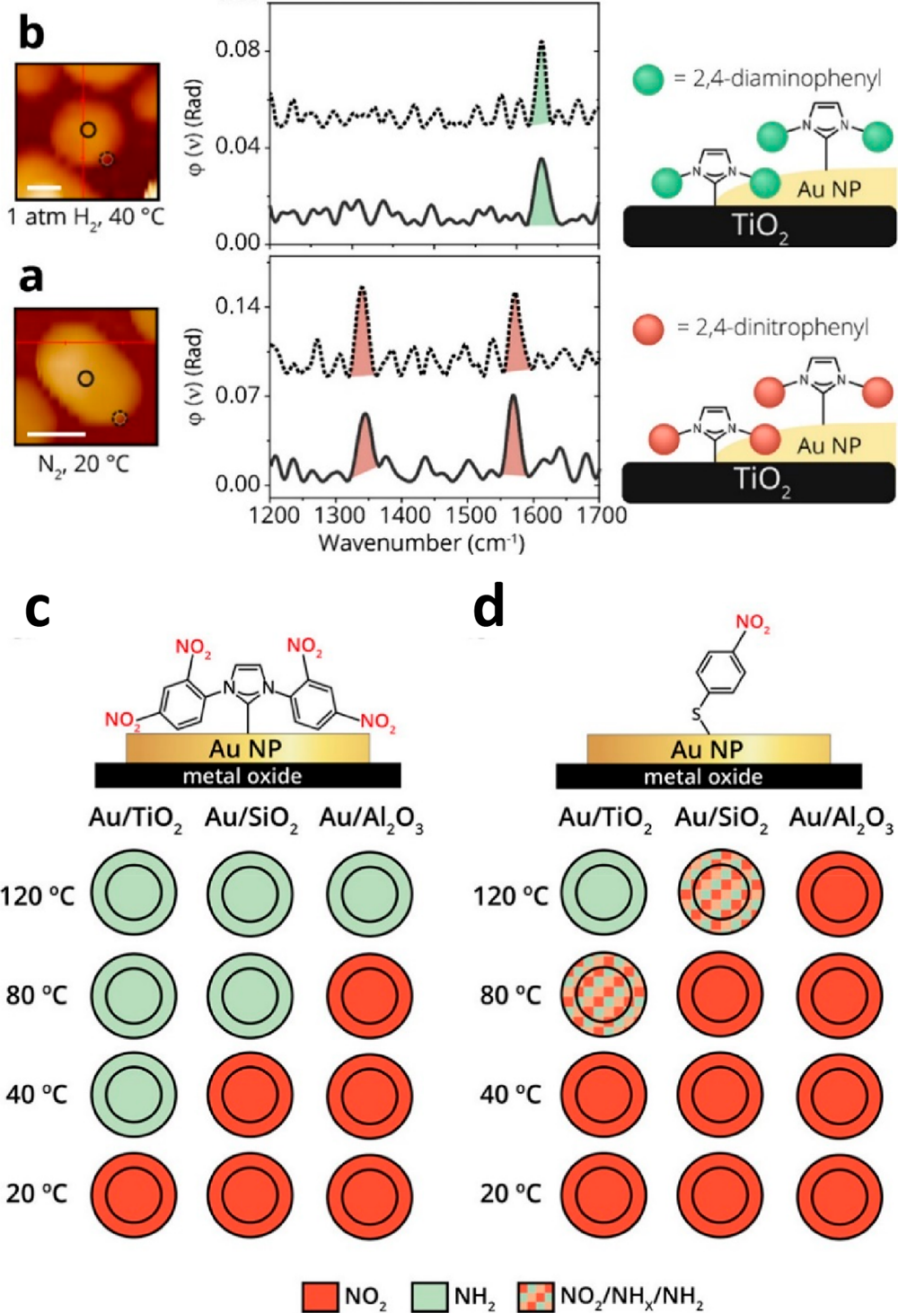
(a,b) AFM topography images (left panels) and point IR nanospectroscopy spectra (right panels) of TiO_2_-supported Au particles coated with NO_2_–NHCs. (a) IR nanospectroscopy measurements were performed following NHC surface-anchoring (b) and after exposure to reducing conditions (1 atm H_2_, 40 °C). Colored circles in the AFM topography image correspond to the IR measurements position. The same color-coding is used for the IR spectra, correlating the IR spectra with the measurement position. Scale bar is 100 nm for all AFM images. (c,d) Schematic representation of the averaged reactivity pattern toward nitro reduction of NO_2_–NHCs and p-NTP that were surface-anchored on oxide-supported Au particles. The reactivity toward nitro reduction of (c) NO_2_–NHCs and (d) p-NTP on Au particles that were dispersed on different oxides was probed by IR nanospectroscopy measurements, and a summary of the spectroscopic results is schematically illustrated. The outer ring and the inner circle correspond to the reactivity that was identified at the oxide–metal interface and on the central part of the particles, respectively. Adapted from ref (84). Copyright 2021 American Chemical Society.

The influence of intraparticle diffusion on the localized reactivity was revealed by studying hydrogenation reactions on oxide-supported Au and Pt particles, using nitro functionalized NHCs (NO_2_–NHCs) and thiols as reactivity markers. IR nanospectroscopy measurements were performed on TiO_2_-supported Au particles coated with NO_2_–NHCs.^84^ These measurements revealed site-independent nitro-to-amine hydrogenation following exposure to mild reducing conditions (Figure 33a,b). The symmetric and asymmetric stretches of the N–O bond at 1350 and 1570 cm^–1^ were replaced with the N–H bending vibration of a primary amine, signifying a full reduction of the −NO_2_ group. Site-independent reactivity pattern was observed for various oxides (Figure 33c). Notably, altering the surface ligand to *p*-nitrothiophenol (p-NTP) moderated the reactivity toward nitro reduction but did not change the overall trend of site-independent reactivity (Figure 33d). The observed trend of oxide impact (TiO_2_ > SiO_2_ > Al_2_O_3_) on the overall reactivity pattern specified that hydrogen dissociation is initiated at the metal–support interface, followed by highly efficient intraparticle hydrogen atom diffusion to the interior parts of the Au particle. These results implied that reactive Au sites on the metal–support interface and the metal surface can equally activate the nitro groups toward hydrogenation reactions in the presence of atomic hydrogen on the surface.

**Figure 34:**
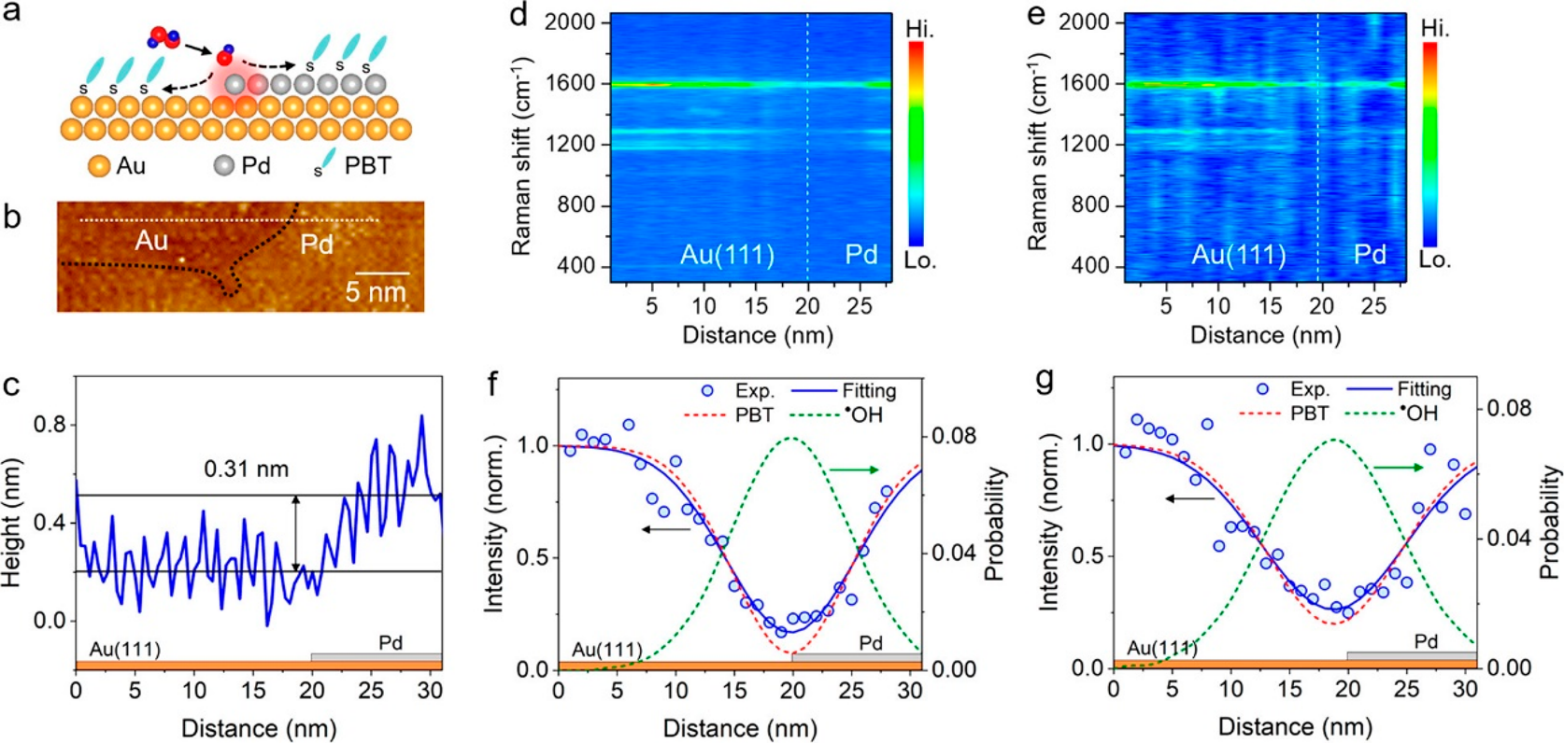
(a) Schematic illustration of H_2_O_2_ dissociation at the Au–Pd interface and diffusion to react with PBT at Au and Pd sites. (b) STM image of the Au–Pd surface region. (c) Height profile of the Au–Pd interface. (d,e) TERS intensity maps for the Au–Pd interface after exposure to mild oxidation conditions. (f,g) Spatial plotting of 1607 cm^–1^ TERS intensity from d and e (blue circles), Gaussian fitting (solid blue line), nonreacted PBT molecules distribution (red dashed line), and OH radicals presence probability distribution (green dashed line). Adapted with permission from ref (142). Copyright 2020 American Chemical Society.

Generation and diffusion of active oxygen species on a Pd/Au bimetallic surface were probed as well by TERS measurements. Evolution of reactive oxygen species such as OH radicals is often a key step in catalytic processes.^140,141^ The ability of a certain metal to produce active species may dictate whether the metal will be able to catalyze a reaction or not. However, on bimetallic surfaces, such as Au–Pd, an active species can be formed on one metal and then diffuse to the other metal and react there, as recently demonstrated by TERS measurements.^142^ Submonolayer of Pd on a well-defined Au surface served as the bimetallic catalyst surface, and (4′-(pyridin-4-yl)biphenyl-4-yl)-methanethiol (PBT) was deposited on the surface as a vibrational probe for oxidation reaction. The oxidation of PBT involves the generation of OH radical as the active oxygen specie (Figure 34a).

Exposure of the sample to H_2_O_2_ yielded a successful oxidation as implied by disappearance of the PBT’s C–C vibration at 1607 cm^–1^. TERS imaging revealed that the oxidation occurred on Pd and not on the bare Au surface, thus indicating that Pd sites are the active sites for OH radical formation. However, analysis of the TERS signal suggested that some Au sites were active despite their inability to form OH radicals. Exposure to milder oxidizing conditions was conducted to methodically examine the diffusion process. AFM measurement obtained from the Au–Pd interface (white dashed line in Figure 34b) yielded a height profile, which confirmed the location and thickness of the Pd layer (Figure 34c). The TERS intensity map revealed that PBT signal was mainly deteriorated near the Au–Pd heterojunction, indicating that the bimetallic interface is the active site for OH radical formation (Figure 34d,e). Next, the intensity of PBT signal at 1607 cm^–1^ was replotted (Figure 34f,g, respectively) and fit by a Gaussian function to spatially resolve the distribution of the nonreacted PBT molecules as well as the spatial distribution probability for OH radical presence. The symmetric Gaussian distribution behavior clearly reveals the diffusion from interface sites.

In a similar approach, hydrogen spillover on Pd/Au(111) structure was studied by conducting TERS-STM measurements of chloronitrobenzenethiol (CNBT) hydrogenation to chloroaminobenzenethiol (CABT).^58^ TERS-STM measurements revealed that although H_2_ dissociation is expected to be solely facilitated on Pd, reactivity was also probed on Au regions that are located up to ∼20 nm from the Pd–Au interface, demonstrating the influence of hydrogen spillover on the reactivity pattern.

**Figure 35:**
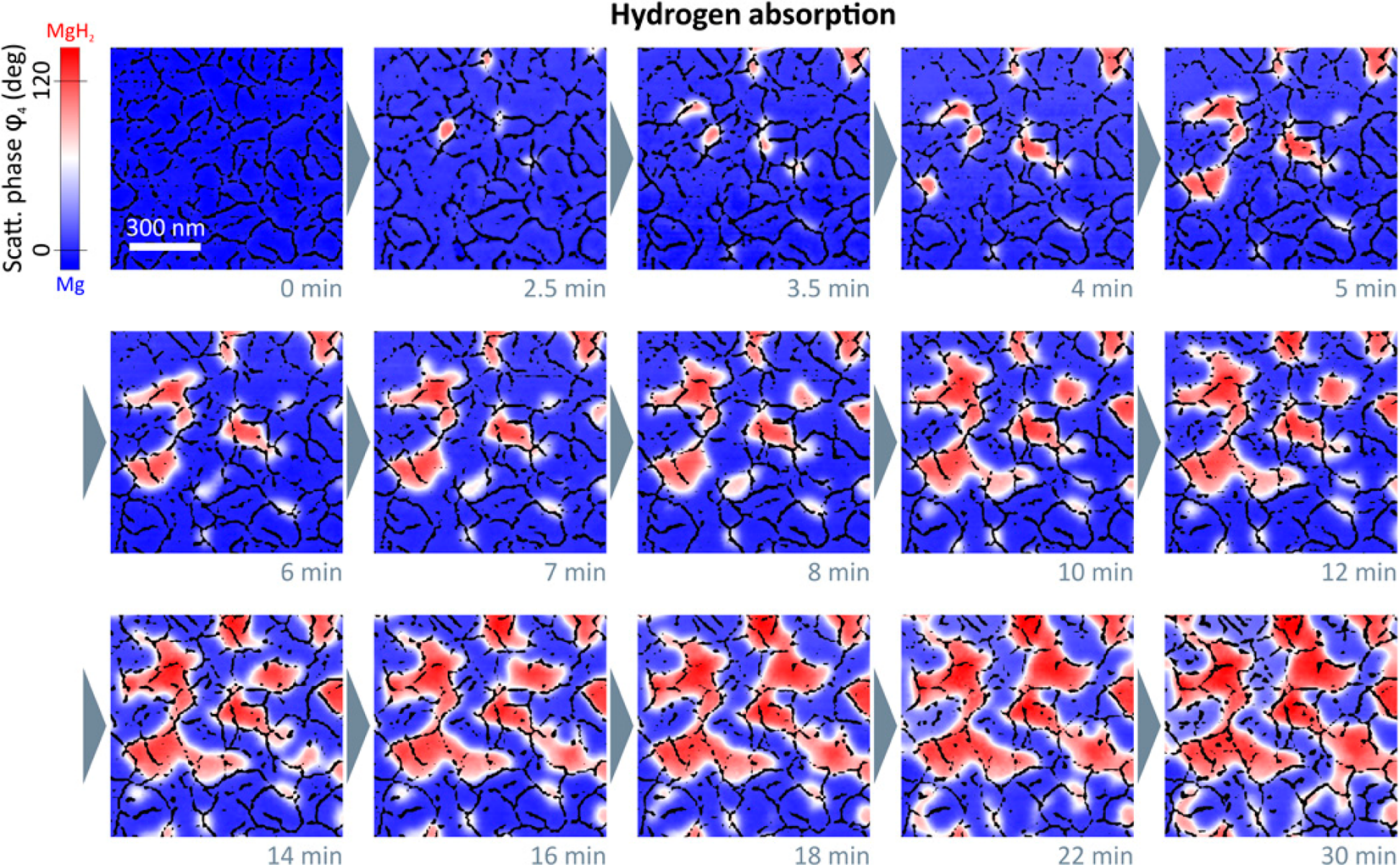
s-SNOM scattering phase imaging. Pristine Mg phase in blue, MgH_2_ phase in red. Grain boundaries overlay in black according to topography images. Adapted with permission from ref (79). Copyright 2020 American Association for the Advancement of Science.

The impact of hydrogen diffusion on localized reactivity was *in situ* investigated by s-SNOM measurements of magnesium hydrogenation, which induces phase changes from Mg to MgH_2_.^79^ The phase changes were probed by detection of MgH_2_ phonon at 1320 cm^–1^. Thin layer (50 nm) of Mg was deposited on 10 nm thick layer of Pd, which functioned as hydrogen dissociation catalyst. In this setup, hydrogen atoms flow from the Pd layer to the top surface of the Mg layer. Figure 35 shows the initiation and propagation of MgH_2_ fronts. The grain boundaries of Mg are marked according to the sample’s topography. Red spots corresponding to MgH_2_ phase were initially detected at the grain boundaries, presumably because grain boundaries are considered weak spots in the structure, allowing hydrogen atoms to penetrate from the bottom up. The MgH_2_ formation proceeded along grains until channels of the hydrogenated phase were formed. The hydrogenation process was limited to about 50% of the film. It was suggested that the newly formed MgH_2_ serves as a hydrogen diffusion barrier, an active “blocking layer” that is responsible for the autotermination of the hydrogenation process.

The presented studies in this subsection demonstrated the influence of diffusion of reactants, products, and intermediates on the localized reactivity and the impact of nanoscale diffusion on the reactivity of neighboring sites, which are characterized with lower reactivity.

## 5 Reaction Mechanism Analysis

So far, the focus of the works described above was to identify surface active sites and to provide insights gained from the catalyst perspective. Nevertheless, high spatial resolution techniques can also expand the ways in which reaction kinetic is measured. Collecting kinetic data from single catalytic particles and comparing it to the measured kinetic trends observed for the ensemble is a powerful tool that can strengthen theoretical reaction models.

**Figure 36:**
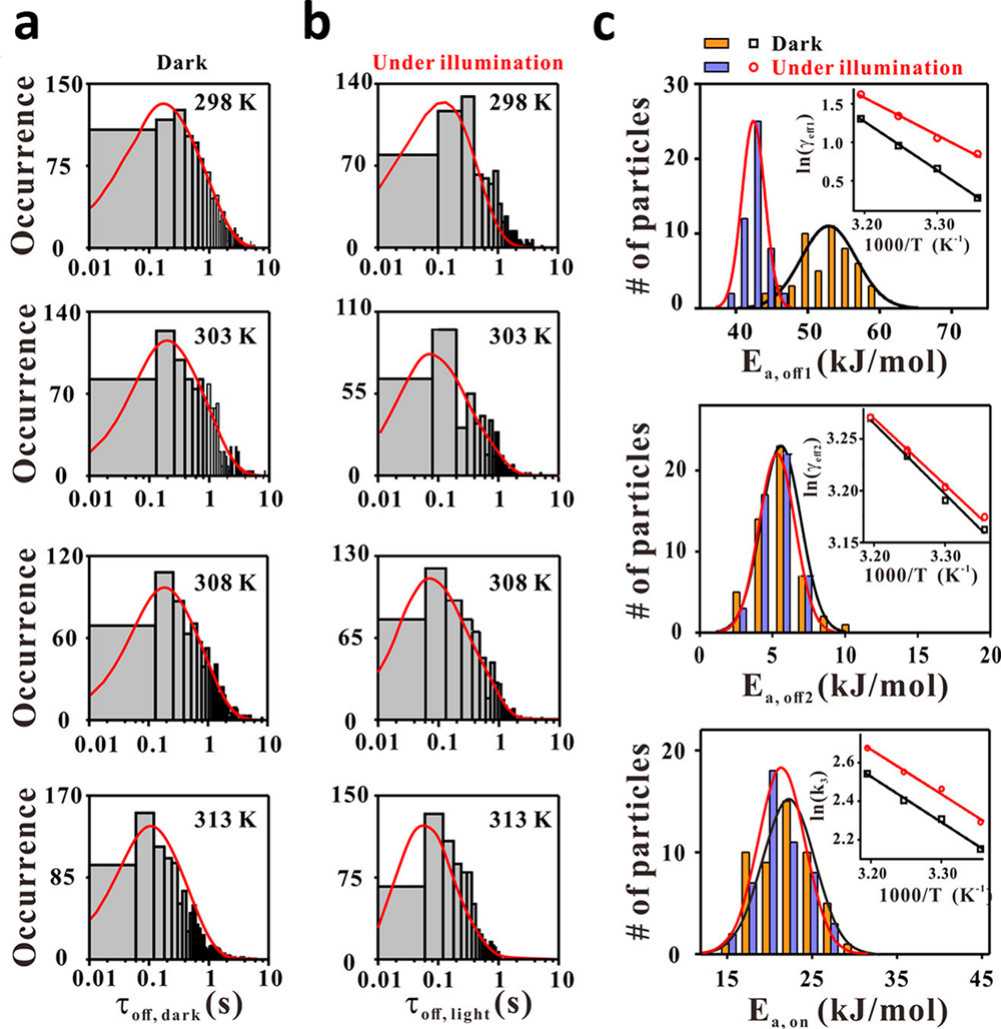
Distribution of τ_off_ at different temperatures at dark condition (a) and under illumination at 758 nm (b). Fitting of probability density function is in red. (c) Distribution of activation energies for the intermediate formation (top), final product formation (medium), and product desorption (bottom) calculated from over 100 Au NRs. Insets: average Arrhenius plots under dark (black) and laser illumination (red). Adapted with permission from ref (143). Copyright 2020 American Chemical Society.

Fluorescence microscopy measurements enabled extraction of the reaction rate and activation energy values on various surface sites,^143^ using the two-step AR oxidation to resorufin on Au nanorods as a model reaction.^144^ Fluorescence bursts of single product formation were detected, where the burst length denoted by τ_on_ represents the desorption time. The time between two subsequent bursts is denoted by τ_off_ and includes the substrate adsorption, intermediate formation, and product formation. Because the intermediate is nonfluorescent, it is not possible to directly track its formation. However, according to the Langmuir–Hinshelwood noncompetitive mechanism, the probability density function of τ_off_ for two steps reaction will depend on rate constants *k*_1_ and *k*_2_. Thus, fitting the distribution density function to the τ_off_ distribution at different temperatures (Figure 36a,b) allows extraction of *k*_1_ and *k*_2_ values, and by extension, the activation energy of each step from the Arrhenius equation.

The τ_off_ distributions were collected with and without illumination at 785 nm to observe the difference derived from plasmon-enhanced catalysis. Activation energy distributions revealed that the activation energies for resorufin formation and desorption remain unchanged with and without illumination (Figure 36c, middle and bottom). AR activation energy was found to be higher than the energy barriers of resorufin formation and desorption, therefore it was concluded that this is the rate limiting step. In a similar way, fluorescence microscopy measurements were utilized to analyze and decouple on-surface product formation and its subsequent desorption by studying the reduction reaction of resasurin to resorufin on Au NPs.^145^ The calculation unveiled resorufin formation as the rate-limiting step, with calculated activation energy values close to the one recorded by ensemble measurements. However, large variability was observed within a single particle, attributed to the presence of different surface sites.

**Figure 37:**
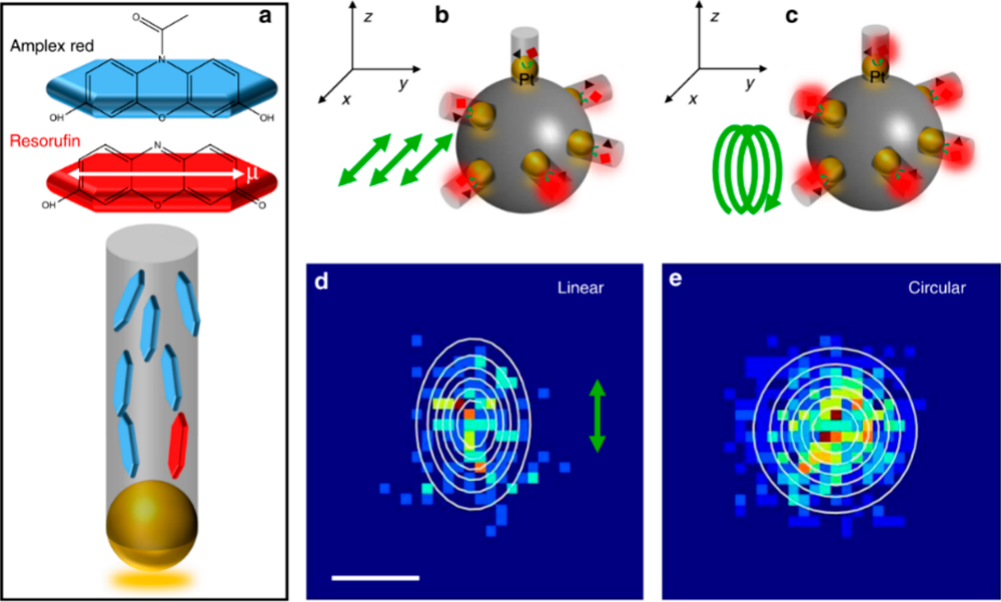
(a) Schematic illustration of the ways by which silica pores may dictate standing orientation of AR (blue) and resorufin (red) with respect to the Pt NP (yellow particle). (b) Schematic illustration of the catalyst core–shell structure and direction of linear polarized excitation and (c) Circular polarized excitation. (d) Super-resolution imaging of resorufin detection on single particle under linear polarized excitation and (e) circular polarized excitation. Scale bar is 200 nm. Adapted with permission from ref (146). Copyright 2019 Nature Publication Group.

Catalytic processes are often influenced by the adsorption orientation of nonspherical reactants, as it may affect both the adsorption/desorption rates and the reactivity of adsorbates. These principals were demonstrated by using a well-defined modified catalyst structure that was designed to confine reactants and intermediates.^146^ The structure of the catalyst was constructed of solid SiO_2_ core with mesoporous SiO_2_ shell containing linear pores, with Pt NPs in between the core and the shell (Figure 37). Conversion of AR to resorufin was chosen as the probe reaction because AR is fluorescent and both reactant and product are nonspherical molecules. Kinetic observations probed enhanced catalytic activity as the shell thickness increased. The role of the pores in facilitating the activity was attributed to confinement of AR molecules (Figure 37a). Applying linearly polarized excitation beam (Figure 37b,d) and circular polarized excitation (Figure 37c,e) revealed elliptical distribution of products along the applied linear polarization and symmetric circular distribution when circular polarization was applied. The linear polarized excitation should only detect resorufin molecules where the dipole moment is aligned with the excitation direction, while the circular polarized excitation leads to detection of all resorufin products. Therefore, the experimental results revealed that the pores confine the molecular orientation to standing orientation, leading to assumption that the AR molecules are adsorbed in the same orientation. This orientation induces high proximity of the phenol group to the active oxygen species which can lead to elevated reactivity.

**Figure 38:**
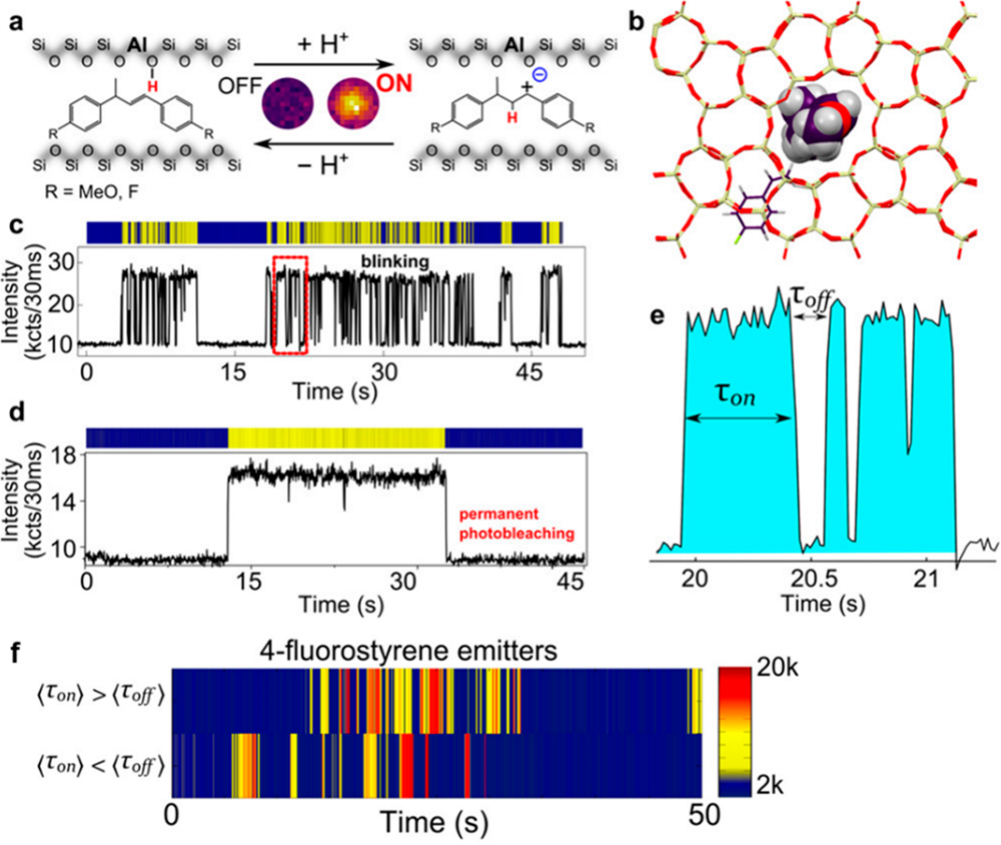
(a) Schematic of the approach to detect protonated, fluorescent dimeric carbocation (bright state on the right); the dark, nonfluorescent state (left) is a neutral dimer that is formed after a proton transfer to the zeolite framework. (b) Representation of the zeolite framework (view along *b* axis) and a dimeric carbocation (ball and stick model) trapped along the straight pores; additional monomeric styrene residing in a sinusoidal pore is shown (wire model). (c,d) Blinking (c) and nonblinking (d) fluorescence intensity trajectories of individual carbocationic species of 4-methoxystyrene in heptane. (e) A zoom-in of the trajectory shown in (c), indicating the definition of τ_on_ and τ_off_ times. (f) Statistical description of blinking properties for fluorescent products originating from 4-fluorostyrene. Adapted with permission from ref (147). Copyright 2018 American Chemical Society.

Fluorescence microscopy measurements were utilized to probe the impact of intermediates on catalytic reactions in zeolites. Oligomerization of styrene derivatives, catalyzed by ZSM-5 zeolites, constitute a convenient model system to study reaction intermediates.^147^ In this reaction, the dimeric carbocation function as an intermediate and emits light within the visible range,^148^ making it suitable for probing by fluorescence microscopy (Figure 38). The fluorescence trajectories of the carbocation exhibited “blinking”, i.e., turning on and off as a result of switching between the carbocation and a neutral state (Figure 38f). Two styrene derivatives 4-fluorostyrene and 4-methoxystyrene were examined. For each “blink” the on time (τ_on_) and subsequent off time (τ_off_) were recorded. It is revealed that τ_on_ and τ_off_ obtained for fluorinated molecules showed similar values, while τ_on_ was higher than τ_off_ for the methoxy substituted molecules. In addition, the τ_on_ of the methoxy substituted carbocations were longer than that of the fluorinated carbocations. These results suggest that the carbocation intermediate is more stable when the styrene is functionalized with a methoxy substituent. The latter is consistent with known electronic substituents effects, in which the methoxy group stabilizes a carbocation while the fluoro group destabilizes it.

**Figure 39:**
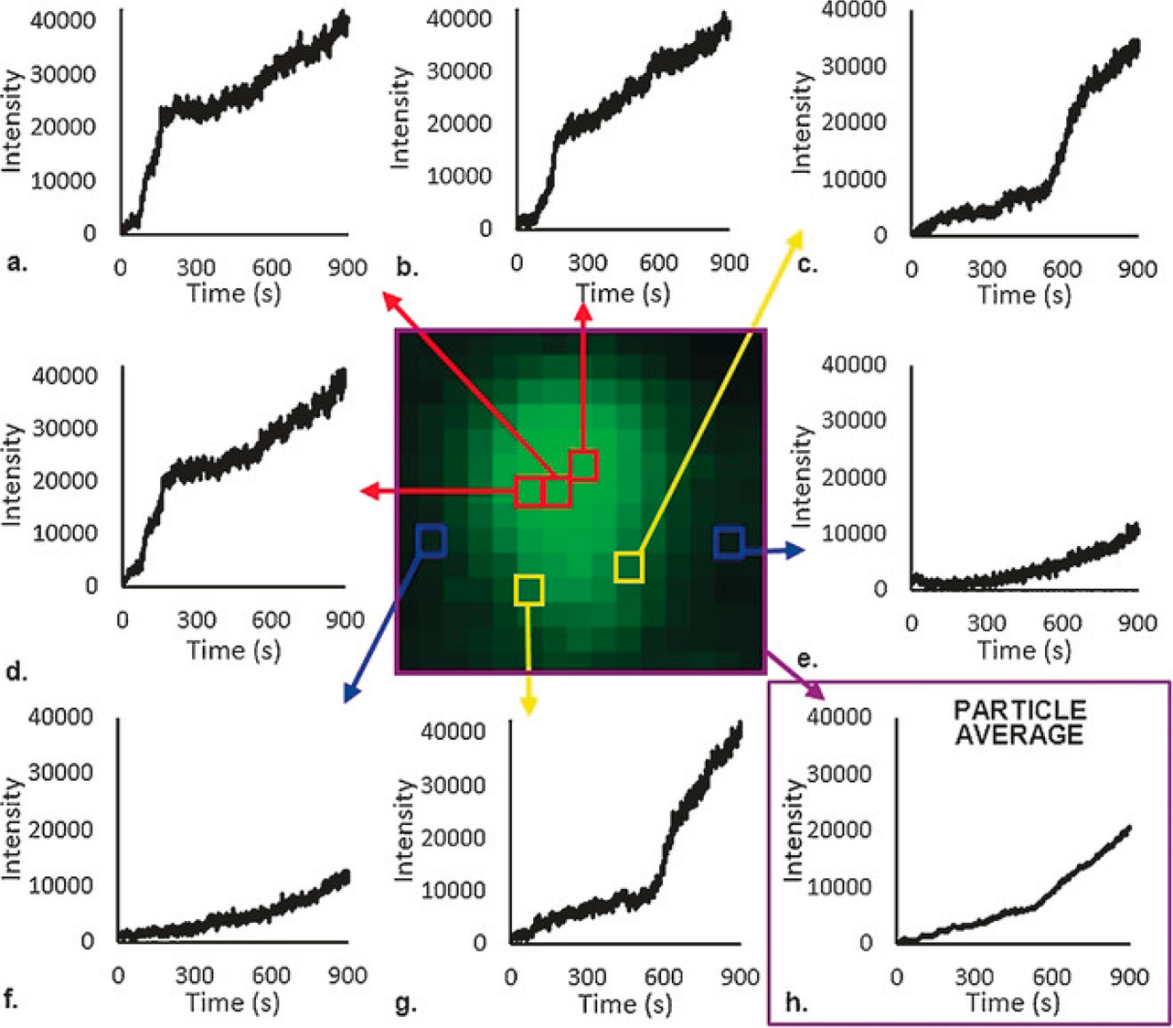
Subparticle polymerization kinetics showing different rates and variable kinetic states in different submicrometer regions of a single polynorbornene aggregate. Each pixel = 267 × 267 nm^2^. Adapted with permission from ref (149). Copyright 2018 Wiley-VCH.

Time-resolved fluorescence microscopy was utilized for measuring the kinetics of a polymerization reaction catalyzed by a molecular catalyst at a single particle level.^149^ Namely, the fluorescence intensity was measured over time for a monomer insertion reaction at the submicrometer regions of a single polymer aggregate. It was shown that at the single ruthenium catalyst level, the kinetics of ring-opening metathesis polymerization, catalyzed by second-generation Grubbs ruthenium catalyst, is dynamic with respect to time. Figure 39 shows the fluorescence intensity over time for different submicron regions within a single polymer aggregate. Notably, the kinetic profile is represented by the slope of the graph, and each subparticle region shows variable kinetic profile with respect to time. These variations were attributed to inhomogeneity in the accessibility of monomers to the center of the catalyst and demonstrate that local variations in the solution can impact the reactivity of homogeneous catalysts.

Catalysts are often broadly divided into homogeneous or heterogeneous categories, according to whether the catalyst is in the same phase of the reactant medium or not. However, it is possible for some catalytic systems to exhibit intermediary behavior so-called “semiheterogeneous”. Such a behavior can be a result of metal ions leaching to the solution from metal NP, thus leading to formation of catalytically active ionic species.^150^ Another option for this intermediary behavior is the opposite case where homogeneous complexes react to form solid NPs under reaction conditions.^151^ An additional mechanism for “semiheterogeneous” catalysis was probed for Sm_2_O_3_ NPs in the Pechman transesterification and condensation of 8-hydroxyjulolidine and ethyl 4,4,4-trifluoroacetoacetate to coumarin 153.^152^ Because coumarin 153 is a fluorescent molecule, its formation can be detected by fluorescence microscopy. Detection of the location of fluorescence signals enabled identification of the nature of the catalyst. Surface-supported heterogeneous catalysts should provide signal at discrete locations. Conversely, homogeneous catalysts will yield randomly distributed product signal.

**Figure 40:**
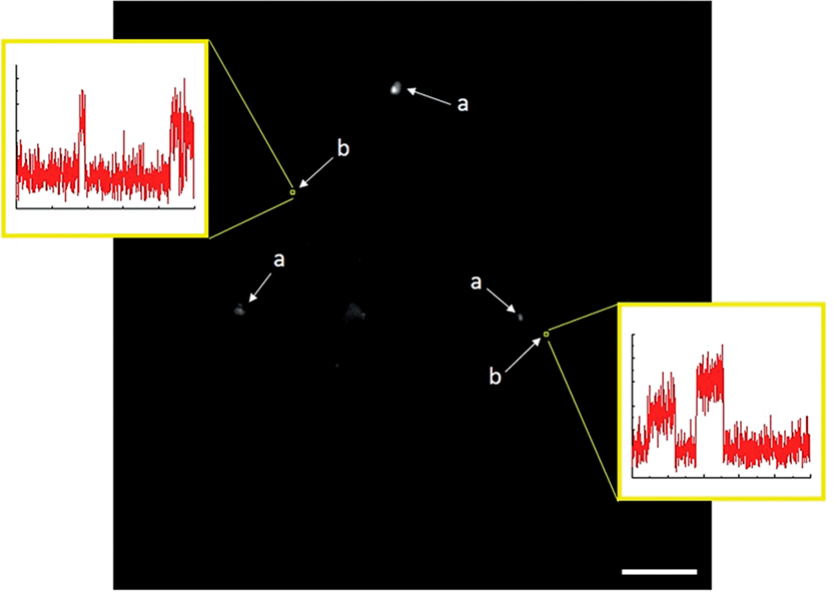
Single frame from a fluorescence microscopy image sequence recorded while flowing the reactants atop a coverslip spin-coated with Sm_2_O_3_ nanoparticles. Large Sm_2_O_3_ nanoparticles are visible due to scattering (a), and multiple fluorescence bursting is observed in regions where no large Sm_2_O_3_ nanoparticles are located (b). Scale bar is 10 μm. Adapted with permission from ref (152) Copyright 2016 Royal Society of Chemistry.

Fluorescence microscopy measurements demonstrated that polydisperse Sm_2_O_3_ nanoparticles provide a continuous supply of colloidal heterogeneous catalyst. Intensity–time trajectories corresponding to image sequences recorded while flowing the reactants atop a microscope coverslip spin-coated with solid Sm_2_O_3_ nanoparticles showed repetitive bursting (Figure 40). This occurred specifically in areas where large Sm_2_O_3_ NPs were not located. It was therefore concluded that products were not formed in the solution, but mostly by heterogeneous catalysis. Nevertheless, fluorescence signals did not correlate to the original location of Sm_2_O_3_ particles, due to a mobile population of small Sm_2_O_3_ NP released from a polydisperse sample containing larger particles. Fluorescence microscopy therefore provided unique insights about the reaction mechanism, identifying that larger particles are merely suppliers for smaller and highly reactive catalytic nanostructures.

**Figure 41:**
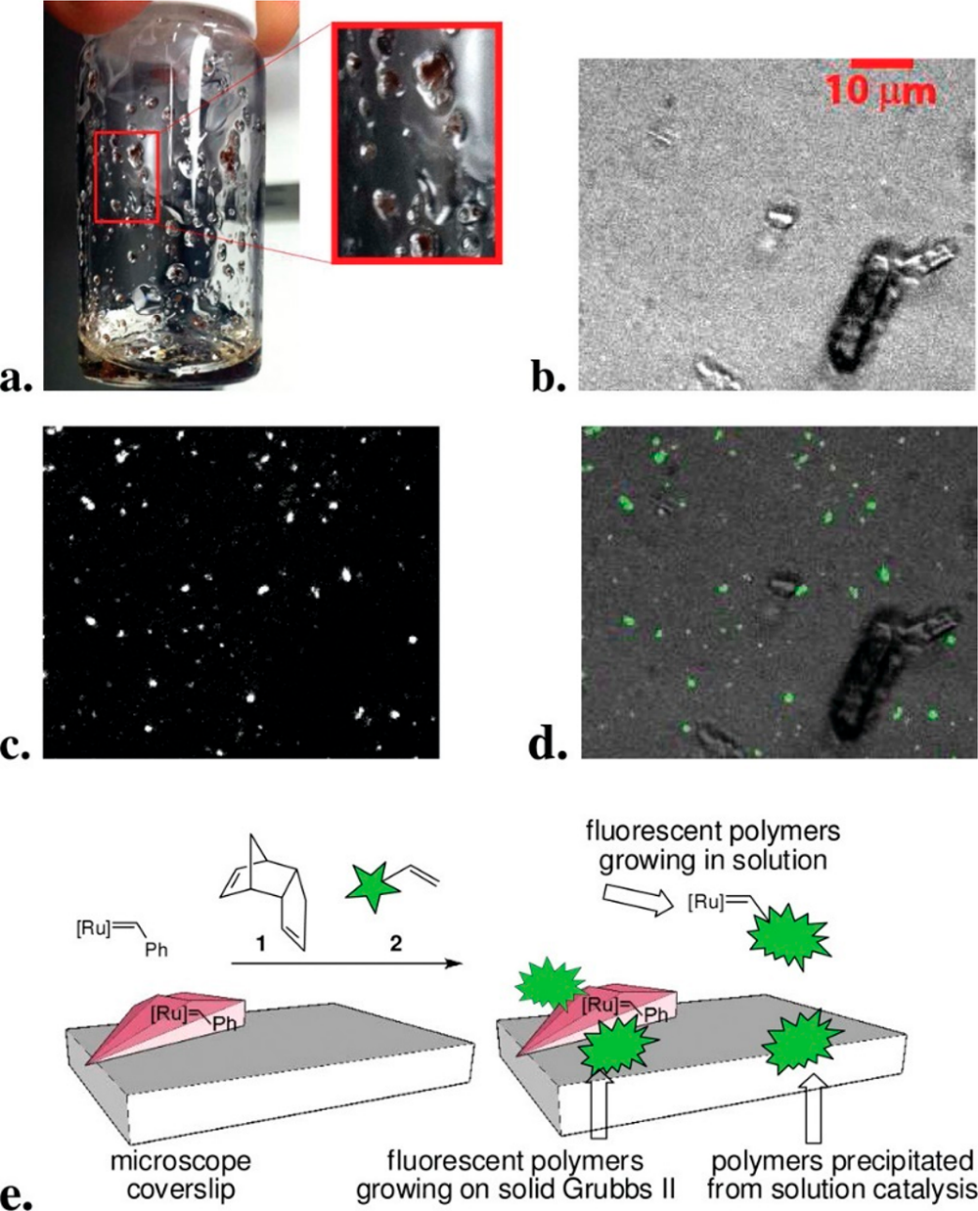
(a) Photograph of bench-scale experiment. Dicyclopentadiene polymerizes around solid particles of Grubbs II, encapsulating the solid maroon-colored precatalyst in clear polydicyclopentadiene. (b) A 53 μm × 63 μm microscope image with ambient light, showing individual crystals of Grubbs II on the surface of a glass microscope slide. (c) Same surface region as in (b) but fluorescence image at *t* = 170 s; individual polymer particles have precipitated on the microscope slide after reaching insolubility in solution. Each white spot is one polymer particle. (d) Overlay of (b) and (c) reveals that polymer growth is not spatially associated with solid particles of Grubbs II precatalyst. Fluorescent polymers are false colored green to facilitate spatial comparison. (e) Experiment schematics: fluorescent polymers tagged with BODIPY (green star) form upon polymerization of **1**. The location of the polymerization differentiates between homogeneous and heterogeneous catalysis. Adapted with permission from ref (18). Copyright 2011 Wiley-VCH.

The determination of whether a catalytic process is heterogeneous, homogeneous, or both is not trivial because the existence of a solid or soluble catalyst components does not indicate on the actual active phase during the catalytic process. Fluorescence microscopy measurements were conducted to identify the connection between the location of a solid catalyst and the actual catalytic reactivity^18^ while using a molecular Grubbs catalyst for polymerization reaction of dicyclopentadiene as a model reaction and boron dipyrromethene molecules (BODIPY) as fluorophores for product formation (Figure 41). High spatial resolution image revealed that the location of the solid catalyst and the polymerized particles are not spatially related, hence it was concluded that the catalysis is homogeneous. In this work, single event fluorescence measurements provided a straightforward approach for differentiation between homogeneous and heterogeneous catalysts.

**Figure 42:**
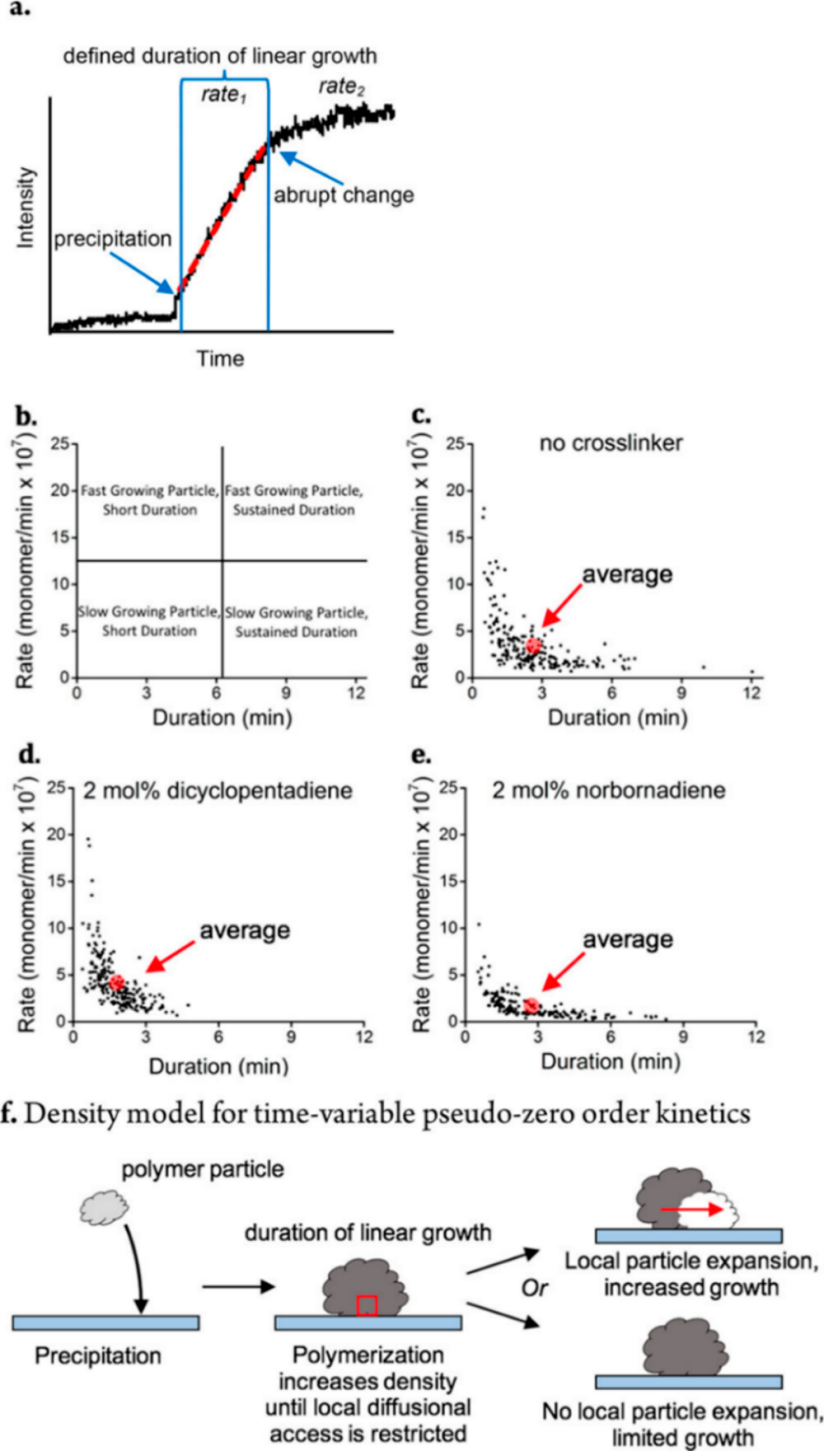
(a) Representative example of intensity versus time trace, depicting how duration of linear growth was quantified. The red line indicates the linear fit of the pseudozero-order polymerization kinetics. (b) Model scatter plot for polymerization, illustrating different types of kinetic behavior. Scatter plot for polymerization of (c) norbornene without cross-linking agent, (d) norbornene with 2 mol % dicyclopentadiene, and (e) norbornene with 2 mol % norbornadiene. The red data point represents the average value for each experiment type. (f) Consistent physical model for abruptly changing kinetics. The red square represents a subparticle imaging region for microscopy. Adapted with permission from ref (19). Copyright 2019 Wiley-VCH.

The kinetics of a ring-opening metathesis polymerization (ROMP) reaction, activated by second-generation Grubbs ruthenium catalyst, was studied at the single catalyst level and was shown to be highly dynamic in its nature.^149^ The existence of a variable local environment was suggested as the cause for local heterogeneties.^153^ In order to establish a physical model that can clarify the reason for the dynamic kinetic behavior, the duration and rate of the linear growth regions were analyzed (Figure 42a).^19^ Correlation analysis between the linear growth parameters, the reaction rate and duration (Figure 42b–e) has enabled gaining of meaningful insights regarding the growth mechanism of polymeric particles. It was identified that fast growing particles do not lead to sustained growth duration. Based on those insights, a physical model was suggested (Figure 42f). The comparison of the hypothetical “average” measurements and the subparticle level measurements, represented by the red and the black dots, respectively, reveals the significant contribution of the high spatial resolution for mechanistic analysis.

**Figure 43:**
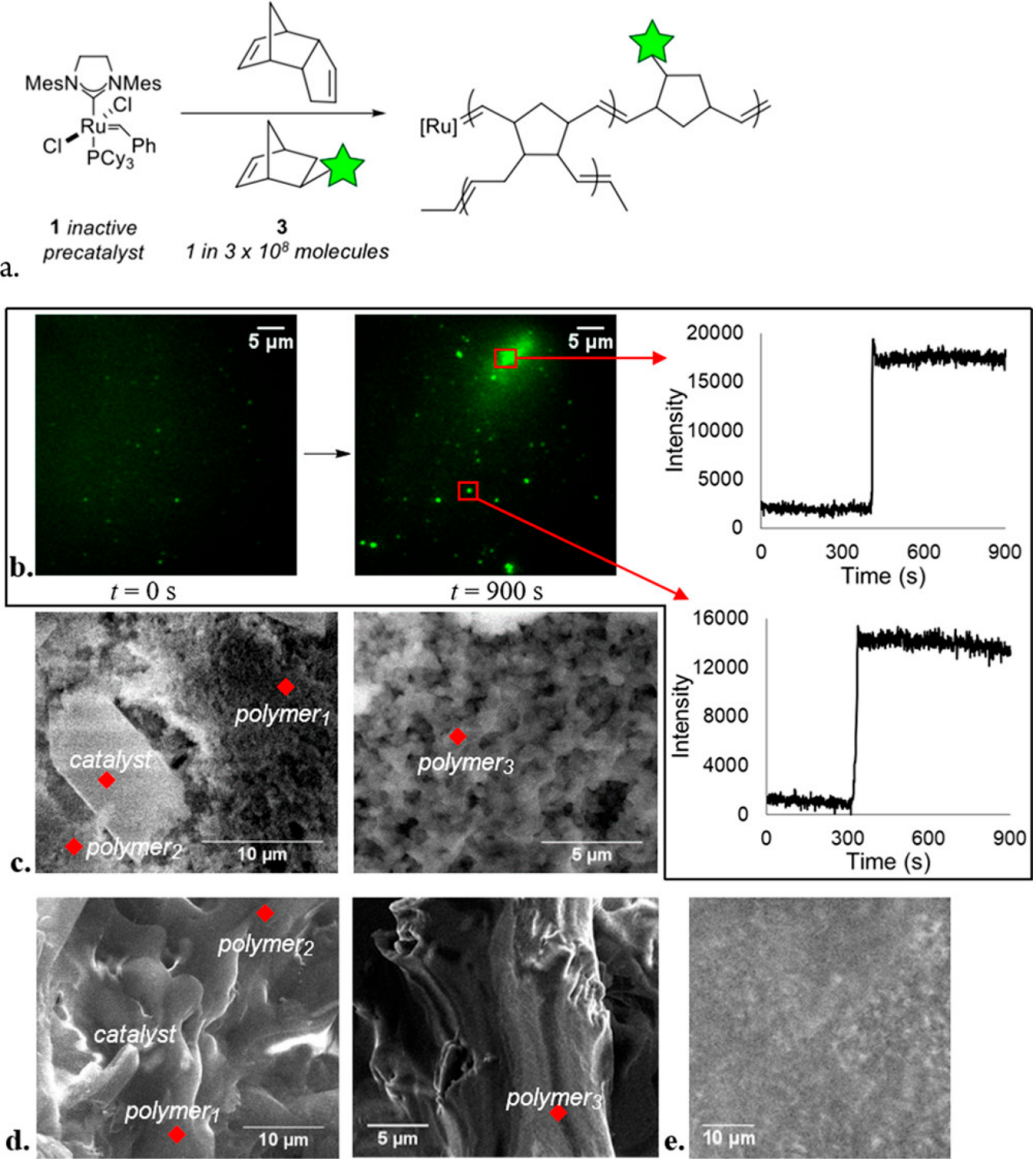
(a) Reaction equation, showing molecular precatalyst **1** polymerizing dicyclopentadiene in the presence of small quantities of imaging agent **3**, yielding polydicyclopentadiene copolymerized with imaging agent **3**. (b) Imaging agent **3** is incorporated into polydicyclopentadiene by catalyst **1**; polymer brightness does not increase after abrupt precipitation, as seen via time trace data. SEM micrographs of (c) polydicyclopentadiene and (d) polynorbornene. Solid forms of catalyst crystals can be easily differentiated from polymer on the basis of their different shapes and structures. Red dots show locations of three EDS measurements within three regions of polymer. (e) Polydicyclopentadiene visible on the surface of a glass coverslip via optical transmission microscopy confirmed its presence. Adapted with permission from ref (19). Copyright 2019 Wiley-VCH.

An additional challenge in the field of homogeneous catalysis is the determination of whether a molecular catalyst is in its active or inactive state. This subject was addressed by combining SEM/EDS and fluorescence microscopy, which enabled imaging at the subensemble level. The investigated model reaction was the precipitation of polydicyclopentadiene (Figure 43).^149^

The catalytic activity of the molecular ruthenium catalysts in the polydicyclopentadiene system was lower than the polynorbornene system. This finding can be explained by a lower amount of the active form of the catalyst within the polynorbornene aggregates because only the active form can give a fluorescence signal. In order to quantitively assess the general amount of the ruthenium catalyst in the polymeric aggregates, SEM and EDS measurements were utilized. Figure 43c,d present SEM images in the subparticle level, when the catalyst and the polymer can be spatially differentiated. EDS analysis has revealed that the catalyst quantity in the polydicyclopentadiene system was higher than the polynorbornene system, even though the former has shown a lower catalytic reactivity. These findings can be explained by the presence of a higher amount of the inactive form of the catalyst in the polydicyclopentadiene system.

As demonstrated in this section, the study of single-site reaction mechanism demands the technical capability to explore the interaction between a catalytic site and active molecular species. In many cases, the nature of this interaction is dynamic with respect to time and space due to the motion of the active species relative to the corresponding position of the catalyst. This dynamic interaction may also be highly heterogeneous at the intra- and intercatalyst level due to variations in the local environment of the catalytic sites. High spatial resolution analysis provided the capabilities to identify the reactive sites on which the reaction was facilitated and the kinetics on these sites.

## 6 Concluding Remarks and Outlook

### 6.1 Mechanistic Insights Gained by High Resolution Reactivity Measurements

High spatial resolution studies highlight the coexistence of various active sites in heterogeneous and homogeneous (electro)catalysis. Experimental evidence was provided for the crucial role of defect sites in thermal catalysis,^83,95,96^ photocatalysis,^104,120,134^ and electrocatalysis^105−107^ by directly mapping the reactivity on surface sites. In addition, inter- and intraparticle effects, induced by spillover and diffusion, were identified,^45,84,139^ along with analysis of the impact of composition and oxidation-state variations on the localized reactivity.^111^

Kinetic data about the reaction mechanism has been mostly gained by using fluorescence microscopy measurements. One of the most common approaches for fluorescence microscopy analysis is based on breaking down the catalytic process by tracking the fluorescence trajectories, when τ_on_ represents the product desorption time and τ_off_ include reactant adsorption time, intermediate formation, and adsorbed product formation. Yet, τ_off_ may also be influenced by events in neighboring sites, such as diffusion to and from active sites. τ_off_ analysis has been mostly based on the Langmuir–Hinshelwood kinetics model. Direct segmentation of reaction steps is possible only in cases when the intermediate is also fluorescent.

In most cases, high resolution fluorescence microscopy reactivity measurements require deposition of the catalyst on a support. The nanoscale dimensions of heterogeneous catalysts and the fact that the catalytic reaction often occurs when the catalyst is supported, enabled probing the reactivity of supported heterogeneous catalyst without significantly hampering their reactivity. Homogeneous catalysts, on the other hand, are mostly solvated. Anchoring a homogeneous catalyst to a support for probing its reactivity will lower its degrees of freedom and structural flexibility, and these two parameters can have crucial impact on the catalytic process. Hence, correlating the reactivity of supported and solvated homogeneous catalysts is not trivial. One approach to mitigate this limitation was by spatially confining the homogeneous catalyst in a protein pore^154^ or in a polymer aggregate, which is formed during polymerization reaction.^17,18,20,149^ In these two examples, the homogeneous catalyst is relatively flexible and solvated during the reaction, thus the reactivity pattern is expected to be similar to the one observed for the noncoordinated homogeneous catalyst.

### 6.2 Comparative Analysis of Methods for High Spatial Resolution Reactivity Analysis

As demonstrated in the previous sections, high resolution measurements provide unique information about the nanometer-scale factors that govern the catalytic reactivity and the reaction mechanism of homogeneous and heterogeneous (electro)catalysts. However, the harnessed information is governed by the specific measurement method and its technical limitations. Table 1 summarizes the main methods that were described in this review along with their main advantages and limitations.

**Table 1:** Summary of Measurement Techniques Discussed in This Review

method	detection	spatial resolution	advantages	limitations
fluorescence microscopy	product (intermediate in some cases)	depends on the experimental setup	- single molecule detection	- requires light emitting molecules
			- high spatiotemporal resolution	- high sensitivity to physical conditions
			- quantitative information	- no structural information
				- limited field of view

TERS	reactants, products, and stable intermediates	3–20 nm	- direct detection of chemical information	- requires Raman active vibrations
			- ability to detect electronic properties in oxide substrate	- limited temporal resolution
				- requires surface-anchored probes
				- high impact of tip and surface properties on signal

AFM-IR	reactants, products, and stable intermediates	10–20 nm	- direct detection of chemical information	- limited temporal resolution
			- improved reproducibility	- requires surface-anchored probes

SECM/SECCM	electrocatalytic process	>15 nm	- localized electrochemical information	- no direct chemical information

The superb detection capability of single light-emitting events by fluorescence microscopy has led to its utilization in catalysis research for gaining mechanistic insights through analyzing fluorescence trajectories. The most obvious restriction in this method is the requirement for light emitting molecules, either as the product (in most cases) or as the intermediate. Some correlations between the reactivity pattern probed by fluorescence model reactions and the reactivity of nonfluorescence reactions were demonstrated.^155^ Overcoming this limitation is possible by implementing competition between nonfluorescent and fluorescent probes, in which the decay in the signal of fluorescent probes was correlated to surface-induced catalytic reactions.^123^ Yet, one must keep in mind that the signal decay can be also influenced by adsorption of reactants that do not react or by surface diffusion of reactants. Another approach for mitigating this limitation is by tagging reactant molecules with fluorophores. This approach is useful for detecting adsorption sites^116^ or when the reaction triggers a signal, for example, when product molecules precipitate to the focal plane.^17,18,20,149^

Another major limitation in fluorescence microscopy is the demand to work at low concentration (typically nM) to spot a single turnover event that is isolated from other events in time and space. Catalytic reactions are mostly performed at much higher concentrations. The difference in reactants’ concentration between realistic systems and fluorescence microscopy model systems can lead to kinetic effects that will change the reactivity pattern, and therefore fluorescence microscopy experiments may be limited in their ability to serve as a representative model system. In addition, because the method involves fluorescent molecules, fluorescence microscopy is highly sensitive to physical and chemical conditions like temperature and pH. The impact of concentration on the reactivity pattern can be potentially analyzed by mixing a low concentration of fluorescence reactants with high concentration of nonfluorescence reactants that will compete on the same active sites.

Scanning probe microscopy (SPM) spectroscopic methods, such as TERS and AFM-IR, have proved to be informative tools in high spatial resolution research of catalytic systems. In these methods, the spatial resolution is mostly dictated by the tip diameter and hence can ultimately reach the 1 nm range, although in most cases the probed resolution is within 10–20 nm. Interestingly, a study in which TERS-STM measurements led to ∼3 nm resolution was recently reported.^56^ SPM-based measurements with sharper tips can potentially lead to higher spatial resolution but will also require longer acquisition time that can cause deteriorated resolution due to thermal drift.

Additional limitations of SPM-based methods rise from the requirement for using chemically adsorbed reactive probe molecules, such as thiols or NHCs. Probe molecules can theoretically be adsorbed on all active sites, and realistic reaction conditions can be applied. However, this model system enables in most cases one-turnover reactions and therefore yield qualitative reactivity pattern. It should be noted that although the surface density of probe molecules can be high, it was identified that the presence of surface-anchored molecules does not block the adsorption and activation of small molecules.^156^ Moreover, the strong interaction between the probe molecule and the surface enabled to monitor chemical reactions under elevated temperature and various gas-phase conditions while minimizing desorption or diffusion of the probe molecules. The use of chemically active, surface-anchored probe molecules can hinder or limit the flexibility of the chemically active group and its proximity to the surface. It was recently demonstrated that surface proximity plays a dominant role in surface-induced activation of chemically active groups.^157−159^ Therefore, the connection between the reactivity pattern of surface-anchored molecules and real catalytic reactions should be carefully assessed.

The recorded information in tip-based methods might be also influenced by the measurement apparatus. TERS signal is dramatically influenced by tip properties, and therefore tip deformation during measurements or between measurements can impact the signal and lead to deteriorated reproducibility. Sample and tip illumination during TERS measurements can heat the tip and induce physical distortions and degradation.^160^ Various approaches were developed to mitigate this effect, by for example tip coating.^161^ Raman spectroscopy is inherently more sensitive to symmetric molecular vibrations. This dependency indicates that polar bonds, such as C–O, C=O, and O–H, which are interesting from the perspective of oxidation and hydrogenation reaction, will have a relatively small change in their polarizability and hence will be weak Raman scatterers.^57^ It should be noted as well that the close surface–tip proximity can influence the reactivity by tip-induced activation of reactants. This can be mitigated by coating the tip with chemically inert oxides^157^ or by minimizing the residence time of the tip near the surface.

AFM-based IR nanospectroscopy measurements have lower signal in comparison to that obtained by TERS, but due to differences in their surface-enhancement mode, the acquired signal in AFM-IR measurements is less affected by changes in the tip structure.^162,163^ The specific measurement mode can also impact the harnessed signal. For example, due to its field-enhancement dependence, the s-SNOM measurement is more sensitive to dipoles that are oriented perpendicular to the surface, while adsorption geometry will have lower impact on the acquired AFM-IR signals.^164^

SECM/SECCM have proved as extremely efficient techniques to study the electrocatalytic reactivity of nanomaterials and interfaces with an impressive high-spatial-resolution capability that is comparable to the above-mentioned tip-based techniques. These electrochemical methods are sensitive to minute variations in current and/or potential across different morphologies or compositions. Both SECM and SECCM enable applying a potential bias on the studied surfaces, indicating that the stability and reactivity can be measured over various reaction conditions. Notably, there is a growing use of SECCM measurements to visualize complex nanostructured electrocatalysts under reaction conditions, showcasing a new possible direction for future *operando* electrocatalytic studies. However, the complexity of SECCM measurements has limited its implementation; for instance, the use of nanopipettes requires the practices of highly trained electrochemists. In addition, a careful selection and examination of the electrolyte and mediator must be made to avoid competing reactions or side products formation on the surface. Finally, an inherent limitation of SECCM is the lacking capacity to track chemical reactions that do not involve redox processes and the absence of chemical detection, which is needed for probing various prodcuts formation. Overcoming this limitation is necessary in order to obtain the full chemical and electrochemical activity of a probed surface.

### 6.3 Future Perspective

The abovementioned limitations call for multimethod reactivity analysis, which can be achieved either by integration of different methods in one setup or by locating a specific and highly stable particle and tracing its properties using various measurement tools. The unique insights that can be gained by multimethod data integration were recently demonstrated. SPM based methods simultaneously collect topography data and spectroscopic data, and therefore can directly correlate structure and reactivity with a resolution of 10–20 nm. Structure–reactivity correlation at higher resolution were achieved by integration of fluorescence microscopy and TEM or SEM to analyze the properties of zeolites and the impact of structure–composition variations on their reactivity.^130^ The influence of the crystallinity of a single Pd particle on its hydrogen dissociation and sorption affinity were detected by measuring single-particle plasmonic properties and X-ray diffraction on the same nanoparticle in two different setups.^165^ In a similar way, SPM-based microspectroscopy measurements and HR-TEM measurements can be integrated by using a thin graphene layer as substrate for catalytic nanoparticles.^166^

High spatial resolution measurements were mostly performed under conditions that differ from the real-world catalytic reaction, these include low reactants concentration, low temperature and gas pressures, and single turnover measurements with surface-anchored probe molecules. These limitations create a gap between the model system studies and the realistic reaction conditions. In order to bridge this gap, it is necessary to develop new tools and approaches that will enable performing high spatial resolution studies under relevant reaction conditions.

The feasibility for conducting high spatial resolution reactivity measurements under flow of H_2_ was recently demonstrated in studying Mg to MgH_2_ transformation.^79^ Recent examples for IR nanospectroscopy measurements under liquid phase conditions enabled the detection of the molecular structure of graphene–liquid interfaces with nanoscale spatial resolution.^166^ In this setup, a graphene layer was used as a seal for liquid electrolyte reservoir while acting also as a working electrode. The photon transparency of graphene enables IR spectroscopy studies of its interface, thus making it possible to determine changes in speciation and ion concentration in the electric double layers as a function of bias.

Recent developments and applications of SECM/SECCM to map electrochemical reactions on surfaces^167^ have positioned these techniques and especially SECCM as the leading choice for nanoscale probing of the electroactivity of nanomaterials.^168^ Various opportunities and challenges remain in the fields of materials science and electrochemistry in which structure–activity correlations are ever-increasingly needed. *Operando* probing of energy-related materials such as Li–S batteries or conductive polymers are interesting avenues for studying charge transfer mechanisms and structural deactivation pathways. Most recent advancements in the field provided a stimulating glance into correlative microscopies, such as scanning-tunneling X-ray microscopy (STXM) for *in-situ*/*operando* that can complement the high spatial resolution electrochemical data with critical chemical information about the elemental composition of the surface and its corresponding oxidation states.^111^ Future endeavors to further complement SECCM with additional spectroscopic and microscopic techniques are already underway; of note is the coupling interference reflectance microscopy (IRM) to study the *in situ* growth of organic crystals.^169^ However, in order to study catalytic surfaces under reactions conditions, the temporal resolution offered by SECCM would have to be improved. These technical developments will be of key importance for acquiring a detailed mapping of complex materials that will include both chemical and electrochemical information to facilitate a comprehensive understanding of structure–reactivity correlations in several dimensions.
